# Plant proximity perception dynamically modulates hormone levels and sensitivity in *Arabidopsis*

**DOI:** 10.1093/jxb/eru083

**Published:** 2014-03-08

**Authors:** Jordi Bou-Torrent, Anahit Galstyan, Marçal Gallemí, Nicolás Cifuentes-Esquivel, Maria José Molina-Contreras, Mercè Salla-Martret, Yusuke Jikumaru, Shinjiro Yamaguchi, Yuji Kamiya, Jaime F. Martínez-García

**Affiliations:** ^1^Centre for Research in Agricultural Genomics (CRAG), CSIC-IRTA-UAB-UB, Bellaterra, 08193-Barcelona, Spain; ^2^RIKEN Plant Science Center, Yokohama, Kanagawa 230-0045, Japan; ^3^Institució Catalana de Recerca i Estudis Avançats, 08010-Barcelona, Spain

**Keywords:** *Arabidopsis*, auxins, brassinosteroids, gibberellins, hypocotyl elongation, plant proximity, shade avoidance syndrome.

## Abstract

The *s*hade *a*voidance *s*yndrome (SAS) refers to a set of plant responses initiated after perception by the phytochromes of light enriched in far-red colour reflected from or filtered by neighbouring plants. These varied responses are aimed at anticipating eventual shading from potential competitor vegetation. In *Arabidopsis thaliana*, the most obvious SAS response at the seedling stage is the increase in hypocotyl elongation. Here, we describe how plant proximity perception rapidly and temporally alters the levels of not only auxins but also active brassinosteroids and gibberellins. At the same time, shade alters the seedling sensitivity to hormones. Plant proximity perception also involves dramatic changes in gene expression that rapidly result in a new balance between positive and negative factors in a network of interacting basic helix–loop–helix proteins, such as HFR1, PAR1, and BIM and BEE factors. Here, it was shown that several of these factors act as auxin- and BR-responsiveness modulators, which ultimately control the intensity or degree of hypocotyl elongation. It was deduced that, as a consequence of the plant proximity-dependent new, dynamic, and local balance between hormone synthesis and sensitivity (mechanistically resulting from a restructured network of SAS regulators), SAS responses are unleashed and hypocotyls elongate.

## Summary

Shade perception involves altered hormone synthesis and sensitivity. Here, we showed that several shade regulators act as positive and negative modulators of the hypocotyl auxin and/or brassinosteroid-induced elongation.

## Introduction

When plants grow in crowded communities, i.e. in close proximity to other plants, light might become limiting. Under these conditions, they initiate a set of responses, known as the shade avoidance syndrome (SAS), which aim to adapt plant growth and development. SAS responses are observed in both natural (forests, prairies) and agricultural (crop fields, orchards) communities. The presence of nearby plants results in a reduction in the red light to far-red light (R:FR) ratio caused by a specific enrichment in FR reflected from the surface of neighbouring leaves or filtered through them. For shade-intolerant plants, such as *Arabidopsis thaliana*, perception of plant proximity evokes SAS responses that allow the plant to anticipate shading, avoiding it by overgrowing neighbouring plants or by flowering to ensure the production of viable seeds for the next generation. Indeed, responding plants grow away from neighbours well before those putative competitors diminish their actual acquisition of light (Martinez-Garcia *et al.*, 2010; Casal, 2012; Hornitschek *et al.*, 2012; Pierik and de Wit, 2014).

The R:FR ratio changes associated with plant proximity are detected by the phytochrome photoreceptors, which, in *Arabidopsis*, are encoded by a small gene family of five members (*PHYA*–*PHYE*) (Bae and Choi, 2008). These photoreceptors detect mainly the R and FR wavelengths of the light spectrum and exist in two photoconvertible forms, an inactive R-absorbing Pr form and an active FR-absorbing Pfr form. In green plants (i.e. fully de-etiolated), nuclear and active phytochromes orchestrate a transcriptional network in part by interacting with different *P*HYTOCHROME *I*NTERACTING *F*ACTORs (PIFs) (Leivar and Quail, 2011; Martinez-Garcia *et al.*, 2000a), which involves rapid changes in *PHYTOCHROME RAPIDLY REGULATED* (*PAR*) gene expression.

Several *PAR* genes have been shown to be instrumental for implementing the morphological and physiological SAS responses, including members from three different families of transcription factors: (i) the homeodomain-leucine zipper (HD-ZIP) class II subfamily (*ATHB2*/*HAT4*; hereafter *ATHB2*, *ATHB4*, *HAT1*, *HAT2*, and *HAT3*) (Steindler *et al.*, 1999; Sorin *et al.*, 2009); (ii) the basic helix–loop–helix (bHLH) family (Salter *et al.*, 2003; Sessa *et al.*, 2005; Roig-Villanova *et al.*, 2006, 2007; Galstyan *et al.*, 2011, 2012; Cifuentes-Esquivel *et al.*, 2013); and (iii) the *B-BOX-CONTAINING* (*BBX*) family (Crocco *et al.*, 2010; Gangappa *et al.*, 2013). Analyses of mutants with altered activity of these factors led us to propose negative [*LONG HYPOCOTYL IN FAR RED1* (*HFR1*); *PAR1*, *PAR2, PIF3-LIKE1* (*PIL1*), *BBX21* and *BBX22*], positive [*BR-ENHANCED EXPRESSION* (*BEE*), *BES1-INTERACTING MYC-LIKE* (*BIM*), *BBX24* and *BBX25*] and complex (HD-ZIP II) activities in the regulation of SAS. Genetic analyses have also implicated non-PAR factors in the regulation of SAS responses, such as HD-ZIP class III transcription factors (Bou-Torrent *et al.*, 2012; Brandt *et al.*, 2012), growth-repressing DELLA proteins and *CONSTITUTIVE SHADE AVOIDANCE 1* (*CSA1*) (Faigon-Soverna *et al.*, 2006; Djakovic-Petrovic *et al.*, 2007). In addition, PIF1, PIF3, PIF4, PIF5 (called the PIF quartet), and PIF7, also proteins of the bHLH subfamily, were identified as SAS positive players that participate in the expression regulation of some *PAR* genes, such as *ATHB2*, *HFR1*, or *PIL1*; in contrast with *PAR* genes, PIF expression is unaffected by plant proximity, but the stability of the resulting proteins is increased by this light signal (Lorrain *et al.*, 2008; Leivar *et al.*, 2012; Li *et al.*, 2012). Recently, it was suggested that the distinct genetic components known to participate in the SAS transcriptional network are organized, forming functional modules, such as PIFs–HFR1, BEEs–PAR1, BIMs and ATHBs–HATs, that control specific gene sets (Cifuentes-Esquivel *et al.*, 2013).

All plant hormones that are involved in elongation growth, such as auxins, brassinosteroids (BRs), and gibberellins (GAs), are potential actors in SAS elongating responses (Martinez-Garcia *et al.*, 2010). Indeed, since the pre-molecular era, it has been accepted (although with some controversy) that the control of hormone-triggered responses might be exerted by concentration changes, by alterations in tissue sensitivity, or by a combination of both (Cleland, 1983). Similarly, light treatments were postulated to alter hypocotyl/stem elongation by modifying these two aspects of hormone action: levels and sensitivity (Kende and Lang, 1964; Kamiya and Garcia-Martinez, 1999; Alabadi and Blazquez, 2009). However, the molecular basis of how changes in hormone sensitivity are instrumented after plant proximity perception remains almost unexplored. In this respect, some of the mentioned SAS regulators might be entry points for the shade signal perceived by the phytochromes intersecting with those regulating cell division and expansion, such as the ones controlled by plant hormones, to adapt the pattern of development to plant proximity (Nemhauser, 2008; Halliday *et al.*, 2009; Martinez-Garcia *et al.*, 2010). PAR1, found to directly repress two auxin-responsive genes, *SAUR15* and *SAUR68*, was proposed to integrate shade- and auxin-mediated transcriptional networks, rapidly connecting phytochrome-sensed light changes with auxin responsiveness (Roig-Villanova *et al.*, 2007). ATHB4, which also regulates the expression of several auxin-, BR-, and/or shade-induced genes, such as *SAUR15*, *SAUR68*, *HAT2*, and *IAA1*, provides additional entry points by which shade- and hormone-regulated transcriptional networks are integrated (Sorin *et al.*, 2009). More recently, PIF5 and PIF7 were shown to directly regulate auxin synthesis or signalling for shade-induced growth (Hornitschek *et al.*, 2012; Li *et al.*, 2012), providing a mechanism by which shade perception by phytochromes results in the accumulation of new auxin in seedlings for the early hypocotyl elongation. In seedlings growing under short-day photoperiods, PIF4 and PIF5 were also shown to modulate auxin sensitivity, reinforcing a shared role for PIFs in modulating auxin pathways in light-regulated elongation responses (Nozue *et al.*, 2011). Plant proximity was also shown to rapidly increase endogenous levels of bioactive auxin (free indole-3-acetic acid, IAA), an effect that involved the action of *SHADE AVOIDANCE 3* (*SAV3*)/*TRYPTOPHAN AMINOTRANSFERASE OF ARABIDOPSIS 1* (*TAA1*) (Tao *et al.*, 2008), and alter PIN-FORMED 3 (PIN3) protein, a regulator of auxin efflux, in seedlings (Keuskamp *et al.*, 2010). Canopy shade was also reported to induce the auxin-regulated *AtCKX6* gene, involved in promoting cytokinin breakdown and resulting in an arrest in leaf primordium growth, which ensures that plant resources are redirected into extension growth (Carabelli *et al.*, 2007). Overall, there are multiple contact points between shade signalling and hormone synthesis and signalling that might be part of the mechanisms that link shade perception to the actual changes in plant growth and physiology.

In this study, it was shown that, under simulated shade, hypocotyl elongation is mediated by changes in both hormone levels and hormone sensitivity. Our work indicates that plants avoid shading by dynamically altering the levels of hormones but also of transcription regulators involved in locally altering hormone sensitivity, providing a framework to understand further how plant proximity perception results in the differential growth associated with SAS responses.

## Material and methods

### Plant material and growth conditions

*Arabidopsis* (*A. thaliana*) mutants and transgenic lines used in this study were in the Columbia (Col-0) background. The lines overexpressing *PAR1* fused to the green fluorescent protein gene (*GFP*) (P35S:PAR1-G.01) or to the β-glucuronidase (*GUS*)–*GFP* double reporter (P35S:PAR1-GG.13), *PAR2* alone (P35S:PAR2.12) or fused to *GFP* (P35S:PAR1-G.03), transgenic PAR1-RNAi, mutant *par2-1* (Roig-Villanova *et al.*, 2007), lines overexpressing truncated forms of *HFR1* fused to *GFP* (P35S:G-BH.03 and P35S:G-H.02), mutant *hfr1-5* (Galstyan *et al.*, 2011), and triple mutants *bee123* and *bim123* (Cifuentes-Esquivel *et al.*, 2013) have been described elsewhere. For seed production and crosses, plants were grown in the greenhouse under long-day conditions. Seeds were surface sterilized and sown on Petri dishes with solid growth medium without sucrose (GM–) (Roig-Villanova *et al.*, 2007). After stratification (3–6 d), the plates were incubated in growth chambers at 22 °C under continuous white light (W, 25 µmol m^–2^ s^–1^ of photosynthetically active radiation; R:FR ratio >2.1). Simulated shade (W+FR) was generated by enriching W with supplementary FR provided by QB1310CS-670–735 LED hybrid lamps (Quantum Devices Inc., http://www.quantumdev.com) (25 µmol m^–2^ s^–1^ of photosynthetically active radiation; R:FR ratio of 0.05). Fluence rates were measured using an EPP2000 spectrometer (StellarNet, http://www.stellarnet-inc.com) (Sorin *et al.*, 2009). For hormone response analyses, seeds were grown in solid GM– supplemented or not with the growth regulators from the day of sowing. For gene expression analyses, seeds were sown on filter paper on top of GM– medium.

### Chemical treatments

To prepare stock solutions, epibrassinolide (EBL; Sigma-Aldrich, http://www.sigmaaldrich.com), gibberellin A_3_ (GA_3_; Sigma-Aldrich), and paclobutrazol (PAC; Duchefa, http://www.duchefa.com) were dissolved in absolute ethanol at 5, 100, and 1mM, respectively. Picloram (PIC; Duchefa) was dissolved in DMSO at 50mM. Propiconazol (PCZ; BannerMax, Syngenta, http://www.syngenta.com) was dissolved in water at 5mM. Stock solutions were kept at –20ºC until use.

### Hypocotyl measurements

The National Institutes of Health ImageJ software (http://rsb.info.nih.gov) was used on digital images to measure the length of different organs of the seedlings, as described elsewhere (Sorin *et al.*, 2009). At least 15 seedlings were used for each data point, and experiments were repeated two to fivetimes and a representative result is shown. Statistical analyses of the data [*t*-test and two-way analysis of variance (ANOVA)] were performed using GraphPad Prism version 4.00 for Windows (http://www.graphpad.com).

### RNA blot analysis

RNA blot analyses and quantification of expression levels were performed as indicated elsewhere (Roig-Villanova *et al.*, 2006). The hybridization probes are described in Supplementary Experimental Procedures available at *JXB* online.

### Microarray data analysis

Published microarray data were used to identify a set of genes that transcriptionally respond after 1h of W+FR treatment in Col-0, *sav1*, and *sav3* seedlings. Published data from Affymetrix microarrays (GEO accession number for the microarray sequence data GSE9816) (Tao *et al.*, 2008) were analysed as described previously (Kaufmann *et al.*, 2010). Briefly, data were imported into the Resolver gene expression data analysis system version 7.1 (Rosetta Biosoftware, http://www.ceibasolutions.com/rosetta-about) and processed as described previously (Wellmer *et al.*, 2006). Resolver uses a platform-specific error model-based approach to stabilize the variance estimation to improve the specificity and sensitivity in differential gene expression detection (Weng *et al.*, 2006). The data from the biological replicates of each condition were combined, resulting in an error model weighted average of the replicates. The *P* values for differential expression calculated by Resolver were adjusted for multi-hypothesis testing using the Benjamini and Hochberg procedure, as implemented in the Bioconductor multtest package in R (http://www.bioconductor.org/packages/bioc/stable/src/contrib/html/multtest.html). Genes for which the Benjamini and Hochberg-adjusted *P* value was <0.05 and an absolute fold-change cut-off of 1.5 were considered differentially expressed in response to 1h of simulated shade treatment. This threshold resulted in the identification of a total of 163, 320, and 160 genes that showed expression changes during the simulated shade treatment in Col-0, *sav1*, and *sav3* seedlings, respectively (Supplementary Table S2 available at *JXB* online). Among these genes, several well-described *PAR* genes, such as *HFR1*, *PAR1*, and *ATHB4*, were found.

### Hormone quantification

Plant hormones except for castasterone (CS) were quantified as described elsewhere (Yoshimoto *et al.*, 2009). CS was quantified as indicated in Supplementary Experimental Procedures (available at *JXB* online).

### Accession numbers

Sequence data from this article can be found in the Arabidopsis Genome Initiative database under the following accession numbers: *PAR1* (*At2g42870*), *PAR2* (*At3g58850*), *SAUR15* (*At4g38850*), *SAUR68* (*At1g29510*), *BEE1* (*At1g18400*), *BEE2* (*At4g36540*), *BEE3* (*At1g73830*), *BIM1* (*At5g08130*), *BIM2* (*At1g69010*), *BIM3* (*At5g38860*), *HFR1* (*At1g02340*) and *At5g45670*.

## Results

### Simulated shade perception rapidly alters hormone levels

**Fig. 1.**
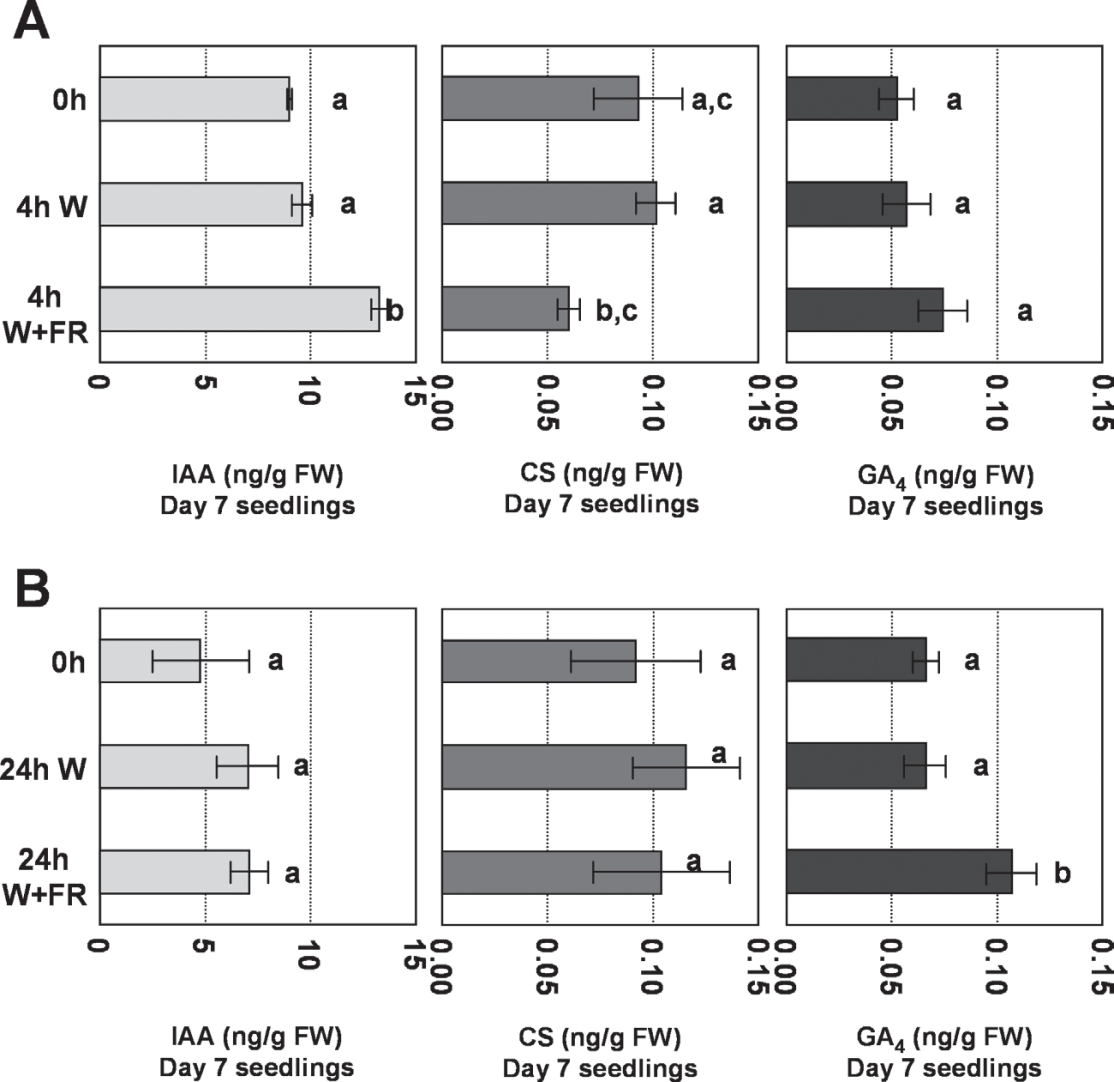
Analysis of hormone levels in wild-type seedlings treated with simulated shade. Seedlings were germinated and grown for 7 d under W and then either kept in W or transferred to W+FR for 4h (A) or 24h (B). Values are means ±standard error (SE) of three experiments; FW, fresh weight. Different letters denote significant differences (*P*<0.01 for IAA levels; *P*<0.05 for CS levels; and *P*<0.1 for GA_4_ levels) among means.

Several of the identified SAS regulators, such as ATHB2, ATHB4, BEEs, and BIMs, have previously been related to hormone signalling, an observation consistent with the idea that promotion of hypocotyl elongation in response to simulated shade is mediated by changes in both hormone levels and hormone sensitivity (Kamiya and Garcia-Martinez, 1999; Alabadi and Blazquez, 2009). To test these possibilities, it was first addressed whether plant proximity perception could alter the levels of different growth-promoting hormones in a rapid and sustained manner. We focused on auxins, BRs, and GAs. Seedlings grown for 5 or 7 d under continuous W were treated either with W or W+FR (simulated shade) and samples were collected after 4 or 24h. Short-term (4h) simulated shade treatments resulted in higher levels of the auxin IAA but lower levels of the active BR CS (Fig. 1A and Supplementary Fig. S1 available at *JXB* online). The rapid increase in IAA levels is consistent with published information (Tao *et al.*, 2008; Keuskamp *et al.*, 2010; Hornitschek *et al.*, 2012). However, longer periods (24h) abolished the differences in IAA and CS levels between W and W+FR treatments (Fig. 1B), suggesting that simulated shade altered auxin and BR levels in the seedling in a dynamic fashion. By contrast, simulated shade treatments resulted in a mild but sustained increase in the levels of GA_4_, the major bioactive GA in *Arabidopsis* (Fig. 1 and Supplementary Fig. S1 available at *JXB* online). Overall, the observed shade-modulated and dynamic changes in IAA, CS, and GA_4_ levels, although modest, provide a hormonal basis for the resulting induction of hypocotyl elongation.

### Simulated shade perception alters hormone responsiveness

**Fig. 2.**
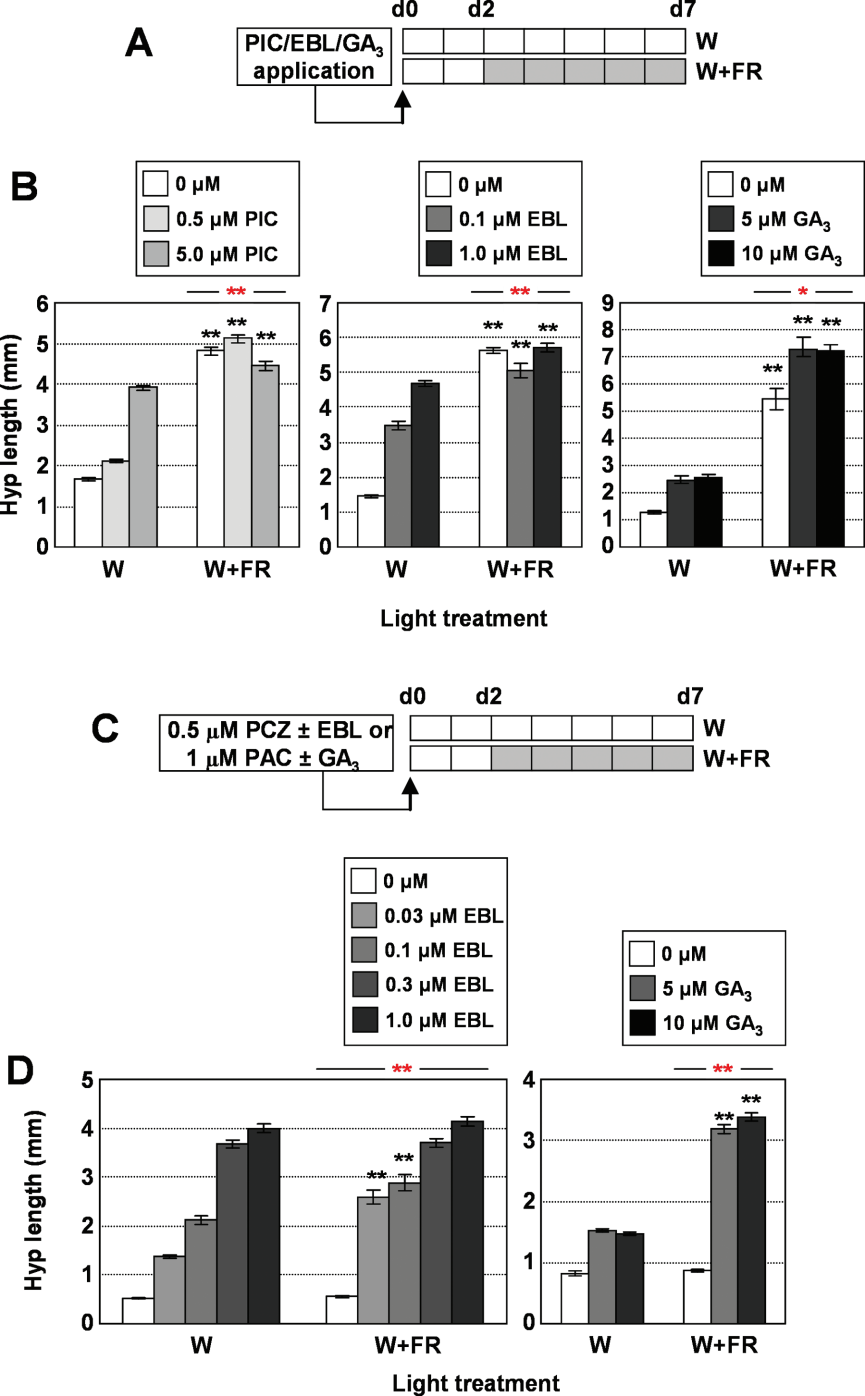
Effect of simulated shade on the hypocotyl response to hormone application. Wild-type seedlings were germinated and grown for 2 d under W and then either kept in W or transferred to W+FR for 5 more days. (A) Medium was supplemented with different concentrations of PIC, EBL, or GA_3_. (B) Hypocotyl length of seedlings grown as depicted in (A) was measured for each treatment. (C) Medium was supplemented with 0.5 µM of PCZ or 1 µM PAC with different concentrations of EBL or GA_3_. (D) Hypocotyl length of seedlings grown as depicted in (C) was measured for each treatment. In (B) and (D), values are means ±SE. Black asterisks indicate significant differences (Student’s *t*-test) relative to the corresponding W-grown controls; red asterisks indicate significant differences (two-way ANOVA) between W- and W+FR-grown seedlings in response to the corresponding hormone applied (*P<0.05, **P<0.01). (This figure is available in colour at *JXB* online.)

To address whether simulated shade also affected seedling sensitivity to these hormones in our specific conditions, hypocotyl elongation was measured in wild-type seedlings treated with bioactive auxins, BRs, or GAs (Fig. 2). Because IAA, the naturally occurring form of auxin, poorly promotes hypocotyl elongation when supplied exogenously, we employed PIC, a synthetic auxin analogue that phenocopies the genetic increase of endogenous auxin levels and is not metabolized by the plant tissues (Horton and Fletcher, 1968; Delarue *et al.*, 1998; Sorin *et al.*, 2005; Vert *et al.*, 2008). Commercially available EBL and GA_3_ were used as BRs and GAs, respectively, because they are bioactive when exogenously applied in a range of plants, including *Arabidopsis* (Alabadi *et al.*, 2004; de Lucas *et al.*, 2008; Hartwig *et al.*, 2012). Under continuous W, all three compounds stimulated hypocotyl elongation in a dose-dependent manner, GA_3_ being the less active in promoting growth. Under sustained W+FR conditions, only GA_3_ promoted hypocotyl elongation at both doses applied, whereas PIC and EBL had subtle inhibitory effects at different doses (Fig. 2A, B). Two-way ANOVA tests showed that wild-type hypocotyls responded differently to PIC (*P*<0.01), EBL (*P*<0.01), and GA_3_ (*P*<0.05), depending on the light conditions (W vs W+FR) (Supplementary Table S1 available at *JXB* online). To provide deeper insights into the effect of simulated shade on hypocotyl sensitivity to BRs and GAs, EBL and GA_3_ were applied to seedlings treated with PCZ and PAC, respectively. PCZ is a fungicide described recently as an inhibitor of BR biosynthesis in both *Arabidopsis* and maize seedlings (Hartwig *et al.*, 2012). PAC is a well-known inhibitor of endogenous GA biosynthesis (Martinez-Garcia and Garcia-Martinez, 1992; Alabadi *et al.*, 2004). As expected, in the absence of EBL or GA_3_, PCZ- and PAC-treated seedlings were almost unresponsive to simulated shade. Under W and W+FR, both EBL and GA_3_ stimulated hypocotyl elongation in a dose-dependent manner, although simulated shade-treated seedlings showed a hypersensitive response to both compounds (Fig. 2C, D). Two-way ANOVA tests showed that W+FR-treated hypocotyls responded significantly more to EBL (*P*<0.01) and GA_3_ (*P*<0.01) (Supplementary Table S1 available at *JXB* online). Together, these results indicate that simulated shade significantly alters hypocotyl responsiveness to auxins, BRs and GAs, probably reflecting changes in the sensitivity to the endogenous active hormones.

**Fig. 3.**
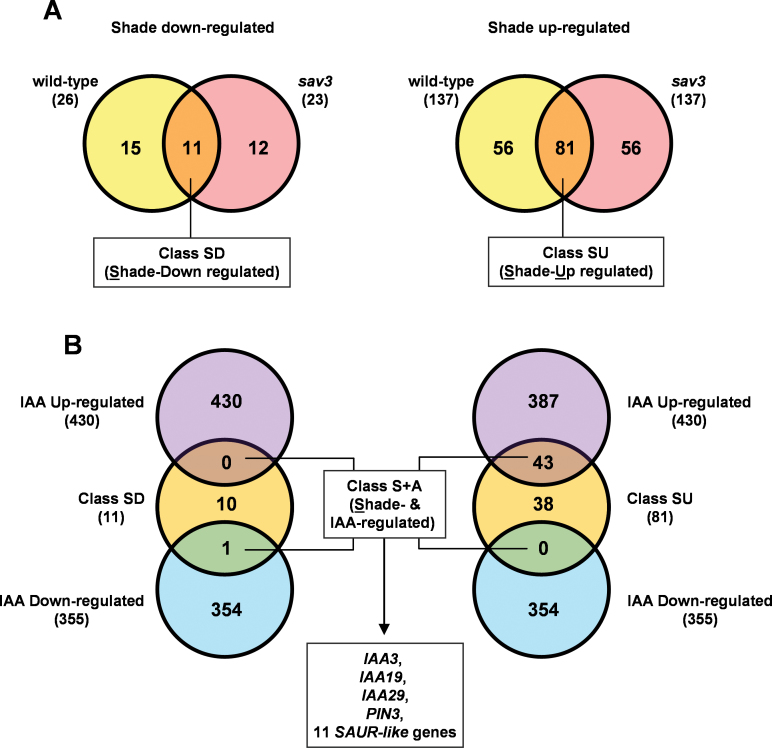
Merging of microarray data from shade-regulated and IAA-regulated genes. (A) Venn diagrams illustrating the overlap between the rapidly downregulated (left) and upregulated (right) group of genes in wild-type and *sav3* mutant seedlings in response to 1h of simulated shade (Tao *et al.*, 2008), as listed in Table S2. The total number of genes in each group is indicated in parentheses. Comparisons between genotypes defined two classes of robust shade-regulated genes: shade downregulated (class SD) and shade upregulated (class SU), as listed in Table S3. The numbers of genes in each sector is indicated. (B) Venn diagrams illustrating the overlap between the class SD (left), class SU (right), and class of IAA upregulated (top) and downregulated (bottom) genes. The comparison between the different groups of genes defines the class S+A (Table S3).

Next, available transcriptomic data were compared to test whether the expression of genes involved in controlling auxin, BR, and GA sensitivity could be altered by simulated shade. As mentioned above, mutant *sav3* seedlings are deficient in the biosynthetic enzyme SAV3/TAA1 required for the rapid increase in free IAA levels after simulated shade perception (Tao *et al.*, 2008). A list of genes significantly regulated by 1h of simulated shade in Col-0 (163 in total, 137 up- and 26 downregulated) and *sav3* (160 in total, 137 up- and 23 downregulated) seedlings were obtained from published data (GEO accession number for the microarray data is GSE9816) (Supplementary Table S2 available at *JXB* online). From these, 92 genes (81 up- and 11 downregulated) were shade regulated in both genotypes, and were named classes SU (*s*hade *u*pregulated) and SD (*s*hade *d*ownregulated) (Fig. 3A, Supplementary Table S3 available at *JXB* online). Comparison of the SU and SD genes with those rapidly up- or downregulated after BL (Supplementary Table 3, available at *JXB* online, in Nemhauser *et al.*, 2006) or GA application (Supplementary Table 4, available at *JXB* online, in Nemhauser *et al.*, 2006) probably reflected the different dynamics of the changes in CS and GA_4_ levels measured: 31 of the 92 shade-regulated genes of the SU class were BL regulated, but only three genes were also GA regulated (overlap was observed only between BL regulated and GA regulated and the SU class; Supplementary Fig. S2, available at *JXB* online). However, this comparison did not provide additional insights on whether BR or GA sensitivity was altered (Supplementary Table S3 available at *JXB* online). By contrast, comparison of these 92 shade-regulated genes with those rapidly up- or downregulated after IAA application (see Supplementary Table 5, available at *JXB* online, in Nemhauser *et al.*, 2006) indicated that a high proportion of the SU genes (43 out of 81) were also induced by IAA application (*s*hade +*a*uxin, S+A class) (Fig. 3B, Supplementary Table S3 available at *JXB* online). Because in *sav3* there is no increase in free IAA levels after simulated shade (Tao *et al.*, 2008), it is inferred that, in simulated shade-treated seedlings, the S+A class of genes is regulated by shade but is independent of the associated (shade-triggered) increase in IAA levels. Several of the S+A genes encode for genes with a role in auxin signalling, such as IAA (IAA3, IAA19, and IAA29), PIN3, and 11 SAUR-like auxin-responsive proteins that probably contribute to the observed changes in auxin sensitivity of the shade-grown seedlings (Fig. 3B, Supplementary Table S3, available at *JXB* online) (Calderon Villalobos *et al.*, 2012). Altogether, these data indicated that simulated shade induces complex but dynamic changes in both the production of different hormones and the seedling responsiveness to these hormones at both molecular and physiological levels.

### HFR1 has little effect on hypocotyl responsiveness to auxins or BRs in white light

**Fig. 4.**
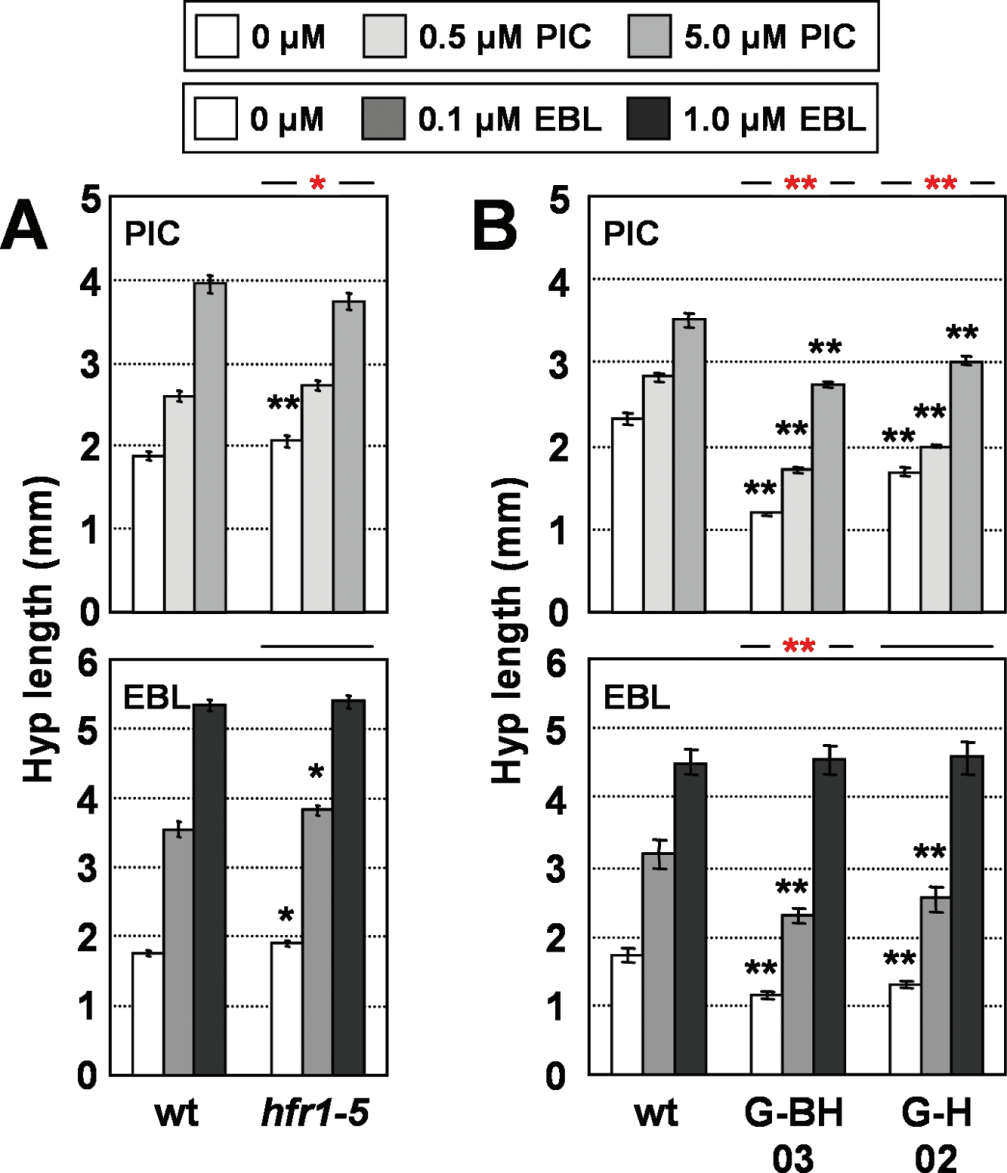
Hypocotyl response of seedlings with altered levels of HFR1 to hormone application. Hypocotyl length of wild-type (wt), *hfr1-5* (A) or truncated *HFR1* overexpressor (B) seedlings germinated and grown under W for 7 d on medium supplemented with increasing concentrations of PIC (upper panels) or EBL (lower panels). Hypocotyl length was measured for each line and treatment. Values are means ±SE. Black asterisks indicate significant differences (Student’s *t*-test) relative to the corresponding wild-type plants; red asterisks indicate significant differences (two-way ANOVA) between genotypes in response to the corresponding hormone applied (*P<0.05, **P<0.01). (This figure is available in colour at *JXB* online.)

*HFR1* is a SAS negative regulator whose expression is rapidly induced by simulated shade. To test whether HFR1 could modulate the seedling sensitivity to these hormones, hypocotyl elongation in response to PIC and EBL was compared in W-grown lines with altered HFR1 activity. Although the loss-of-function *hfr1-5* mutant displays an increased response to simulated or canopy shade (Sessa *et al.*, 2005; Roig-Villanova *et al.*, 2007), an attenuated hypocotyl response to PIC and a wild-type hypocotyl response to EBL of mutant seedlings was observed (Fig. 4, Supplementary Table S4 at *JXB* online). To analyse hormone responsiveness in gain of HFR1 function, available transgenic lines producing truncated HFR1 derivatives fused to GFP were employed: G-BH, which lacked the whole aa 1–131 N-terminal region (P35S:G-BH plants), and G-H, with only the HLH domain and the adjacent C-terminal region (P35S:G-H plants) (Supplementary Fig. S3 available at *JXB* online). Both proteins strongly inhibited the hypocotyl response to W+FR. In particular, hypocotyls of the P35S:G-BH line were completely unresponsive to simulated shade (Galstyan *et al.*, 2011). In contrast to this reduced response to simulated shade treatment, hypocotyls of these transgenic seedlings elongated in response to PIC and EBL at least as much as those of wild-type seedlings (Fig. 4B, Supplementary Table S4 available at *JXB* online). These results indicated that HFR1 has a minor role, if any, in modulating hypocotyl elongation induced by these hormones, i.e. HFR1 does not seem to control auxin or BR responsiveness.

### High levels of PAR1 and PAR2 reduce the auxin responsiveness of hypocotyls in white light

**Fig. 5.**
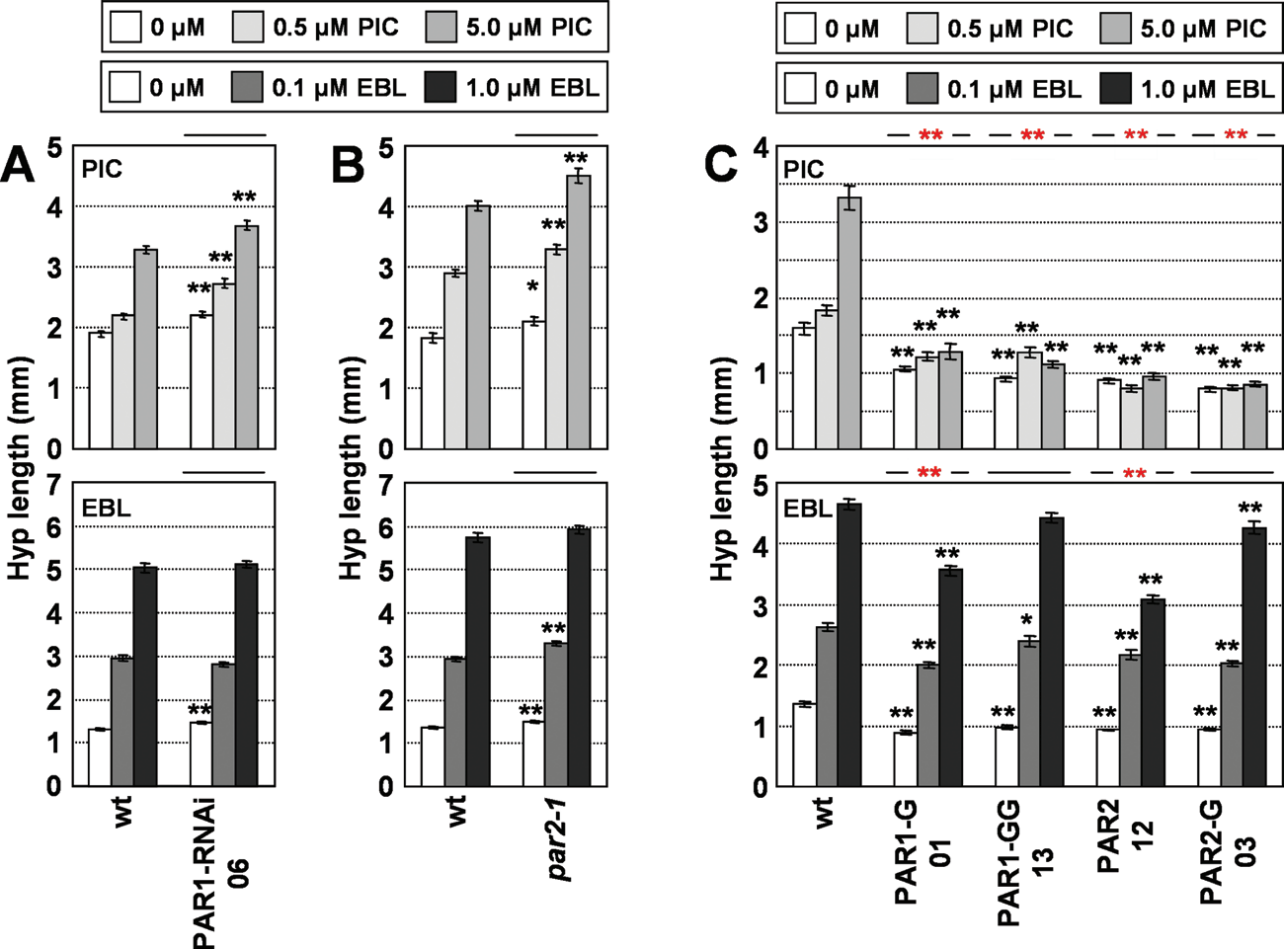
Hypocotyl response of seedlings with altered levels of PAR1 or PAR2 to hormone application. Hypocotyl length of wild-type (wt), PAR1-RNAi (A), *par2-1* (B), or *PAR1* and *PAR2* overexpressor (C) seedlings germinated and grown under W for 7 d on medium supplemented with increasing concentrations of PIC (upper panels) or EBL (lower panels). Hypocotyl length was measured for each line and treatment. Values are means ±SE. Black asterisks indicate significant differences (Student’s *t*-test) relative to the corresponding wild-type plants; red asterisks indicate significant differences (two-way ANOVA) between genotypes in response to the corresponding hormone applied (*P<0.05, **P<0.01). (This figure is available in colour at *JXB* online.)

A role for PAR1 and PAR2 as modulators of hormone responsiveness was suggested based on the altered expression of some *SAUR* genes involved in auxin and BR signalling (Roig-Villanova *et al.*, 2007). To further substantiate their role as modulators of seedling sensitivity to these hormones, hypocotyl elongation in response to PIC and EBL was compared in W-grown lines with reduced or increased *PAR1* or *PAR2* levels (Roig-Villanova *et al.*, 2007). The response to hormone treatment of PAR1-RNAi and *par2-1* hypocotyls was essentially identical to that of wild-type seedlings. Hypocotyls of seedlings overexpressing *PAR1* fused to *GFP* (P35S:PAR1-G) or to the *GUS–GFP* double reporter (P35S:PAR1-GG), *PAR2* alone (P35S:PAR2), or fused to *GFP* (P35S:PAR1-G) responded to EBL at least as much as wild-type seedlings (Fig. 5, Supplementary Table S5 available at *JXB* online), despite showing an attenuated EBL-induced expression of specific (*At5g45670*) or non-specific (*SAUR15*, *SAUR68*) BR-regulated genes (Nemhauser *et al.*, 2006; Goda *et al.*, 2008) (Supplementary Fig. S4 available at *JXB* online). By contrast, *PAR1* and *PAR2* overexpressing seedlings were almost unresponsive to the same PIC concentrations that induced hypocotyl elongation in wild-type seedlings (Fig. 5C). Two-way ANOVA tests confirmed the interaction between high levels of PAR1 and/or PAR2 and PIC treatments in terms of hypocotyl elongation (Supplementary Table S5 available at *JXB* online), confirming that PAR1 and PAR2 modulate auxin responsiveness of hypocotyls. Together, these experiments indicated that high levels of *PAR1* and *PAR2* strongly downregulate the hypocotyl elongation response of *Arabidopsis* seedlings to auxins but have little effect on the same response to BRs (EBL).

### BEE and BIM factors differently modulate the auxin and BR responsiveness of hypocotyls in white light

**Fig. 6.**
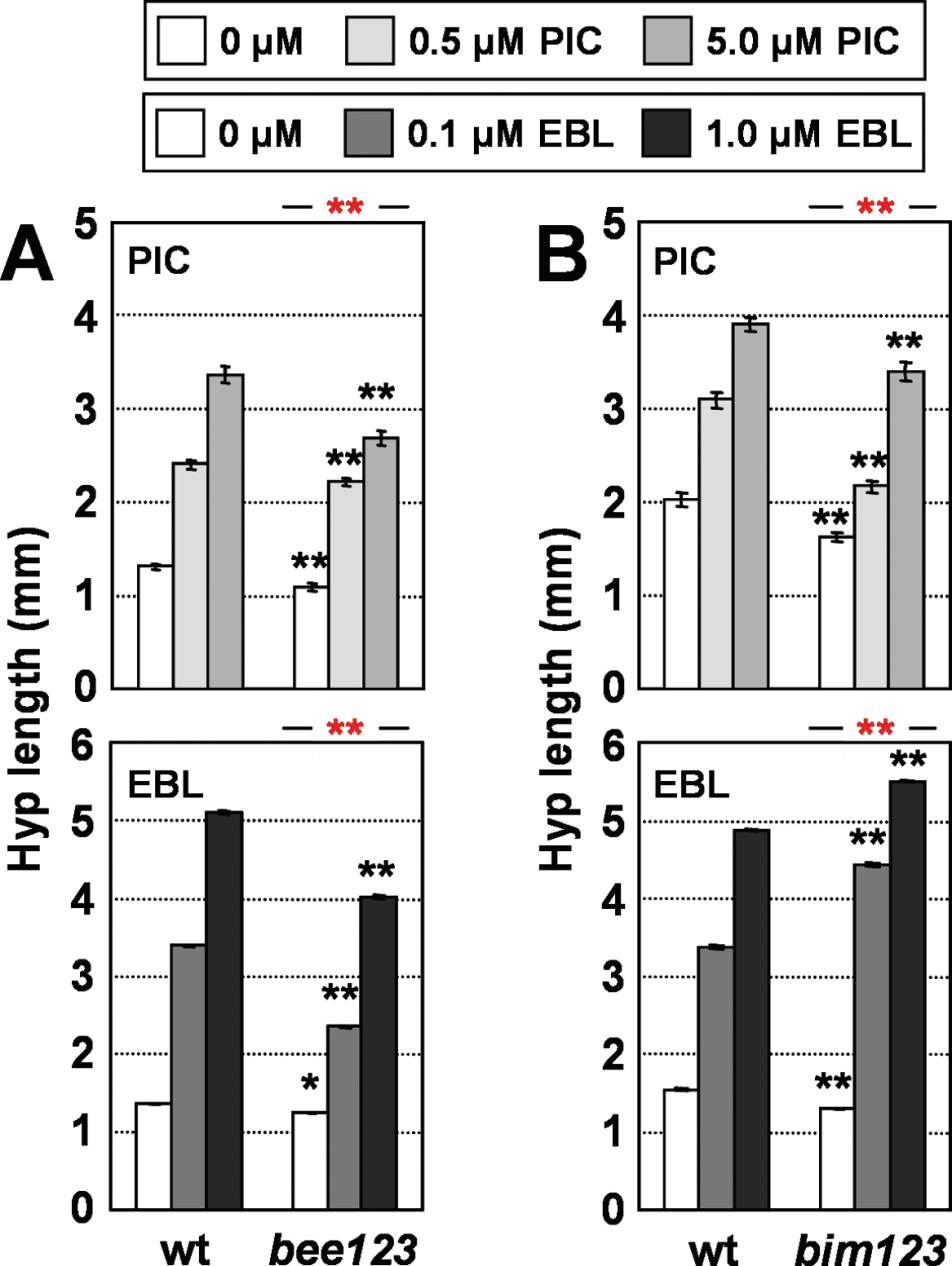
Hypocotyl response of *bee123* and *bim123* mutant seedlings to hormone application. Hypocotyl length of wild-type (wt), *bee123* (A), or *bim123* (B) seedlings germinated and grown under W for 7 d on medium supplemented with increasing concentrations of PIC (upper panels) or EBL (lower panels). Hypocotyl length was measured for each line and treatment. Values are means ±SE. Black asterisks indicate significant differences (Student’s *t*-test) relative to the corresponding wild-type plants; red asterisks indicate significant differences (two-way ANOVA) between genotypes in response to the corresponding hormone applied (*P<0.05, **P<0.01). (This figure is available in colour at *JXB* online.)

The positive SAS regulators BIM and BEE factors were reported previously as positive components of BR signalling (Friedrichsen *et al.*, 2002; Yin *et al.*, 2005). Consistent with these results, triple *bee1 bee2 bee3* (hereafter referred to as *bee123*) mutant seedlings displayed a reduced hypocotyl response to EBL (Fig. 6A, Supplementary Table S6 available at *JXB* online), supporting a positive role for BEE factors as BR-signalling regulators. By contrast, hypocotyl elongation in triple *bim1 bim2 bim3* (hereafter referred to as *bim123*) mutant seedlings was not reduced but increased in response to EBL compared with wild-type seedlings (Fig. 6B, Supplementary Table S6 available at *JXB* online). These results support a role for BIM factors as negative regulators of BR signalling in light-grown seedlings, in clear contrast to the reported positive role deduced from the inhibition of hypocotyl elongation by the BR biosynthesis inhibitor brassinazole in mutant etiolated seedlings (Yin *et al.*, 2005). In any case, our results support a role for BEE and BIM factors in controlling physiological BR responsiveness.

As BEE and BIM factors physically interact with PAR1 (Cifuentes-Esquivel *et al.*, 2013), and PAR1 was shown to reduce auxin responsiveness (Fig. 5), it was next addressed whether BEE and BIM factors affected the response to PIC. As shown in Fig. 6A, mutant *bee123* and *bim123* seedlings displayed a reduced hypocotyl elongation in response to PIC. Two-way ANOVA test confirmed the interaction between *BEE* and *BIM* levels and PIC treatments in terms of hypocotyl elongation (Supplementary Table S6 available at *JXB* online). These results confirmed the participation of BEE and BIM factors in controlling the hypocotyl elongation response to auxins. The opposite effect of these factors relative to PAR1 and PAR2 is consistent with the molecular mechanism described for their interaction, based on the inhibition of BEE and BIM DNA-binding and transcriptional activities upon binding to PAR1 (Cifuentes-Esquivel *et al.*, 2013). Together, our data suggest that different PAR factors with a role as SAS regulators contribute differently to the hypocotyl responsiveness to auxins and BRs.

## Discussion

The SAS includes morphological, physiological, metabolic, and molecular responses that are very probably interconnected. Hypocotyl elongation is one of the SAS responses more frequently studied because of its simplicity. Despite the identification of several factors involved in shade-induced hypocotyl elongation, we still know little about how they are connected with the endogenous mechanisms that boost growth (elongation). Previously, it was shown that auxins and BRs cooperatively promote shade-induced petiole elongation, a different SAS response (Kozuka *et al.*, 2010). In the case of the shade-induced hypocotyl elongation, intact auxin, BR, and GA pathways are required for the normal display of this SAS response (Djakovic-Petrovic *et al.*, 2007; Tao *et al.*, 2008; Martinez-Garcia *et al.*, 2010). Here, it was shown that perception of simulated shade has a relatively rapid impact on the levels of hormones known to stimulate hypocotyl elongation in whole *Arabidopsis* seedlings. The differences, however, might be more dramatic at the cell or organ level, as was shown in other plant systems (Martinez-Garcia *et al.*, 2000b; Weller *et al.*, 1994; Kurepin *et al.*, 2007). A rapid increase in auxin levels has previously been observed as soon as 1h after low R:FR treatment, a process that requires the auxin biosynthetic genes *TAA1*/*SAV3* (Tao *et al.*, 2008) and *YUCCA*. PIF4, PIF5, and PIF7 directly activate the expression of several *YUCCA* genes, providing a direct link between shade perception and auxin synthesis regulation (Hornitschek *et al.*, 2012; Li *et al.*, 2012). Genetic analyses suggest that other bHLH transcription factors, such as BIMs, might also play a direct or indirect role in auxin synthesis via the control of *YUCCA* expression (Cifuentes-Esquivel *et al.*, 2013). We have observed that, whereas IAA and CS levels are rapidly (after 4h) but transitorily (24h) altered, GA_4_ levels steadily increased after simulated shade treatment (Fig. 1 and Supplementary Fig. S1 available at *JXB* online). The dynamic reduction in CS levels observed here in both 5- and 7-d-old seedlings might be caused by its increased inactivation. In agreement, *BAS1*, a gene involved in BR inactivation, is the only gene related to BR metabolism whose expression is activated 1h after low R:FR perception (Supplementary Tables S2 and S7A available at *JXB* online). The increase in GA_4_ levels is supported by the general upregulation of expression of all five *Arabidopsis* genes encoding GA-20 oxidases in seedlings grown under low R:FR conditions for 24h (Supplementary Table S7B available at *JXB* online), an increase that might be linked with the previous increase in IAA levels (Frigerio *et al.*, 2006). These *in silico* observations are consistent with the observed changes in hormone levels.

In contrast to the increase in IAA and GA_4_ levels, the observed transient reduction in CS levels does not have an obvious biological relevance. Indeed, it might even seem contradictory with the promotion of hypocotyl elongation induced by simulated shade, as mutant seedlings defective in genes encoding enzymes involved in BR biosynthesis display an attenuated or null hypocotyl elongation in response to plant proximity or canopy shade. This is the case of *dwarf1-101* (*dwf1-101*) (Luccioni *et al.*, 2002), *de-etiolated2-1* (*det2-1*) (Martinez-Garcia *et al.*, 2010), and *sav1*, this last mutant identified in the same screening as *sav3* (the *sav1* mutant was predicted to be a weak allele of *DWF4*, which encodes a C-22 hydroxylase involved in BR biosynthesis) (Tao *et al.*, 2008). However, as a normal auxin response depends on an intact BR signal (Vert *et al.*, 2008), the transient and opposed effect of W+FR on IAA and CS levels might be part of the gas-and-brake mechanism to avoid an exaggerated auxin-induced growth after short and unsustained exposures to simulated shade. We also noticed that the list of genes significantly regulated by 1h of W+FR in *sav1* was substantially higher in *sav1* (320 in total, 258 up- and 62 downregulated) than in Col-0 (163 in total) and *sav3* (160 in total) seedlings (Supplementary Fig. S5 available at *JXB* online), suggesting that *sav1* seedlings display an increased molecular responsiveness to simulated shade, i.e. that a reduction in BR signalling might affect light signalling, at least in terms of modulation of gene expression. Therefore, in the short term, the temporary decrease in CS levels observed rapidly after exposure to W+FR might enhance the responsiveness of the seedling in terms of changes in gene expression to shade and/or the transient and antiphasic increase in auxin levels (Fig. 2 and Supplementary Fig. S5 available at *JXB* online). In the long term, however, as changes in IAA and CS levels seemed transitory (Fig. 1 and Supplementary Fig. S1 available at *JXB* online), we reasoned that they are not the main cause for enhanced hypocotyl elongation in response to simulated shade. Therefore, in addition to GA levels, seedling sensitivity to hormones might have an important effect on this trait.

Previously, a trend in the mechanisms connecting SAS and hormonal transcriptional networks was postulated: that phytochrome rapidly regulates the expression of several modulators of hormone responsiveness (Sorin *et al.*, 2009). Genotype-dependent changes in hypocotyl elongation in response to exogenously applied hormones are indicative of alterations in hormone sensitivity caused by the molecular lesion involved. It has been shown that *BIM* and *BEE* genes, whose global expression is rapidly induced after simulated shade perception (because *BIM1*, *BIM2*, *BEE1*, and *BEE2* are also *PAR* genes), have a role in SAS regulation (Cifuentes-Esquivel *et al.*, 2013). We showed here that, under W, *bee123* hypocotyls are hyposensitive to both EBL and PIC, whereas *bim123* hypocotyls are hypersensitive to BRs and hyposensitive to auxins (Fig. 6). Therefore our results support a role for BEE and BIM factors as modulators of the complex network responsible for seedling sensitivity to BRs and auxins. Similarly, the reduction in hypocotyl elongation in response to PIC (molecular and/or physiological) observed in W-grown seedlings with increased levels of PAR1 and/or PAR2 (Fig. 5) suggests a major role for these factors as modulators of the auxin sensitivity of the hypocotyls. As we performed our analyses only under W (high R:FR), the role of these different PAR factors as modulators of hypocotyl sensitivity to auxin and/or BR under simulated shade (low R:FR), although likely, would need further confirmation. Genetic analyses of other PAR factors, such as ATHB4, HAT3, and HAT2, combined with hormone applications also support their role as modulators of hormone sensitivity (Sawa *et al.*, 2002; Sorin *et al.*, 2009). Although HFR1 seems to have little or no role in affecting auxin and BR sensitivity (Fig. 4), the SAS positive regulators PIF4, PIF5, and PIF7, whose activity is inhibited by HFR1, were also reported to increase auxin sensitivity of hypocotyls (Nozue *et al.*, 2011; Hornitschek *et al.*, 2012; Li *et al.*, 2012). Therefore, a specific role for HFR1 modulating hormone sensitivity under simulated shade cannot be discarded. In summary, our results suggest that most PAR factors might be part of the molecular mechanisms employed by *Arabidopsis* to modify hormone sensitivity during the SAS.

Together, our data have important implications: they support a general mechanism by which phytochrome-mediated light perception could be rapidly transduced into global and/or local changes in hormone sensitivity. Under this postulate, W-grown seedlings display a balance of hormone modulators that, combined with the endogenous hormone levels, results in relatively short hypocotyls. After perception of plant proximity by the phytochromes, hormone levels are altered (most likely in a cell-, tissue-, and organ-specific fashion) and the level of hormone modulators is transcriptionally (i.e. *PAR* genes) or post-transcriptionally (i.e. PIFs) increased (probably with specific temporal and spatial patterns). As such hormone modulators are organized in functional modules, the transcriptional networks are reorganized at the cell, tissue, organ, and whole-plant levels (Bou-Torrent *et al.*, 2008a, *b*). As a consequence of the new balance between hormone synthesis and sensitivity, SAS responses are unleashed and hypocotyls elongate. Therefore, our results provide a molecular basis for the differential and hormone-mediated growth associated with the SAS responses after perception of plant proximity.

## Supplementary data

Supplementary data are available at JXB online.

Supplementary Fig. S1. Analysis of hormone levels in wild-type seedlings treated with simulated shade.

Supplementary Fig. S2. Merging of microarray data from shade-regulated and BL- and GA-regulated genes.

Supplementary Fig. S3. Cartoon depicting the different truncated HFR1 derivatives overexpressed in transgenic plants.

Supplementary Fig. S4. Effect of PAR1 and PAR2 on BR-induced gene expression.

Supplementary Fig. S5. Venn diagrams illustrating the subgroup of genes in wild-type, *sav1*, and *sav3* mutant seedlings in response to 1h of simulated shade.

Supplementary Table S1. ANOVA table for experiments shown in Figs 2B, D.

Supplementary Table S2. List of regulated genes identified in response to 1h of W+FR in wild-type, *sav1*, and *sav3* mutant seedlings.

Supplementary Table S3. List of shade- regulated and IAA-, BL-, and GA-regulated genes.

Supplementary Table S4. ANOVA table for the experiment shown in Fig. 4.

Supplementary Table S5. ANOVA table for the experiments shown in Fig. 5.

Supplementary Table S6. ANOVA table for the experiments shown in Fig. 6.

Supplementary Table S7. Changes in the expression of genes reported as involved in some aspects of BR (A) or GA (B) metabolism or inactivation in response to short (1h) or long (24h) treatments with simulated shade.

Supplementary Data

## Abbreviations:

bHLH: basic helix–loop–helix
BR: brassinosteroid
CS: castasterone
EBL: epibrassinolide
FR: far-red light
GA: gibberellin
GA_3_: gibberellin A_3_
GFP: green fluorescent protein
GUS: β-glucuronidase
IAA: indole-3-acetic acid
PAC: paclobutrazol
PCZ: propiconazol
PIC: picloram
R: red light
SAS: *s*hade *a*voidance syndrome
SE: standard error
W: white light.
