# Supplementary material for: Plant proximity perception dynamically modulates hormone levels and sensitivity in Arabidopsis
Source: J Exp Bot. 2014 Mar 8;65(11):2937–47. doi: 10.1093/jxb/eru083 (PMC4056540; doi:10.1093/jxb/eru083)
Supplement: Supplementary Data [file supp_65_11_2937__index.html]

Plant proximity perception dynamically modulates hormone levels and sensitivity in Arabidopsis — Plant proximity perception dynamically modulates hormone levels and sensitivity in Arabidopsis — Supplementary Data 

# Plant proximity perception dynamically modulates hormone levels and sensitivity in *Arabidopsis*

## Supplementary Data

Data files

**Files in this Data Supplement:**

- Supplementary Data - Supplementary Data
