# Supplementary material for: Plant proximity perception dynamically modulates hormone levels and sensitivity in Arabidopsis
Source: J Exp Bot. 2014 Mar 8;65(11):2937–47. doi: 10.1093/jxb/eru083 (PMC4056540; doi:10.1093/jxb/eru083)
Supplement: Supplementary Data [file supp_eru083_jexbot108530_file001.pdf]

SUPPLEMENTAL INFORMATION

Plant proximity perception dynamically modulates hormone levels and sensitivity in Arabidopsis

Jordi Bou-Torrent, Anahit Galstyan, Marçal Gallemí, Nicolás Cifuentes-Esquivel, María José Molina-Contreras, Mercè Salla-Martret, Yusuke Jikumaru, Shinjiro Yamaguchi, Yuji Kamiya and Jaime F Martínez-García

SUPPLEMENTAL DATA

Figure S1. Analysis of hormone levels in wild-type seedlings treated with simulated shade. Seedlings were germinated and grown for 5 days under W and then either kept in W or transferred to W+FR for 4 h. Data represent the mean ± SE of three experiments; FW, fresh weight. Different letters denote significant differences ($P < 0.01$ for IAA levels; $P<0.05$ for CS levels; and $P<0.1$ for GA_4 levels) among means.

Figure S2. Merging of microarray data from shade-regulated and BL- and GA-regulated genes. **(A)** Venn diagrams illustrating the overlap between the class SD (left), class SU (right), class of BL up- (top) and down-regulated (bottom) genes. The comparison between the different groups of genes defines the class S+B, listed in Table S3. **(B)** Venn diagrams illustrating the overlap between the class SD (left), class SU (right), class of GA up- (top) and down-regulated (bottom) genes. The comparison between the different groups of genes defines the class S+G, listed in Table S3.

Figure S3. Cartoon depicting the different truncated HFR1 derivatives overexpressed in transgenic plants. Transgenes were expressed under the control of the 35S promoter (P_{35S}) and fused to the GFP reporter protein. The name of the corresponding transgenic plants and their response to W+FR (Galstyan *et al.*, 2011) is summarized in the right.

Figure S4. Effect of PAR1 and PAR2 on BR-induced gene expression. **(A)** Seven-day-old W-grown seedlings were treated with 1 μM EBL for the indicated times (in hours). **(B)** RNA blot analysis of *SAUR15*, *SAUR68* and *At5g45670* expression in wt and transgenic seedlings overexpressing PAR1-GG (line 13) and PAR2-G (line 03). Plant material for RNA extraction was harvested at the time points indicated. *25S* rRNA levels are shown as a loading control of the corresponding (upper) membranes.

Figure S5. Venn diagrams illustrating the subgroup of shade-responding genes in wild-type, *sav1* and *sav3* mutant seedlings. Merging of microarray data from rapidly down- (left) and up-regulated (right) group of genes in wild-type (Col-0), *sav1* and *sav3* mutant seedlings in response to 1h of W+FR (Tao *et al.*, 2008). The total number of genes in each group, indicated in parenthesis, is listed in Table S2. The numbers of genes in each sector is indicated.

Table S1. ANOVA table for experiments shown in Figure 2.

Table S2. List of regulated genes identified in response to 1h of W+FR in, wild-type, *sav1* and *sav3* mutant seedlings.

Table S3. List of shade- regulated and IAA-, BL- and GA-regulated genes.

Table S4. ANOVA table for experiments shown in Figure 4.

Table S5. ANOVA table for experiments shown in Figure 5.

Table S6. ANOVA table for experiments shown in Figure 6.

Table S7. Changes in the expression of genes reported as involved in some aspects of BR **(A)** or GA **(B)** metabolism or inactivation in response to short (1 h) or long treatments (24 h) with simulated shade.

SUPPLEMENTAL EXPERIMENTAL PROCEDURES

CS levels quantification

At the head of extraction, approximately 500 pg/g fresh weight of $[^2H]_4$-CS was added as an internal standard. Neutral extracts containing BRs were prepared by sequential extraction with hydrophobic, cation-exchange and anion-exchange cartridges, as described (Yoshimoto *et al.*, 2009). Extracts were dried, suspended in 1 ml of $CHCl_3$ and loaded onto normal phase extraction cartridge (Waters, SepPak Silica). BRs were eluted with 2 ml of $CHCl_3$:MeOH (9:1, v/v) after washing with 1 ml of $CHCl_3$. Extracts were dried and dissolved into 100 µl of 50% MeOH and subjected to HPLC equipped with ODS column (SHISEIDO, CAPCELL PAK C18, 4.6x250 mm). Separation was performed using a gradient of increasing acetonitrile to water (flow rate of 1 ml/min). 10% acetonitrile was eluted over 10 min and increased to 40% over 11 min. Then concentration of acetonitrile was increased to 60% over 30 min. After washing with 90% acetonitrile over 40 min, the initial condition was restored and allowed to equilibrate over 50 min. Eluents were collected for 1 tube/min. After drying up the solvent, fractions 23rd to 30th were

analyzed with liquid chromatography-electrospray ionization-tandem mass spectrometry (LC-ESI-MS/MS) (Agilent, 1200-6410). LC conditions and parameters for LC-ESI-MS/MS analysis are shown in the Table below. Ratio was calculated comparing areas of endogenous to internal standard. Concentration of endogenous CS was calculated with the average of three specific productions.

LC conditions of LC-ESI-MS/MS analysis

Solvent A	Solvent B	Composition of solvent B
Water containing 0.01% pyridine	Acetonitrile containing 0.01% pyridine	3 to 63% over 18 min

MS conditions of LC-ESI-MS/MS analysis

	Retention time (min)	Charge	MS/MS transition (*m/z*)	Collision energy (V)	Fragmentor (V)
**Castasterone (CS)**	15.5	–	463/129<br>463/411<br>463/427	22	190
**$[^2H]_4$-Castasterone ($[^2H]_4$-CS)**	15.4	–	467/129<br>467/415<br>467/431	22	190

RNA blot analysis

Total RNA was isolated from seedlings, electrophoresed (10 µg) and blotted as described elsewhere (Roig-Villanova *et al.*, 2006). Hybridization probes for *SAUR15*, *SAUR68* and *25S* rRNA were prepared as described (Sorin *et al.*, 2009). Probe for *At5g45670* was made by amplifying Col-0 genomic DNA with specific primers BO42 (5'-TAG-AAA-AAG-ATG-GCG-AGA-ATG-AGT-3') and BO43 (5'-GAT-

CTT-TCT-CTC-TTT-AGA-GAG-ATG-C-3'). The resulting PCR product was subcloned into pGEMT-easy (Promega, www.promega.com) to give pJB31. Inserts were sequenced for identity confirmation.

Probes for DNA inserts, isolated by restriction digestion or by PCR using specific primers, were radioactively labeled with [α^{32}P]dCTP by using a random primed DNA-labeling kit (www.roche-applied-science.com) or by PCR using one specific primer to labels only the antisense strand. Labeled DNA was purified through Sephadex G-50 columns (GE Helthcare, www1.gelifesciences.com). Hybridization, washes, exposure and quantification of radioactive signals were carried out as described (Roig-Villanova *et al.*, 2006). Images were visualized by using a molecular Imager FX (Bio-Rad, www.bio-rad.com) Expression levels were normalized with the *25S* rRNA signal.

SUPPLEMENTAL REFERENCES

Galstyan A, Cifuentes-Esquivel N, Bou-Torrent J, Martinez-Garcia JF. 2011. The shade avoidance syndrome in Arabidopsis: a fundamental role for atypical basic helix-loop-helix proteins as transcriptional cofactors. *The Plant Journal* **66**, 258-267.

Roig-Villanova I, Bou J, Sorin C, Devlin PF, Martinez-Garcia JF. 2006. Identification of primary target genes of phytochrome signaling. Early transcriptional control during shade avoidance responses in Arabidopsis. *Plant Physiology* **141**, 85-96.

Sorin C, Salla-Martret M, Bou-Torrent J, Roig-Villanova I, Martinez-Garcia JF. 2009. ATHB4, a regulator of shade avoidance, modulates hormone response in Arabidopsis seedlings. *The Plant Journal* **59**, 266-277.

Tao Y, Ferrer JL, Ljung K*, et al.* 2008. Rapid synthesis of auxin via a new tryptophan-dependent pathway is required for shade avoidance in plants. *Cell* **133**, 164-176.

Yoshimoto K, Jikumaru Y, Kamiya Y, Kusano M, Consonni C, Panstruga R, Ohsumi Y, Shirasu K. 2009. Autophagy negatively regulates cell death by controlling NPR1-dependent salicylic acid signaling during senescence and the innate immune response in Arabidopsis. *The Plant Cell* **21**, 2914-2927.

Figure S1

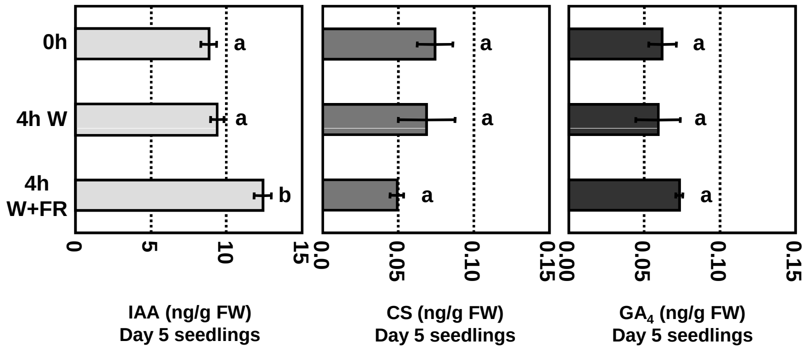

Figure S1. Analysis of hormone levels in wild-type seedlings treated with simulated shade. Seedlings were germinated and grown for 5 days under W and then either kept in W or transferred to W+FR for 4 h. Data represent the mean ± SE of three experiments; FW, fresh weight. Different letters denote significant differences ($P < 0.01$ for IAA levels; $P<0.05$ for CS levels; and $P<0.1$ for GA_4 levels) among means.

Figure S2

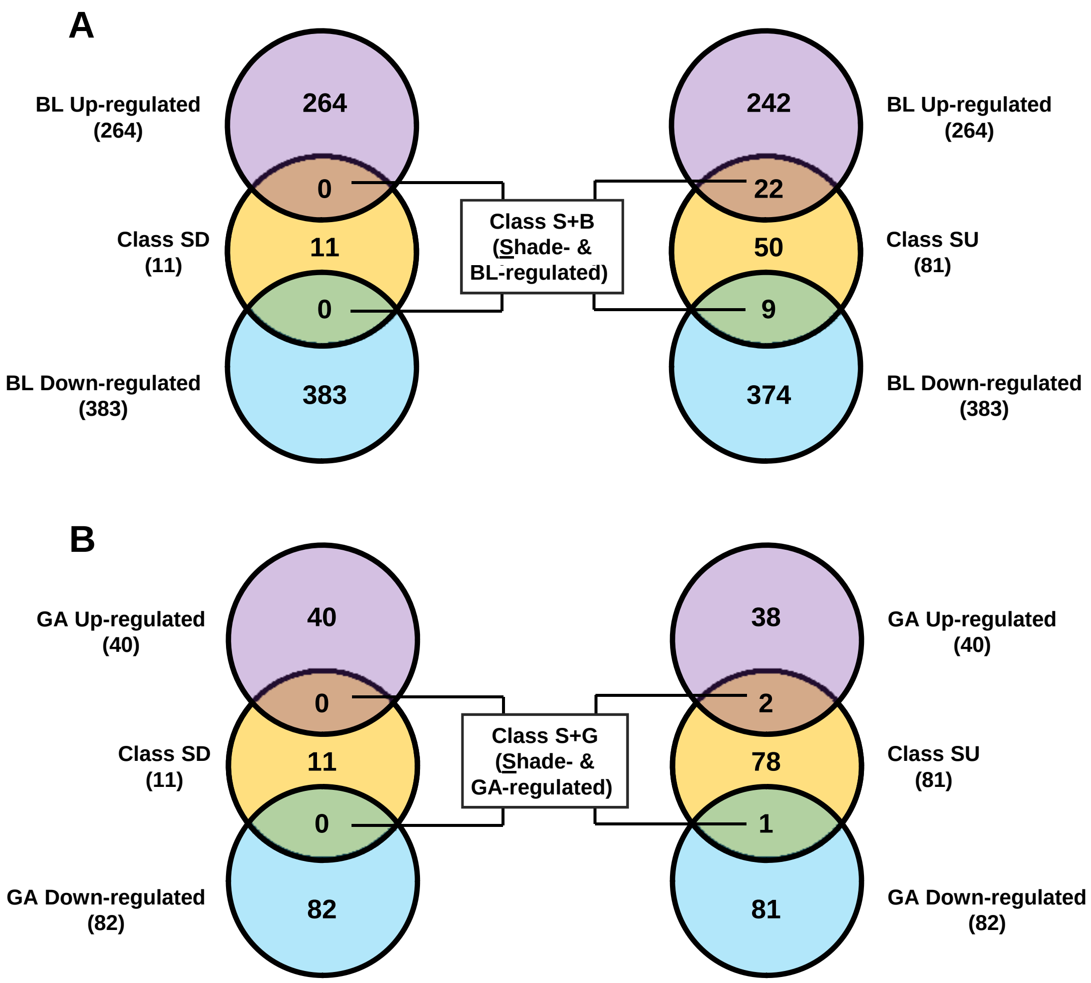

Figure S2. Merging of microarray data from shade-regulated and BL- and GA-regulated genes. **(A)** Venn diagrams illustrating the overlap between the class SD (left), class SU (right), class of BL up- (top) and down-regulated (bottom) genes. The comparison between the different groups of genes defines the class S+B, listed in Table S3. **(B)** Venn diagrams illustrating the overlap between the class SD (left), class SU (right), class of GA up- (top) and down-regulated (bottom) genes. The comparison between the different groups of genes defines the class S+G, listed in Table S3.

Figure S3

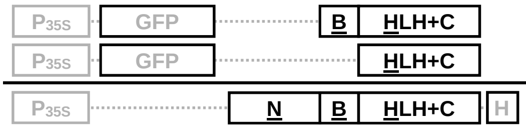

		Response to W+FR	
	name	HYP elongation	*PIL1* expression
	G-BH	Very reduced	Very reduced
	G-H	Abolished	Abolished
	HFR1-HA	Slightly reduced	N.D.

Figure S3. Cartoon depicting the different truncated HFR1 derivatives overexpressed in transgenic plants. Transgenes were expressed under the control of the 35S promoter (P35S) and fused to the GFP reporter protein. The name of the corresponding transgenic plants and their response to W+FR (Galstyan et al., 2011) is summarized in the right.

Figure S4

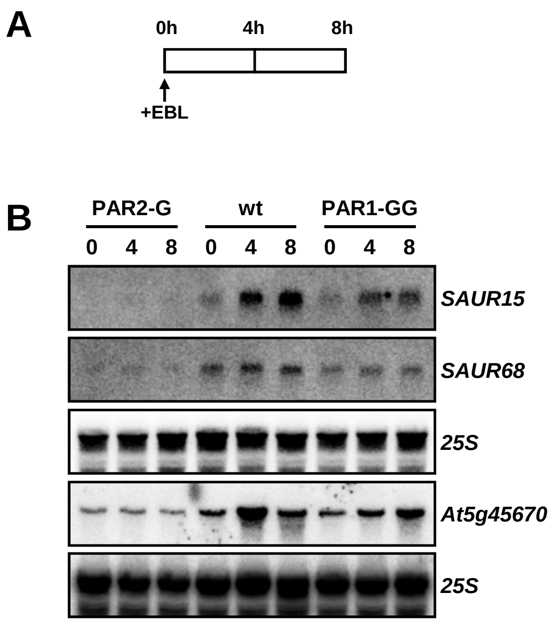

Figure S4. Effect of PAR1 and PAR2 on BR-induced gene expression. **(A)** Seven-day-old W-grown seedlings were treated with 1 µM EBL for the indicated times (in hours). **(B)** RNA blot analysis of *SAUR15*, *SAUR68* and *At5g45670* expression in wt and transgenic seedlings overexpressing PAR1-GG (line 13) and PAR2-G (line 03). Plant material for RNA extraction was harvested at the time points indicated. *25S* rRNA levels are shown as a loading control of the corresponding (upper) membranes.

Figure S5

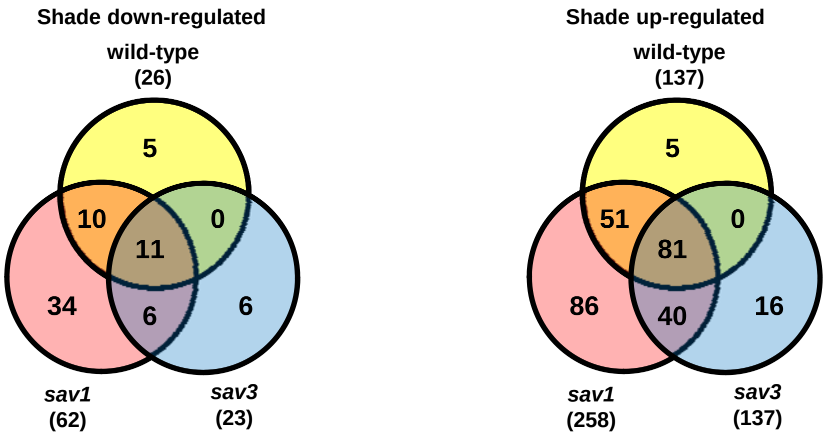

Figure S5. Venn diagrams illustrating the subgroup of shade-responding genes in wild-type, *sav1* and *sav3* mutant seedlings. Merging of microarray data from rapidly down- (left) and up-regulated (right) group of genes in wild-type (Col-0), *sav1* and *sav3* mutant seedlings in response to 1h of W+FR (Tao et al., 2008). The total number of genes in each group, indicated in parenthesis, is listed in Table S2. The numbers of genes in each sector is indicated.

Table S1. ANOVA table for experiments shown in Figures 2B and 2D. DF, Degrees of Freedom; SS, Sum of Squares; MS; Mean Square.

HYP elongation in W vs. W+FR (Figure 2B)

Source of variation	DF	SS	MS	F-value	p-value
**Simulated shade**	1	225.8	225.8	1162.91	<0.0001
**PIC**	2	27.21	13.60	70.06	<0.0001
**Interaction**	2	64.28	32.14	165.52	**<0.0001**
**Residual (error)**	174	33.79	0.1942	-	-

HYP elongation in W vs. W+FR (Figure 2B)

Source of variation	DF	SS	MS	F-value	p-value
**Simulated shade**	1	182.2	182.2	604.86	<0.0001
**EBL**	2	69.0	34.5	114.52	<0.0001
**Interaction**	2	70.74	35.37	117.39	**<0.0001**
**Residual (error)**	139	41.88	0.3013	-	-

HYP elongation in W vs. W+FR (Figure 2B)

Source of variation	DF	SS	MS	F-value	p-value
**Simulated shade**	1	652.6	652.6	2106.90	<0.0001
**GA3**	2	63.35	31.67	102.25	<0.0001
**Interaction**	2	2.671	1.336	4.31	**0.0155**
**Residual (error)**	120	37.17	0.3098	-	-

HYP elongation in W vs. W+FR (Figure 2D)

Source of variation	DF	SS	MS	F-value	p-value
**Simulated shade**	1	11.79	11.79	45.99	<0.0001
**EBL**	4	370.2	92.54	361.05	<0.0001
**Interaction**	4	14.66	3.665	14.30	**<0.0001**
**Residual (error)**	263	67.41	0.2563	-	-

HYP elongation in W vs. W+FR (Figure 2D)

Source of variation	DF	SS	MS	F-value	p-value
**Simulated shade**	1	42.48	42.48	1003.52	<0.0001
**GA3**	2	59.31	29.65	700.46	<0.0001
**Interaction**	2	19.06	9.530	225.10	**<0.0001**
**Residual (error)**	111	4.699	0.04234	-	-

Table S2. List of regulated genes identified in response to 1h of W+FR in, wild-type, *sav1* and *sav3* mutant seedlings.

Shade-regulated in Col-0 seedlings

Down-regulated genes identified in response to 1h of W+FR in wild-type (Col-0) seedlings. "Down-regulated" indicates genes that have significantly (BH<0.05) lower signal (Fold change < 1.5) after 1h of W+FR compared to their levels at time 0 (inmediately before starting the simulated shade treatment).

Sequence ID	Accession #	Sequence Name(s)	Sequence Description	Log (Error)	Log (Ratio)	Ratio	Fold Change	BH
950147	At4g02410	255502_at	contains similarity to a protein kinase domain (Pfam: pkinase.hmm, score: 166.20) and to legume lectins beta domain (Pfam: lectin_legB.hmm, score: 139.32)	0.03521	-0.17845	0.66306	-1.50817	6.41E-05
952964	At3g02910	258609_at	unknown protein ;supported by full-length cDNA: Ceres:269248.	0.04363	-0.18302	0.65612	-1.52412	0.003153
954060	At1g77760	NIA1	nitrate reductase 1 (NR1) identical to nitrate reductase 1 (NR1) GB:P11832 [Arabidopsis thaliana]; supported by cDNA: gi_15983498_gb_AF424624.1_AF424624	0.02868	-0.18347	0.65544	-1.52569	4.19E-08
956953	At1g15260	262598_at	unknown protein EST gb|N65467 comes from this gene;supported by full-length cDNA: Ceres:30239.	0.02234	-0.18414	0.65442	-1.52806	8.76E-14
945089	At5g10030	250463_at	transcription factor OBF4	0.03715	-0.18464	0.65367	-1.52983	1.03E-04
958564	At1g54820	264240_at	protein kinase, putative Simisimilar to protein kinase 2 GI:7573598 from [Populus nigra]	0.04203	-0.18738	0.64957	-1.53949	1.05E-03
957334	At1g75710	262969_at	unknown protein	0.04266	-0.18778	0.64896	-1.54093	0.001164
955903	At1g01140	261581_at	serine/threonine kinase, putative similar to serine/threonine kinase GB:CAA73067.1 GI:2632252 from [Sorghum bicolor]; supported by cDNA: gi_14423523_gb_AF386999.1_AF386999	0.0447	-0.19689	0.63549	-1.57359	0.001164
961409	At2g37710	267165_at	putative receptor-like protein kinase same as GB:X95909 (polymorphism exists at a GA repeat. We found 6 copies in our sequence whereas only 5 copies exist in GB:X95909)	0.03641	-0.2027	0.62705	-1.59478	5.07E-06
947271	At3g45210	252619_at	putative protein several hypothetical proteins - Arabidopsis thaliana; supported by cDNA: gi_15724293_gb_AF412087.1_AF412087	0.03336	-0.21022	0.61629	-1.62263	7.37E-08
945930	At3g61890	ATHB-12	homeobox-leucine zipper protein ATHB-12 ;supported by full-length cDNA: Ceres:32615.	0.05186	-0.21882	0.60419	-1.6551	0.002152
948984	At4g21990	APR3	PRH26 protein ;supported by full-length cDNA: Ceres:36866.	0.05452	-0.22652	0.59358	-1.68468	0.003153
948764	At4g24780	254119_at	putative pectate lyase pectate lyase, Musa acuminata, PATX:E209876;supported by full-length cDNA: Ceres:36681.	0.0408	-0.22874	0.59056	-1.69331	4.29E-06
949219	At4g19380	254572_at	putative protein predicted protein MTCY20G9, Mycobacterium tuberculosis	0.04846	-0.23808	0.57798	-1.73015	1.36E-04

941671	At5g67280	246986_at	receptor-like protein kinase ;supported by full-length cDNA: Ceres:110712.	0.03437	-0.24579	0.56782	-1.76114	3.04E-10
960280	At2g15050	265894_at	putative lipid transfer protein	0.02684	-0.24688	0.56639	-1.76556	2.97E-17
947259	At3g44990	XTR8	xyloglucan endo-transglycosylase ; supported by cDNA: gi_15810248_gb_AY056163.1_	0.03971	-0.25758	0.55262	-1.80957	2.46E-08
956567	At1g74940	262170_at	hypothetical protein predicted by genemark.hmm;supported by full-length cDNA: Ceres:24864.	0.04359	-0.26698	0.54078	-1.84917	2.21E-07
950790	At1g51805	256168_at	receptor protein kinase, putative contains Pfam profiles: PF00069: Eukaryotic protein kinase domain, multiple PF00560: Leucine Rich Repeat	0.03422	-0.27325	0.53302	-1.87609	6.61E-13
947096	At3g47420	252414_at	putative protein sn-glycerol-3-phosphate permease - Haemophilus influenzae,PID:g3603157; supported by cDNA: gi_13430515_gb_AF360170.1_AF360170	0.0593	-0.27475	0.53119	-1.88257	4.98E-04
953273	At3g10525	258919_at	Expressed protein ; supported by full-length cDNA: Ceres: 2153.	0.06237	-0.27743	0.52792	-1.89422	1.08E-03
954461	At1g78170	260081_at	unknown protein	0.03821	-0.29174	0.51081	-1.95768	9.12E-12
959272	At2g17300	264909_at	unknown protein	0.06929	-0.30676	0.49345	-2.02654	1.16E-03
943916	At5g41410	249309_at	homeotic protein BEL1 homolog ; supported by cDNA: gi_15146192_gb_AY049237.1_	0.07164	-0.3096	0.49023	-2.03987	0.002152
954728	At1g70560	TAA1	putative alliinase similar to aliinase precursor GB:AAD26853 [Allium cepa]	0.05473	-0.34887	0.44785	-2.23291	4.77E-08
947308	At3g44450	252661_at	putative protein	0.08904	-0.44725	0.35707	-2.8006	7.90E-05

Up-regulated genes identified in response to 1h of W+FR in wild-type (Col-0) seedlings. "Up-regulated" indicates genes that have significantly (BH<0.05) higher signal (Fold change > 1.5) after 1h of W+FR compared to their levels at time 0 (inmediately before starting the simulated shade treatment).

Sequence ID	Accession #	Sequence Name(s)	Sequence Description	Log (Error)	Log (Ratio)	Ratio	Fold Change	BH
947650	At4g37770	253066_at	1-aminocyclopropane-1-carboxylate synthase - like protein 1-aminocyclopropane-1-carboxylate synthase,Arabidopsis thaliana, S71174; supported by cDNA: gi_12247996_gb_AF334712.1_AF334712	0.20231	1.3997	25.10124	25.10124	1.51E-09
958701	At1g04180	YUC9	YUCCA9 - putative dimethylaniline monooxygenase	0.14058	1.15111	14.16142	14.16142	1.34E-13
956171	At1g15580	IAA5	auxin-induced protein IAA5, putative similar to auxin-induced protein IAA5 GI:972913 from [Arabidopsis thaliana]	0.06258	1.12505	13.3367	13.3367	0
963796	At4g16780	ATHB2	DNA-binding homeotic protein Athb-2 ; supported by cDNA: gi_166751_gb_M90394.1_ATHHOMEOA	0.07788	1.09344	12.4005	12.4005	1.74E-41
958331	At2g42870	PAR1	unknown protein ;supported by full-length cDNA: Ceres:102453.	0.15188	1.06334	11.57015	11.57015	8.52E-10

948061	At4g32280	253423_at	Expressed protein ; supported by cDNA: gi_14190492_gb_AF380646.1_AF380646	0.14771	0.942	8.74978	8.74978	4.72E-08
945281	At5g07010	250662_at	steroid sulfotransferase-like protein ;supported by full-length cDNA: Ceres:124067.	0.13376	0.90095	7.96076	7.96076	5.02E-09
945801	At3g63440	251178_at	cytokinin oxidase -like protein cytokinin oxidase, Zea mays, EMBL:ZMY18377	0.09819	0.89729	7.89395	7.89395	5.00E-17
953710	At3g03830	259332_at	putative auxin-induced protein similar to SAUR GB:BAA25434 [Raphanus sativus]	0.06429	0.87031	7.41833	7.41833	1.77E-38
952778	At3g15540	IAA19	early auxin-induced protein, IAA19 identical to IAA19 GB:AAB84356 from [Arabidopsis thaliana]; supported by full-length cDNA: Ceres: 94231.	0.04467	0.85056	7.08858	7.08858	0
954520	At1g52830	IAA6	putative IAA6 protein similar to IAA6 protein GB:S5849 from [Arabidopsis thaliana];supported by full-length cDNA: Ceres:147711.	0.04542	0.84959	7.07277	7.07277	0
955504	At1g75450	261109_at	cytokinin oxidase, putative similar to GB:CAA77151 from [Zea mays] (Plant J. 17 (6), 615-626 (1999))	0.15817	0.83952	6.91068	6.91068	1.99E-05
948743	At4g25420	254065_at	gibberellin 20-oxidase - Arabidopsis thaliana	0.13703	0.82959	6.75438	6.75438	3.35E-07
948437	At4g28720	YUC8	YUCCA8 - putative protein dimethylaniline monooxygenase (N-oxide-forming), Sus scrofa domestica, PIR1:A33768	0.08538	0.82652	6.7069	6.7069	3.48E-19
961190	At2g22810	ACS4	1-aminocyclopropane-1-carboxylate synthase (ACS4) identical to GB:U23481; supported by cDNA: gi_12083215_gb_AF332404.1_AF332404	0.14501	0.82347	6.65995	6.65995	2.85E-06
953791	At1g02340	HFR1	unknown protein contains similarity to phytochrome interacting factor 3 GI:3929586 from [Arabidopsis thaliana]; supported by cDNA: gi_11870114_gb_AF324245.1_AF324245	0.08816	0.80059	6.3181	6.3181	7.87E-17
961174	At2g44910	ATHB4	homeodomain transcription factor (ATHB-4)	0.17104	0.79997	6.3092	6.3092	4.10E-04
960166	At2g18010	265806_at	putative auxin-regulated protein	0.05966	0.79837	6.28599	6.28599	1.36E-37
954228	At1g69570	259834_at	H-protein promoter binding factor-2b nearly identical to H-protein promoter binding factor-2b (Arabidopsis thaliana) GI:3386548; supported by cDNA: gi_3386547_gb_AF079504.1_AF079504	0.10033	0.79265	6.20367	6.20367	1.22E-12
963299	At1g35140	245757_at	phosphate-induced (phi-1) protein, putative similar to phi-1 GB:BAA33810 GI:3759184 from [Nicotiana tabacum];supported by full-length cDNA: Ceres:118937.	0.15839	0.78511	6.09694	6.09694	1.09E-04
942556	At5g57560	TCH4	TCH4 protein (gb|AAA92363.1) ; supported by cDNA: gi_14194112_gb_AF367262.1_AF367262	0.08867	0.775	5.95659	5.95659	1.47E-15
964006	At2g23170	245076_at	unknown protein	0.0869	0.76882	5.87246	5.87246	6.18E-16
943399	At5g47370	248801_at	homeobox-leucine zipper protein-like ; supported by cDNA: gi_15450446_gb_AY052324.1_	0.06073	0.76016	5.75653	5.75653	8.08E-33
944690	At5g18060	250012_x_at	auxin-induced protein-like	0.06223	0.75475	5.68524	5.68524	8.44E-31

958225	At2g37030	263890_at	putative auxin-induced protein	0.10122	0.74558	5.56644	5.56644	6.80E-11
942972	At5g52900	248282_at	unknown protein ;supported by full-length cDNA: Ceres:148254.	0.04303	0.72489	5.30755	5.30755	0
948574	At4g27280	253915_at	putative protein centrin, Marsilea vestita;supported by full-length cDNA: Ceres:13072.	0.05608	0.71243	5.15744	5.15744	8.52E-34
947778	At4g36110	253103_at	putative auxin-induced protein high similarity to auxin-induced protein 15A, soybean, PIR2:JQ1096; supported by cDNA: gi_13194817_gb_AF349524.1_AF349524	0.14161	0.71184	5.15038	5.15038	7.80E-05
943709	At5g43890	YUC5	YUCCA5 - dimethylaniline monooxygenase-like	0.13663	0.70692	5.09234	5.09234	3.84E-05
945609	At5g02760	PP2C	protein phosphatase - like protein protein phosphatase 2C homolog, Mesembryanthemum crystallinum, EMBL:AF097667	0.05733	0.69634	4.96978	4.96978	7.18E-31
953709	At3g03840	259331_at	putative auxin-induced protein similar to SAUR GB:BAA25434 [Raphanus sativus]	0.03478	0.67238	4.70304	4.70304	0
949810	At4g08040	255177_at	strong similarity to 1-aminocyclopropane-1-carboxylic acid synthases	0.15087	0.66557	4.62993	4.62993	0.001164
942112	At5g62280	247474_at	putative protein predicted proteins, Arabidopsis thaliana	0.0901	0.64443	4.40992	4.40992	3.03E-10
943739	At5g44260	249065_at	putative protein similar to unknown protein (gb AAD10689.1); supported by cDNA: gi_14334449_gb_AY034916.1_	0.1447	0.64328	4.39826	4.39826	1.09E-03
963695	At4g14560	IAA1	auxin-responsive protein IAA1 ;supported by full-length cDNA: Ceres:33860.	0.08809	0.63659	4.33103	4.33103	1.82E-10
951396	At3g29370	256743_at	Expressed protein ; supported by full-length cDNA: Ceres: 22461.	0.09035	0.63487	4.31386	4.31386	7.22E-10
943073	At5g51810	248371_at	gibberellin 20-oxidase (emb|CAA58294.1)	0.12541	0.63378	4.30306	4.30306	6.87E-05
949357	At4g13790	254685_at	SAUR-AC - like protein (small auxin up RNA) SAUR-AC1, Arabidopsis thaliana, S70188;supported by full-length cDNA: Ceres:8965.	0.14976	0.61787	4.14829	4.14829	0.004055
946880	At3g50340	252204_at	putative protein predicted protein, Arabidopsis thaliana	0.03754	0.60316	4.01011	4.01011	0
941705	At5g66590	247074_at	putative protein contains similarity to pathogenesis-related protein;supported by full-length cDNA: Ceres:2152.	0.09149	0.59946	3.97617	3.97617	1.64E-08
944978	At5g12050	250327_at	putative serine rich protein predicted proteins, Arabidopsis thaliana;supported by full-length cDNA: Ceres:36958.	0.10447	0.59404	3.92684	3.92684	2.77E-06
962983	At5g25190	246932_at	ethylene-responsive element - like protein ethylene-responsive element binding protein homolog, Stylosanthes hamata, EMBL:U91857; supported by cDNA: gi_15010715_gb_AY045659.1_	0.08942	0.58561	3.85129	3.85129	1.65E-08
952200	At3g28420	257900_at	hypothetical protein predicted by genemark.hmm	0.0865	0.57869	3.79041	3.79041	6.78E-09
963364	At5g04190	245696_at	phytochrome kinase substrate 1 - like protein	0.1088	0.56596	3.68094	3.68094	3.33E-05
960217	At2g42380	265877_at	unknown protein ; supported by cDNA: gi_15100052_gb_AF401299.1_AF401299	0.07738	0.56486	3.67163	3.67163	1.09E-10
947838	At4g34770	253207_at	putative protein small auxin up-regulated RNA, Malus domestica, gb:Z93766	0.07576	0.54693	3.52317	3.52317	1.89E-10
943130	At5g51190	248448_at	putative protein contains similarity to ethylene responsive element binding factor;supported by full-length cDNA: Ceres:2347.	0.11557	0.53328	3.41412	3.41412	5.43E-04

943913	At5g41400	249306_at	RING zinc finger protein-like ;supported by full-length cDNA: Ceres:207148.	0.08209	0.52616	3.35864	3.35864	3.92E-08
943496	At5g46240	248888_at	potassium channel protein KAT1 (pir||S32816) ; supported by cDNA: gi_166773_gb_M86990.1_ATHKAT1	0.07682	0.52378	3.34024	3.34024	2.91E-09
964362	At1g29440	257506_at	auxin-induced protein, putative similar to SP:P33083 from [Glycine max]	0.03231	0.52327	3.33631	3.33631	0
956093	At1g18400	BEE1	helix-loop-helix protein homolog, putative similar to helix-loop-helix protein homolog GB:BAA87957 GI:6520231 from [Arabidopsis thaliana]	0.08639	0.50873	3.22648	3.22648	8.79E-07
954133	At1g29460	259787_at	auxin-induced protein, putative similar to SP:P33083 from [Glycine max];supported by full-length cDNA: Ceres:147801.	0.05454	0.50872	3.22644	3.22644	9.12E-18
945276	At5g07000	250657_at	steroid sulfotransferase-like protein	0.03815	0.50692	3.21303	3.21303	4.40E-37
950307	At3g29575	255723_at	hypothetical protein	0.11088	0.49484	3.12494	3.12494	1.04E-03
952152	At3g23030	IAA2	auxin-inducible gene (IAA2) identical to auxin-inducible gene (IAA2) GB:AF027157 [Arabidopsis thaliana] (Plant Physiol. 115, 1730 (1997))	0.04626	0.49465	3.12357	3.12357	1.14E-23
957573	At1g10550	263207_at	putative endoxyloglucan transferase similar to xyloglucan endotransglycosylase-related protein XTR4 (pir|IS71223);supported by full-length cDNA: Ceres:27813.	0.12339	0.49146	3.10067	3.10067	0.006681
961807	At2g26710	267614_at	putative cytochrome P450	0.05387	0.47311	2.9724	2.9724	1.04E-15
950221	At4g01250	255568_at	putative DNA-binding protein ; supported by cDNA: gi_15028172_gb_AY045909.1_	0.06398	0.46632	2.92628	2.92628	1.17E-10
946099	At3g59900	251436_at	putative protein hypothetical protein At2g44080 - Arabidopsis thaliana, EMBL:AC004005	0.07039	0.46129	2.89261	2.89261	1.64E-08
955287	At1g02660	260915_at	hypothetical protein similar to hypothetical protein GB:CAB83109 GI:7362739 from [Arabidopsis thaliana]; supported by cDNA: gi_15081706_gb_AY048246.1_	0.04862	0.46019	2.8853	2.8853	2.68E-18
955780	At1g21050	261456_at	hypothetical protein predicted by genemark.hmm;supported by full-length cDNA: Ceres:3200.	0.05206	0.45312	2.83868	2.83868	1.97E-15
961160	At2g30040	266832_at	putative protein kinase contains a protein kinase domain profile (PDOC00100)	0.05333	0.45237	2.83379	2.83379	1.26E-14
954119	At1g29500	259773_at	auxin-induced protein, putative similar to SP:P33083 from [Glycine max]	0.03211	0.4521	2.83207	2.83207	1.28E-41
952407	At3g19380	258021_at	hypothetical protein predicted genemark;supported by full-length cDNA: Ceres:255040.	0.0529	0.44908	2.81242	2.81242	1.22E-14
954130	At1g29450	259784_at	auxin-induced protein, putative similar to SP:P33083 from [Glycine max];supported by full-length cDNA: Ceres:29931.	0.04847	0.42626	2.66845	2.66845	9.73E-16
947626	At4g38400	252997_at	putative pollen allergen pollen allergen - Pinus radiata, PID:g2935527; supported by cDNA: gi_14190354_gb_AF378855.1_AF378855	0.05061	0.41779	2.61692	2.61692	8.06E-14

945190	At5g08130	BIM1	basic helix-loop-helix (bHLH) family protein involved in brassinosteroid signaling.	0.09024	0.4148	2.59899	2.59899	5.87E-04
960446	At2g18790	PHYB	phytochrome B Identical to GB:X17342	0.04899	0.41174	2.5807	2.5807	2.35E-14
963761	At4g16515	245336_at	Expressed protein ; supported by full-length cDNA: Ceres: 6580.	0.04634	0.39192	2.46557	2.46557	1.51E-14
949396	At4g13260	YUC2	putative protein dimethylaniline monooxygenase (N-oxide-forming) (EC1.14.13.8) -Oryctolagus cuniculus,PID:g164989	0.05935	0.3908	2.45923	2.45923	1.34E-08
954136	At1g29430	259790_s_at	auxin-induced protein, putative similar to SP:P33083 from [Glycine max];supported by full-length cDNA: Ceres:4119.	0.03145	0.38811	2.44402	2.44402	7.07E-32
955188	At1g21910	260856_at	TINY-like protein similar to TINY GB:CAA64359 GI:1246403 from [Arabidopsis thaliana];supported by full-length cDNA: Ceres:19721.	0.088	0.38121	2.40555	2.40555	0.001164
947577	At4g38850	252970_at	small auxin up RNA (SAUR-AC1) ;supported by full-length cDNA: Ceres:14973.	0.04685	0.378	2.38782	2.38782	3.45E-13
955971	At1g33055	261567_at	Expressed protein ; supported by cDNA: gi_13877526_gb_AF370464.1_AF370464	0.09347	0.37378	2.3647	2.3647	0.005799
958603	At1g09250	264264_at	unknown protein ESTs gb|T04610, gb|N38459, gb|T45174, gb|R30481 and gb|N64971 come from this gene;supported by full-length cDNA: Ceres:9228.	0.06287	0.37008	2.34468	2.34468	8.83E-07
956054	At1g50040	261658_at	hypothetical protein predicted by genemark.hmm	0.0703	0.36172	2.29994	2.29994	4.38E-05
948728	At4g25260	254110_at	putative protein pectinesterase - Citrus sinensis, PID:g2098705	0.05064	0.35825	2.28166	2.28166	5.22E-10
949514	At4g11280	ACS6	ACC synthase (AtACS-6) ; supported by cDNA: gi_16226285_gb_AF428292.1_AF428292	0.06876	0.35412	2.26006	2.26006	4.30E-05
947298	At3g44260	252679_at	CCR4-associated factor 1-like protein CAF1_MOUSE CCR4-ASSOCIATED FACTOR 1 - Mus musculus, SWISSPROT:CAF1_MOUSE; supported by cDNA: gi_15292828_gb_AY050848.1_	0.07738	0.35127	2.24529	2.24529	7.47E-04
946241	At3g58120	251575_at	putative protein basic leucine zipper transcription activator shoot-forming PKSF1 - Paulownia kawakamii, EMBL:AF046934;supported by full-length cDNA: Ceres:34553.	0.01933	0.34899	2.23354	2.23354	0
963768	At4g16770	245307_at	gibberellin oxidase-like protein non-consensus GG acceptor splice site at exon 8; supported by cDNA: gi_13265536_gb_AF324704.2_AF324704	0.07082	0.34677	2.22212	2.22212	1.45E-04
963720	At4g17460	245362_at	homeobox-leucine zipper protein HAT1 (hd-zip protein 1) ;supported by full-length cDNA: Ceres:34167.	0.07806	0.34431	2.20959	2.20959	0.001164
957566	At1g54120	263151_at	hypothetical protein predicted by genemark.hmm;supported by full-length cDNA: Ceres:94743.	0.07426	0.34039	2.18971	2.18971	6.19E-04
951685	At3g19680	257076_at	unknown protein	0.02304	0.33662	2.1708	2.1708	0
950403	At4g10150	255802_s_at	putative protein RING-H2 finger protein RHA1a, Arabidopsis thaliana,AF078683	0.06542	0.33172	2.14643	2.14643	6.38E-05
947379	At3g42800	252765_at	putative protein hypothetical proteins - Arabidopsis thaliana	0.07067	0.3303	2.13945	2.13945	4.14E-04

961547	At2g39870	267339_at	unknown protein ; supported by cDNA: gi_13877742_gb_AF370134.1_AF370134	0.04022	0.32683	2.1224	2.1224	2.19E-13
949688	At4g08950	255064_at	putative phi-1-like phosphate-induced protein ;supported by full-length cDNA: Ceres:3552.	0.07644	0.32665	2.12154	2.12154	0.002152
955401	At1g01260	261050_at	transcription factor MYC7E, putative similar to transcription factor MYC7E GB:AAD15818 GI:4321762 from [Zea mays]	0.0483	0.32079	2.09312	2.09312	9.33E-09
948567	At4g27260	253908_at	GH3 like protein GH3 protein, Glycine max., PIR2:S17433	0.05077	0.32058	2.09207	2.09207	6.89E-08
961537	At2g40000	267357_at	putative nematode-resistance protein ;supported by full-length cDNA: Ceres:35056.	0.07848	0.31771	2.07829	2.07829	0.004959
956885	At1g21820	262488_at	unknown protein EST gb|AA586241 comes from this gene	0.04625	0.31679	2.07391	2.07391	2.42E-09
954129	At1g29510	259783_at	auxin-induced protein, putative similar to SP:P33083 from [Glycine max];supported by full-length cDNA: Ceres:9311.	0.02612	0.31526	2.06663	2.06663	1.63E-30
947325	At3g44610	252646_at	protein kinase-like protein protein kinase - Solanum berthaultii, EMBL:X97980	0.06134	0.3112	2.04736	2.04736	6.33E-05
947827	At4g34760	253255_at	putative auxin-regulated protein auxin-induced protein X15, Glycine max, PIR2:JQ1097;supported by full-length cDNA: Ceres:10510.	0.03856	0.30785	2.03167	2.03167	6.61E-13
946167	At3g58640	251526_at	putative protein several serine/threonine-specific protein kinases	0.02417	0.30465	2.01672	2.01672	2.77E-33
960973	At2g46310	266606_at	putative AP2 domain transcription factor	0.06966	0.30343	2.01106	2.01106	0.001164
956638	At1g70940	262263_at	auxin transport protein REH1, putative similar to auxin transport protein REH1 GI:3377509 from [Oryza sativa]; supported by cDNA: gi_5817300_gb_AF087818.1_AF087818	0.0471	0.30221	2.00542	2.00542	3.80E-08
957228	At1g14920	262850_at	signal response protein (GAI) identical to GAI GB:CAA75492 GI:2569938 [Arabidopsis thaliana] (Genes Dev. In press); supported by cDNA: gi_16648833_gb_AY058194.1_	0.05094	0.29496	1.97224	1.97224	1.54E-06
958046	At1g04240	IAA3	putative auxin-induced protein AUX2-11 Match to Arabidopsis IAA3 (gb|U18406). EST gb|T04296 comes from this gene; supported by cDNA: gi_972910_gb_U18406.1_ATU18406	0.03288	0.29462	1.97069	1.97069	2.30E-16
957387	At1g54200	263002_at	hypothetical protein predicted by genemark.hmm	0.07456	0.29301	1.96341	1.96341	0.008483
957360	At1g23340	263042_at	conserved hypothetical protein protein predicted by genemark.hmm	0.05072	0.28283	1.91792	1.91792	4.82E-06
955579	At1g32920	261193_at	unknown protein ; supported by cDNA: gi_15450636_gb_AY052686.1_	0.06104	0.27498	1.88358	1.88358	8.63E-04
946869	At3g50060	252193_at	R2R3-MYB transcription factor ; supported by cDNA: gi_15983427_gb_AF424588.1_AF424588	0.058	0.27429	1.88056	1.88056	3.23E-04
963920	At2g47440	245176_at	unknown protein similar to GP|2104534|AF001308 (T10M13.11)	0.05325	0.27313	1.87554	1.87554	4.74E-05
946462	At3g54810	251861_at	putative protein GATA transcription factor 3, Arabidopsis thaliana, Y13650; supported by cDNA: gi_15724333_gb_AF412107.1_AF412107	0.06327	0.26995	1.86186	1.86186	0.002152
959567	At2g43060	265245_at	unknown protein	0.06195	0.26928	1.85902	1.85902	0.001164

952279	At3g17100	257894_at	unknown protein ; supported by cDNA: gi_15010765_gb_AY045684.1_	0.05872	0.25471	1.79768	1.79768	0.001164
948483	At4g28240	253812_at	putative wound induced protein wound-induced protein - tomato (fragment), PIR2:S19773;supported by full-length cDNA: Ceres:20161.	0.04443	0.24836	1.77159	1.77159	4.54E-06
953480	At3g02170	259104_at	unknown protein ;supported by full-length cDNA: Ceres:22225.	0.03201	0.24753	1.76819	1.76819	4.31E-12
948053	At4g32290	253475_at	putative protein predicted protein, Arabidopsis thaliana, PATCHX:G2252634	0.04429	0.24711	1.76649	1.76649	4.78E-06
946283	At3g57795	251601_at	Expressed protein ; supported by full-length cDNA: Ceres: 19033.	0.04887	0.24668	1.76473	1.76473	7.02E-05
951749	At3g15210	AtERF4	ethylene responsive element binding factor 4 (AtERF4) identical to GB:BAA32421 from [Arabidopsis thaliana];supported by full-length cDNA: Ceres:22775.	0.04528	0.24103	1.74195	1.74195	1.86E-05
951954	At3g25710	257642_at	putative HLH DNA-binding protein contains Pfam profile: PF00010 helix-loop-helix DNA-binding domain;supported by full-length cDNA: Ceres:247881.	0.04072	0.24024	1.73875	1.73875	8.28E-07
943327	At5g48900	248681_at	pectate lyase non-consensus AG donor splice site at exon 2; supported by full-length cDNA: Ceres: 21311.	0.0583	0.23831	1.73107	1.73107	0.004055
963750	At4g14130	XTR7	xyloglucan endotransglycosylase-related protein XTR-7 ;supported by full-length cDNA: Ceres:33554.	0.04516	0.23671	1.72469	1.72469	2.79E-05
962639	At5g15580	246562_at	putative protein unknown protein F14P3.18 - Arabidopsis thaliana, EMBL:AC009755	0.04365	0.22843	1.69211	1.69211	2.85E-05
946377	At3g55740	ProT2	proline transporter 2	0.03343	0.22805	1.69064	1.69064	2.87E-09
947805	At4g35470	253136_at	putative protein leucine-rich protein - Helianthus annuus (common sunflower),PIR:T12704	0.02854	0.22332	1.67232	1.67232	2.17E-12
963319	At1g73540	245777_at	unknown protein contains similarity to diphosphoinositol polyphosphate phosphohydrolase GI:3978224 from [Homo sapiens]	0.05001	0.22306	1.6713	1.6713	1.04E-03
945272	At5g06930	250653_at	putative protein strong similarity to unknown protein (gb|AAD23715.1)	0.02756	0.21896	1.65562	1.65562	8.85E-13
958273	At2g36220	263931_at	unknown protein ;supported by full-length cDNA: Ceres:12251.	0.04803	0.21795	1.65178	1.65178	7.50E-04
950055	At4g03190	FBL18	F-box protein GRR1-like protein 1, AtFBL18 almost identical to GRR1-like protein 1 GI:12658970 from [Arabidopsis thaliana]	0.0376	0.21538	1.64202	1.64202	2.19E-06
961495	At2g23760	267298_at	putative homeodomain transcription factor ; supported by cDNA: gi_13877512_gb_AF353092.1_AF353092	0.05426	0.2135	1.63492	1.63492	0.007572
952545	At3g13980	258196_at	hypothetical protein predicted by genemark.hmm	0.05211	0.21311	1.63346	1.63346	0.004055
952225	At3g12920	257858_at	hypothetical protein predicted by genefinder;supported by full-length cDNA: Ceres:924.	0.03374	0.21143	1.62717	1.62717	9.15E-08
944270	At5g37770	249583_at	CALMODULIN-RELATED PROTEIN 2, TOUCH-INDUCED (TCH2) ;supported by full-length cDNA: Ceres:25475.	0.04975	0.20846	1.61609	1.61609	0.003153
950517	At1g67050	255854_at	unknown protein ;supported by full-length cDNA: Ceres:12461.	0.0415	0.20798	1.61427	1.61427	8.31E-05
943790	At5g43700	IAA4	auxin-induced protein AUX2-11 (sp P33077)	0.03556	0.19893	1.58098	1.58098	4.50E-06

947535	At4g39400	BRI1	brassinosteroid insensitive 1 gene (BRI1)	0.04839	0.19809	1.57793	1.57793	0.004055
946165	At3g58620	251584_at	putative protein At2g42580 - Arabidopsis thaliana, EMBL:AC007087	0.0364	0.19636	1.57167	1.57167	1.26E-05
953043	At3g08640	258692_at	unknown protein	0.03224	0.1952	1.56746	1.56746	3.35E-07
954598	At1g68550	260209_at	putative AP2 domain transcription factor contains Pfam profile: PF00847 AP2 domain; supported by cDNA: gi_16604674_gb_AY059782.1_	0.04292	0.19388	1.56271	1.56271	8.21E-04
949665	At4g09890	255028_at	putative protein Arabidopsis thaliana chromosome II BAC T30B22 genomic sequence, gene T30B22.22, PID:g2529679	0.02389	0.18674	1.53723	1.53723	2.31E-12
948730	At4g25280	254052_at	UMP/CMP kinase like protein UMP/CMP kinase - Arabidopsis thaliana,PID:g2121275	0.04414	0.18244	1.52208	1.52208	0.004055
960621	At2g46660	266321_at	putative cytochrome P450	0.03504	0.18235	1.52179	1.52179	3.30E-05
942108	At5g62220	247470_at	putative protein various predicted proteins, Arabidopsis thaliana	0.02099	0.17867	1.50894	1.50894	1.01E-14

Shade-regulated in *sav3* seedlings.

Down-regulated genes identified in response to 1h of W+FR in *sav3* mutant seedlings. "Down-regulated" indicates genes that have significantly (BH<0.05) lower signal (Fold change < 1.5) after 1h of W+FR compared to their levels at time 0 (before starting the simulated shade treatment).

Sequence ID	Accession #	Sequence Name(s)	Sequence Description	Log (Error)	Log (Ratio)	Ratio	Fold Change	BH
956329	At1g22550	261924_at	peptide transporter, putative similar to peptide transporter GI:9757725 from [Arabidopsis thaliana]	0.03081	0.17726	1.50404	-1.50404	2.94E-06
947096	At3g47420	252414_at	putative protein sn-glycerol-3-phosphate permease - Haemophilus influenzae,PID:g3603157; supported by cDNA: gi_13430515_gb_AF360170.1_AF360170	0.0375	0.1785	1.50833	-1.50833	3.81E-04
943816	At5g42760	249191_at	putative protein similar to unknown protein (pir||F70811);supported by full-length cDNA: Ceres:111265.	0.05677	0.19494	1.56654	-1.56654	0.04836
963765	At4g15630	245304_at	hypothetical protein ; supported by full-length cDNA: Ceres: 933.	0.02644	0.19543	1.56829	-1.56829	1.22E-10
961524	At2g44230	267344_at	hypothetical protein predicted by genefinder; supported by cDNA: gi_15450386_gb_AY052294.1_	0.04947	0.19557	1.56882	-1.56882	0.00931
960729	At2g38750	ANNAT4	putative annexin ;supported by full-length cDNA: Ceres:32721.	0.04785	0.19597	1.57026	-1.57026	0.005431
948764	At4g24780	254119_at	putative pectate lyase pectate lyase, Musa acuminata, PATX:E209876;supported by full-length cDNA: Ceres:36681.	0.05104	0.19733	1.57517	-1.57517	0.012005
944788	At5g15310	ATMYB16	myb-related protein - like myb-related protein 1, garden petunia, PIR:S26605	0.04831	0.20476	1.60237	-1.60237	0.002982
956611	At1g53870	262259_s_at	hypothetical protein predicted by genemark.hmm;supported by full-length cDNA: Ceres:1094.	0.05499	0.21536	1.64194	-1.64194	0.010113
957334	At1g75710	262969_at	unknown protein	0.04499	0.22126	1.66439	-1.66439	1.89E-04

942870	At5g54130	248191_at	putative protein similar to unknown protein (gb|AAC24386.1)	0.06654	0.23573	1.7208	-1.7208	0.035641
950807	At1g51700	ADOF1	dof zinc finger protein identical to dof zinc finger protein [Arabidopsis thaliana] GI:3608261; supported by cDNA: gi_3608260_dbj_AB017564.1_AB017564	0.06485	0.23804	1.72996	-1.72996	0.023295
960280	At2g15050	LTP	putative lipid transfer protein	0.0145	0.24179	1.74498	-1.74498	0
956567	At1g74940	262170_at	hypothetical protein predicted by genemark.hmm;supported by full-length cDNA: Ceres:24864.	0.03982	0.2447	1.75671	-1.75671	3.64E-07
947259	At3g44990	XTR8	xyloglucan endo-transglycosylase ; supported by cDNA: gi_15810248_gb_AY056163.1_	0.05357	0.25558	1.80129	-1.80129	3.63E-04
948984	At4g21990	APR3	PRH26 protein ;supported by full-length cDNA: Ceres:36866.	0.0516	0.26186	1.82752	-1.82752	8.94E-05
950790	At1g51805	256168_at	receptor protein kinase, putative contains Pfam profiles: PF00069: Eukaryotic protein kinase domain, multiple PF00560: Leucine Rich Repeat	0.03642	0.26445	1.83843	-1.83843	3.03E-10
954728	At1g70560	TAA1	putative alliinase similar to aliinase precursor GB:AAD26853 [Allium cepa]	0.05813	0.28296	1.91851	-1.91851	2.36E-04
946631	At3g52740	252010_at	hypothetical protein ; supported by cDNA: gi_15450654_gb_AY052695.1_	0.08907	0.31302	2.05599	-2.05599	0.038602
943916	At5g41410	BEL1	homeotic protein BEL1 homolog ; supported by cDNA: gi_15146192_gb_AY049237.1_	0.06728	0.31851	2.08214	-2.08214	4.25E-04
941833	At5g64770	247252_at	unknown protein	0.06519	0.33131	2.14443	-2.14443	8.71E-05
961331	At2g23550	MES6	putative acetone-cyanohydrin lyase	0.10345	0.36006	2.29121	-2.29121	0.04193
947308	At3g44450	252661_at	putative protein	0.08385	0.38364	2.419	-2.419	8.33E-04

Up-regulated genes identified in response to 1h of W+FR in *sav3* mutant seedlings. "Up-regulated" indicates genes that have significantly (BH<0.05) higher signal (Fold change > 1.5) after 1h of W+FR compared to their levels at time 0 (before starting the simulated shade treatment).

Sequence ID	Accession #	Sequence Name(s)	Sequence Description	Log (Error)	Log (Ratio)	Ratio	Fold Change	BH
947650	At4g37770	ACS8	1-aminocyclopropane-1-carboxylate synthase - like protein 1-aminocyclopropane-1-carboxylate synthase,Arabidopsis thaliana, S71174; supported by cDNA: gi_12247996_gb_AF334712.1_AF334712	0.29205	-1.42291	0.03776	26.47975	2.32E-04
958701	At1g04180	264323_at	putative dimethylaniline monooxygenase	0.08978	-1.40553	0.03931	25.4407	0
958331	At2g42870	PAR1	unknown protein ;supported by full-length cDNA: Ceres:102453.	0.13641	-1.34676	0.045	22.22088	9.60E-20
963796	At4g16780	ATHB2	DNA-binding homeotic protein Athb-2 ; supported by cDNA: gi_166751_gb_M90394.1_ATHHOMEOA	0.08795	-1.25624	0.05543	18.04007	0
948061	At4g32280	IAA29	Expressed protein ; supported by cDNA: gi_14190492_gb_AF380646.1_AF380646	0.11686	-1.16054	0.0691	14.47241	6.35E-20

945281	At5g07010	ST2A	steroid sulfotransferase-like protein ;supported by full-length cDNA: Ceres:124067.	0.09732	-0.92573	0.11865	8.42812	2.90E-18
953791	At1g02340	HFR1	unknown protein contains similarity to phytochrome interacting factor 3 GI:3929586 from [Arabidopsis thaliana]; supported by cDNA: gi_11870114_gb_AF324245.1_AF324245	0.09933	-0.92268	0.11949	8.36919	2.08E-17
948437	At4g28720	253794_at	putative protein dimethylaniline monooxygenase (N-oxide-forming), Sus scrofa domestica, PIR1:A33768	0.08462	-0.8688	0.13527	7.39272	2.27E-21
955504	At1g75450	CKX5	cytokinin oxidase, putative similar to GB:CAA77151 from [Zea mays] (Plant J. 17 (6), 615-626 (1999))	0.18871	-0.84481	0.14295	6.99537	1.27E-03
943739	At5g44260	249065_at	putative protein similar to unknown protein (gb AAD10689.1); supported by cDNA: gi_14334449_gb_AY034916.1_	0.13879	-0.82669	0.14904	6.70957	1.01E-06
943709	At5g43890	YUC5	dimethylaniline monooxygenase-like	0.09013	-0.78651	0.16349	6.11655	2.72E-15
944978	At5g12050	250327_at	putative serine rich protein predicted proteins, Arabidopsis thaliana;supported by full-length cDNA: Ceres:36958.	0.0598	-0.77865	0.16648	6.00689	2.63E-35
945604	At5g02580	251012_at	putative protein ;supported by full-length cDNA: Ceres:16476.	0.12656	-0.7767	0.16722	5.98	3.76E-07
961174	At2g44910	ATHB4	homeodomain transcription factor (ATHB-4)	0.13383	-0.74727	0.17895	5.58824	6.99E-06
944690	At5g18060	250012_x_at	auxin-induced protein-like	0.0795	-0.73807	0.18278	5.4711	2.08E-17
959648	At2g28400	265276_at	hypothetical protein predicted by genscan and genefinder	0.13297	-0.71918	0.19091	5.23815	1.77E-05
945605	At5g02540	251013_at	putative protein various predicted proteins, Brassica rapa, Arabidopsis thaliana;supported by full-length cDNA: Ceres:26538.	0.06424	-0.68175	0.20809	4.80567	6.64E-23
943496	At5g46240	KAT1	potassium channel protein KAT1 (pir||S32816) ; supported by cDNA: gi_166773_gb_M86990.1_ATHKAT1	0.03508	-0.6808	0.20855	4.7951	0
945276	At5g07000	ST2B	steroid sulfotransferase-like protein	0.04414	-0.67353	0.21206	4.71558	0
942556	At5g57560	TCH4	TCH4 protein (gb|AAA92363.1) ; supported by cDNA: gi_14194112_gb_AF367262.1_AF367262	0.03546	-0.60069	0.25079	3.98737	0
957650	At1g10560	PUB18	putative zinc-binding protein similar to zinc-binding protein (gi|3249068)	0.1583	-0.59902	0.25176	3.97211	0.015552
950307	At3g29575	AFP3	hypothetical protein	0.10041	-0.58633	0.25922	3.85768	1.93E-06
944084	At5g39860	PRE1	putative protein putative DNA-binding protein - Arabidopsis thaliana, EMBL:AC011765;supported by full-length cDNA: Ceres:4734.	0.1432	-0.56592	0.2717	3.68059	0.00931
945642	At5g02200	FHL	hypothetical protein	0.11099	-0.55862	0.2763	3.6193	1.08E-04
953710	At3g03830	259332_at	putative auxin-induced protein similar to SAUR GB:BAA25434 [Raphanus sativus]	0.13098	-0.55837	0.27646	3.61719	0.002982
963299	At1g35140	PHI-1	phosphate-induced (phi-1) protein, putative similar to phi-1 GB:BAA33810 GI:3759184 from [Nicotiana tabacum];supported by full-length cDNA: Ceres:118937.	0.08111	-0.53654	0.29071	3.43987	2.12E-08
955137	At1g15045	260741_at	hypothetical protein contains Pfam profile: PF00117 Glutamine amidotransferase class-I	0.1362	-0.53453	0.29206	3.42395	0.010113

960217	At2g42380	BZIP34	unknown protein ; supported by cDNA: gi_15100052_gb_AF401299.1_AF401299	0.09419	-0.53044	0.29482	3.3919	5.41E-06
950221	At4g01250	WRKY22	putative DNA-binding protein ; supported by cDNA: gi_15028172_gb_AY045909.1_	0.07528	-0.52418	0.2991	3.34332	2.17E-09
941754	At5g66080	PP2C	protein phosphatase 2C-like protein	0.10667	-0.49591	0.31922	3.13266	6.13E-04
953709	At3g03840	259331_at	putative auxin-induced protein similar to SAUR GB:BAA25434 [Raphanus sativus]	0.09502	-0.48356	0.32843	3.04483	8.55E-05
962983	At5g25190	246932_at	ethylene-responsive element - like protein ethylene-responsive element binding protein homolog, Stylosanthes hamata, EMBL:U91857; supported by cDNA: gi_15010715_gb_AY045659.1_	0.12101	-0.48279	0.32901	3.03944	0.008448
942112	At5g62280	247474_at	putative protein predicted proteins, Arabidopsis thaliana	0.09067	-0.48138	0.33008	3.02955	2.92E-05
941705	At5g66590	247074_at	putative protein contains similarity to pathogenesis-related protein;supported by full-length cDNA: Ceres:2152.	0.12407	-0.47516	0.33484	2.98647	0.013922
945190	At5g08130	BIM1	basic helix-loop-helix (bHLH) family protein involved in brassinosteroid signaling.	0.07762	-0.47384	0.33586	2.97742	4.52E-07
952152	At3g23030	IAA2	auxin-inducible gene (IAA2) identical to auxin-inducible gene (IAA2) GB:AF027157 [Arabidopsis thaliana] (Plant Physiol. 115, 1730 (1997))	0.06012	-0.46221	0.34497	2.89878	1.37E-11
952778	At3g15540	IAA19	early auxin-induced protein, IAA19 identical to IAA19 GB:AAB84356 from [Arabidopsis thaliana]; supported by full-length cDNA: Ceres: 94231.	0.0926	-0.44818	0.3563	2.80659	2.67E-04
947838	At4g34770	253207_at	putative protein small auxin up-regulated RNA, Malus domestica, gb:Z93766	0.06761	-0.44791	0.35653	2.80485	2.02E-08
945609	At5g02760	PP2C	protein phosphatase - like protein protein phosphatase 2C homolog, Mesembryanthemum crystallinum, EMBL:AF097667	0.06129	-0.44363	0.36006	2.77734	3.46E-10
949396	At4g13260	YUC2	putative protein dimethylaniline monooxygenase (N-oxide-forming) (EC1.14.13.8) -Oryctolagus cuniculus,PID:g164989	0.04911	-0.44093	0.3623	2.76011	3.12E-16
960446	At2g18790	PHYB	phytochrome B Identical to GB:X17342	0.04763	-0.43579	0.36662	2.72764	6.89E-17
953394	At3g07350	259015_at	unknown protein similar to hypothetical protein GB:AAC17612 [Arabidopsis thaliana];supported by full-length cDNA: Ceres:251012.	0.10825	-0.42895	0.37243	2.68504	0.008448
943399	At5g47370	HAT2	homeobox-leucine zipper protein-like ; supported by cDNA: gi_15450446_gb_AY052324.1_	0.12414	-0.42671	0.37436	2.67124	0.047893
963768	At4g16770	245307_at	gibberellin oxidase-like protein non-consensus GG acceptor splice site at exon 8; supported by cDNA: gi_13265536_gb_AF324704.2_AF324704	0.07728	-0.42224	0.37823	2.64386	1.32E-05
943327	At5g48900	248681_at	pectate lyase non-consensus AG donor splice site at exon 2; supported by full-length cDNA: Ceres: 21311.	0.04619	-0.41236	0.38694	2.58438	4.78E-16
946167	At3g58640	251526_at	putative protein several serine/threonine-specific protein kinases	0.02437	-0.39814	0.39982	2.50113	0
963761	At4g16515	245336_at	Expressed protein ; supported by full-length cDNA: Ceres: 6580.	0.05552	-0.38705	0.41016	2.43809	2.11E-09

948728	At4g25260	254110_at	putative protein pectinesterase - Citrus sinensis, PID:g2098705	0.03984	-0.3726	0.42403	2.3583	1.23E-17
942972	At5g52900	248282_at	unknown protein ;supported by full-length cDNA: Ceres:148254.	0.06324	-0.36708	0.42945	2.32854	2.23E-06
943130	At5g51190	248448_at	putative protein contains similarity to ethylene responsive element binding factor;supported by full-length cDNA: Ceres:2347.	0.08874	-0.36331	0.4332	2.30842	0.005431
948574	At4g27280	253915_at	putative protein centrin, Marsilea vestita;supported by full-length cDNA: Ceres:13072.	0.03668	-0.36307	0.43344	2.3071	8.14E-20
946283	At3g57795	251601_at	Expressed protein ; supported by full-length cDNA: Ceres: 19033.	0.04349	-0.36284	0.43367	2.3059	7.17E-14
947153	At3g45970	ATEXLA1	putative protein cim1 induced allergen, Glycine max, EMBL:U03860;supported by full-length cDNA: Ceres:27534.	0.05056	-0.35777	0.43877	2.27911	1.02E-09
954133	At1g29460	259787_at	auxin-induced protein, putative similar to SP:P33083 from [Glycine max];supported by full-length cDNA: Ceres:147801.	0.09027	-0.35594	0.44062	2.26954	0.00931
961679	At2g45600	267503_at	unknown protein ; supported by full-length cDNA: Ceres:36855.	0.06492	-0.34842	0.44831	2.23058	2.17E-05
964362	At1g29440	257506_at	auxin-induced protein, putative similar to SP:P33083 from [Glycine max]	0.06708	-0.34747	0.44929	2.22571	5.63E-05
942803	At5g54490	PBP1	putative protein similar to unknown protein (pir||T05752);supported by full-length cDNA: Ceres:109272.	0.08667	-0.34378	0.45313	2.20688	0.008448
942241	At5g60860	AtRABA1f	GTP-binding protein - like GTP-binding protein, garden pea, PIR:T06447	0.08209	-0.34172	0.45528	2.19643	0.004224
955188	At1g21910	260856_at	TINY-like protein similar to TINY GB:CAA64359 GI:1246403 from [Arabidopsis thaliana];supported by full-length cDNA: Ceres:19721.	0.08445	-0.34051	0.45655	2.19032	0.007479
959156	At1g03610	264836_at	unknown protein similar to hypothetical protein GB:AAD11584;supported by full-length cDNA: Ceres:123030.	0.05309	-0.33213	0.46544	2.14849	1.96E-07
954130	At1g29450	259784_at	auxin-induced protein, putative similar to SP:P33083 from [Glycine max];supported by full-length cDNA: Ceres:29931.	0.08714	-0.32902	0.46879	2.13316	0.016293
958603	At1g09250	264264_at	unknown protein ESTs gb|T04610, gb|N38459, gb|T45174, gb|R30481 and gb|N64971 come from this gene;supported by full-length cDNA: Ceres:9228.	0.04342	-0.32889	0.46893	2.13252	3.18E-11
955780	At1g21050	261456_at	hypothetical protein predicted by genemark.hmm;supported by full-length cDNA: Ceres:3200.	0.05752	-0.32479	0.47338	2.11248	5.12E-06
960973	At2g46310	CRF5	putative AP2 domain transcription factor	0.05675	-0.32368	0.4746	2.10705	3.81E-06
954119	At1g29500	259773_at	auxin-induced protein, putative similar to SP:P33083 from [Glycine max]	0.06061	-0.32311	0.47522	2.10429	2.62E-05
948483	At4g28240	253812_at	putative wound induced protein wound-induced protein - tomato (fragment), PIR2:S19773;supported by full-length cDNA: Ceres:20161.	0.03378	-0.32122	0.47729	2.09517	2.90E-18
954868	At1g68190	260431_at	putative zinc finger protein similar to zinc finger protein GB:BAA33206 from [Oryza sativa]	0.08224	-0.31761	0.48127	2.07782	0.012005
961547	At2g39870	267339_at	unknown protein ; supported by cDNA: gi_13877742_gb_AF370134.1_AF370134	0.04657	-0.31715	0.48178	2.07562	5.87E-09

951211	At3g30180	BR6OX2	cytochrome P450 homolog, putative similar to cytochrome P450 homolog GB:U54770 GI:1421740 from [Lycopersicon esculentum];supported by full-length cDNA: Ceres:11278.	0.05398	-0.31566	0.48344	2.06852	1.87E-06
945559	At5g03230	250937_at	putative protein various predicted proteins, Arabidopsis thaliana; supported by cDNA: gi_13878024_gb_AF370275.1_AF370275	0.07756	-0.31082	0.48885	2.04561	0.007479
946241	At3g58120	BZIP61	putative protein basic leucine zipper transcription activator shoot-forming PKSF1 - Paulownia kawakamii, EMBL:AF046934;supported by full-length cDNA: Ceres:34553.	0.05373	-0.30278	0.49799	2.00808	5.36E-06
958046	At1g04240	SHY2	putative auxin-induced protein AUX2-11 Match to Arabidopsis IAA3 (gb|U18406). EST gb|T04296 comes from this gene; supported by cDNA: gi_972910_gb_U18406.1_ATU18406	0.05866	-0.30235	0.49848	2.00611	6.37E-05
955662	At1g36940	261292_at	hypothetical protein predicted by genemark.hmm	0.08412	-0.30146	0.49951	2.00197	0.031784
948765	At4g24570	dic-02	putative mitochondrial uncoupling protein mitochondrial uncoupling protein, Arabidopsis thaliana (thale cress), PATX:E1316826;supported by full-length cDNA: Ceres:119476.	0.05329	-0.30144	0.49952	2.00191	4.91E-06
961537	At2g40000	HSPRO2	putative nematode-resistance protein ;supported by full-length cDNA: Ceres:35056.	0.07204	-0.29911	0.50221	1.99119	0.004224
963920	At2g47440	245176_at	unknown protein similar to GP|2104534|AF001308 (T10M13.11)	0.04151	-0.2956	0.50629	1.97516	7.63E-10
947626	At4g38400	ATEXLA2	putative pollen allergen pollen allergen - Pinus radiata, PID:g2935527; supported by cDNA: gi_14190354_gb_AF378855.1_AF378855	0.06437	-0.29474	0.50729	1.97125	8.26E-04
952525	At3g24500	MBF1C	ethylene-responsive transcriptional coactivator, putative similar to GB:AAD46402 from [Lycopersicon esculentum] (Plant J. 18 (6), 589-600 (1999));supported by full-length cDNA: Ceres:158734.	0.06992	-0.29066	0.51208	1.95281	0.004224
953242	At3g05640	258901_at	putative protein phosphatase-2C similar to protein phosphatase-2C GB:AAC36699 from [Mesembryanthemum crystallinum]; supported by cDNA: gi_15215718_gb_AY050388.1_	0.07043	-0.28683	0.51662	1.93567	0.006555
954129	At1g29510	SAUR68	auxin-induced protein, putative similar to SP:P33083 from [Glycine max];supported by full-length cDNA: Ceres:9311.	0.07133	-0.27027	0.5367	1.86323	0.015552
956024	At1g18330	EPR1	hypothetical protein similar to hypothetical protein GB:AAF25987 GI:6714291 from [Arabidopsis thaliana]	0.06876	-0.26726	0.54043	1.85039	0.011127
945983	At3g60390	HAT3	homeobox-leucine zipper protein HAT3 ; supported by cDNA: gi_527632_gb_U09338.1_ATU09338	0.06441	-0.26658	0.54127	1.8475	0.004224
962572	At5g15830	AtbZIP3	bZIP DNA-binding protein-like putative bZIP DNA-binding protein - Capsicum chinense, EMBL:AF127797	0.07396	-0.26362	0.54497	1.83495	0.032846
948053	At4g32290	253475_at	putative protein predicted protein, Arabidopsis thaliana, PATCHX:G2252634	0.05145	-0.26019	0.5493	1.8205	9.74E-05
962317	At4g36780	246284_at	putative protein	0.05653	-0.25485	0.5561	1.79824	1.11E-03

951962	At3g16800	257650_at	protein phosphatase, putative similar to protein phosphatase-2C GB:AAC36699 from [Mesembryanthemum crystallinum];supported by full-length cDNA: Ceres:6305.	0.06536	-0.25216	0.55955	1.78714	0.012005
957387	At1g54200	263002_at	hypothetical protein predicted by genemark.hmm	0.05375	-0.25044	0.56177	1.78009	5.88E-04
956885	At1g21820	262488_at	unknown protein EST gb|AA586241 comes from this gene	0.0401	-0.24876	0.56395	1.77321	2.64E-07
942245	At5g60850	OBP4	zinc finger protein OBP4 - like zinc finger protein OBP4, Arabidopsis thaliana, EMBL:AF155817;supported by full-length cDNA: Ceres:19080.	0.05049	-0.24826	0.5646	1.77116	1.89E-04
946660	At3g53180	251973_at	nodulin / glutamate-ammonia ligase - like protein MtN6 - nodulin 6, Medicago truncatula, EMBL:MET133118	0.04078	-0.24453	0.56947	1.75603	8.16E-07
951685	At3g19680	257076_at	unknown protein	0.04173	-0.2433	0.57109	1.75104	2.00E-06
952586	At3g15760	258275_at	unknown protein ;supported by full-length cDNA: Ceres:8259.	0.04158	-0.24152	0.57343	1.74388	2.20E-06
955579	At1g32920	261193_at	unknown protein ; supported by cDNA: gi_15450636_gb_AY052686.1_	0.05088	-0.24132	0.57369	1.7431	4.11E-04
952545	At3g13980	258196_at	hypothetical protein predicted by genemark.hmm	0.05391	-0.23956	0.57602	1.73605	1.44E-03
955287	At1g02660	260915_at	hypothetical protein similar to hypothetical protein GB:CAB83109 GI:7362739 from [Arabidopsis thaliana]; supported by cDNA: gi_15081706_gb_AY048246.1_	0.0691	-0.23698	0.57945	1.72578	0.04836
952654	At3g15770	258262_at	hypothetical protein	0.05011	-0.23583	0.581	1.72118	4.79E-04
954598	At1g68550	CPuORF53	putative AP2 domain transcription factor contains Pfam profile: PF00847 AP2 domain; supported by cDNA: gi_16604674_gb_AY059782.1_	0.03712	-0.23367	0.58389	1.71264	1.64E-07
949688	At4g08950	EXO	putative phi-1-like phosphate-induced protein ;supported by full-length cDNA: Ceres:3552.	0.04854	-0.23314	0.5846	1.71056	3.18E-04
954610	At1g74670	260221_at	GAST1-like protein similar to GAST1 protein precursor GB:P27057 [Lycopersicon esculentum] (induced by gibberellins, inhibited by ABA Plant J 1992 Mar;2(2):153-9)	0.04671	-0.23056	0.58808	1.70045	1.75E-04
950668	At1g07135	256046_at	unknown protein	0.06275	-0.22982	0.58909	1.69752	0.02396
953627	At3g11490	259287_at	putative rac GTPase activating protein similar to rac GTPase activating protein 1 GB:AAC62624 [Lotus japonicus]	0.04526	-0.22861	0.59074	1.6928	9.89E-05
946880	At3g50340	252204_at	putative protein predicted protein, Arabidopsis thaliana	0.04543	-0.22678	0.59323	1.68569	1.33E-04
945272	At5g06930	250653_at	putative protein strong similarity to unknown protein (gb|AAD23715.1)	0.04324	-0.22612	0.59413	1.68312	4.36E-05
952781	At3g15450	258402_at	unknown protein very similar to unknown protein GB:AAC39468 from [Arabidopsis thaliana]; supported by cDNA: gi_14335087_gb_AY037223.1_	0.06419	-0.2239	0.59717	1.67456	0.04155
957228	At1g14920	GAI	signal response protein (GAI) identical to GAI GB:CAA75492 GI:2569938 [Arabidopsis thaliana] (Genes Dev. In press); supported by cDNA: gi_16648833_gb_AY058194.1_	0.02743	-0.22204	0.59973	1.66741	5.44E-13

958670	At1g78700	264248_at	hypothetical protein predicted by genscan+; supported by cDNA: gi_15215801_gb_AY050430.1_	0.05914	-0.22186	0.59999	1.6667	0.018087
954493	At1g66400	260135_at	calmodulin-related protein similar to GB:P25070 from [Arabidopsis thaliana], contains Pfam profile: PF00036 EF hand (4 copies);supported by full-length cDNA: Ceres:95959.	0.06104	-0.22169	0.60023	1.66604	0.026501
952279	At3g17100	257894_at	unknown protein ; supported by cDNA: gi_15010765_gb_AY045684.1_	0.05358	-0.22015	0.60236	1.66014	0.005431
943481	At5g47380	248784_at	putative protein similar to unknown protein (pir||T02421)	0.05313	-0.21758	0.60592	1.65038	0.005431
958782	At1g11960	264389_at	unknown protein similar to hypothetical protein HYP1 gb|Z97338 from A. thaliana	0.04697	-0.21736	0.60624	1.64952	6.72E-04
954136	At1g29430	259790_s_at	auxin-induced protein, putative similar to SP:P33083 from [Glycine max];supported by full-length cDNA: Ceres:4119.	0.06228	-0.2169	0.60687	1.64779	0.04193
954001	At1g69010	BIM2	putative DNA-binding protein predicted by genscan, multiple est matches; Pfam HMM hit: helix-loop-helix DNA-binding domain;supported by full-length cDNA: Ceres:27793.	0.05332	-0.21443	0.61033	1.63845	0.007479
947483	At4g39800	MIPS1	myo-inositol-1-phosphate synthase ;supported by full-length cDNA: Ceres:1978.	0.03888	-0.21391	0.61107	1.63648	1.09E-05
956638	At1g70940	PIN3	auxin transport protein REH1, putative similar to auxin transport protein REH1 GI:3377509 from [Oryza sativa]; supported by cDNA: gi_5817300_gb_AF087818.1_AF087818	0.05056	-0.21298	0.61237	1.63299	0.004224
953484	At3g02140	TMAC2	hypothetical protein predicted by genscan; supported by cDNA: gi_14334515_gb_AY034949.1_	0.05788	-0.2119	0.6139	1.62893	0.02396
942391	At5g58650	PSY1	putative protein ; supported by cDNA: gi_15529263_gb_AY052256.1_	0.0531	-0.21178	0.61408	1.62845	0.008448
946828	At3g50660	DWF4	steroid 22-alpha-hydroxylase (DWF4) ; supported by cDNA: gi_15724347_gb_AF412114.1_AF412114	0.06033	-0.21027	0.61621	1.62282	0.04155
955401	At1g01260	261050_at	transcription factor MYC7E, putative similar to transcription factor MYC7E GB:AAD15818 GI:4321762 from [Zea mays]	0.05387	-0.21003	0.61655	1.62193	0.011127
946462	At3g54810	BME3	putative protein GATA transcription factor 3, Arabidopsis thaliana, Y13650; supported by cDNA: gi_15724333_gb_AF412107.1_AF412107	0.03643	-0.20245	0.6274	1.59387	7.98E-06
942229	At5g60680	247585_at	putative protein predicted proteins, Arabidopsis thaliana;supported by full-length cDNA: Ceres:16638.	0.02781	-0.19959	0.63156	1.58339	5.29E-10
942603	At5g56980	247933_at	putative protein non-consensus CG donor splice site at exon 1, GA donor splice site at exon 3, similar to unknown protein (pir||T04268);supported by full-length cDNA: Ceres:32257.	0.03849	-0.1982	0.63358	1.57834	6.50E-05
944984	At5g11970	250301_at	putative protein predicted proteins, Arabidopsis thaliana; supported by full-length cDNA: Ceres: 13625.	0.03599	-0.19762	0.63442	1.57624	1.14E-05
948997	At4g22190	254356_at	hypothetical protein ;supported by full-length cDNA: Ceres:6848.	0.04448	-0.19728	0.63491	1.57502	1.49E-03

946165	At3g58620	TTL4	putative protein At2g42580 - Arabidopsis thaliana, EMBL:AC007087	0.03403	-0.19681	0.63561	1.57329	2.50E-06
949514	At4g11280	ACS6	ACC synthase (AtACS-6) ; supported by cDNA: gi_16226285_gb_AF428292.1_AF428292	0.05015	-0.19599	0.63681	1.57034	0.010113
947827	At4g34760	253255_at	putative auxin-regulated protein auxin-induced protein X15, Glycine max, PIR2:JQ1097;supported by full-length cDNA: Ceres:10510.	0.05159	-0.19355	0.6404	1.56153	0.018087
956582	At1g68560	XYL1	alpha-xylosidase precursor identical to alpha-xylosidase precursor GB:AAD05539 GI:4163997 from [Arabidopsis thaliana]; supported by cDNA: gi_15982750_gb_AY057482.1_	0.02615	-0.19313	0.64101	1.56003	1.25E-10
945912	At3g61830	ARF18	auxin response factor-like protein auxin response factor 9 - Arabidopsis thaliana, PIR:T08917; supported by cDNA: gi_16604602_gb_AY059746.1_	0.05482	-0.19205	0.64261	1.55615	0.039896
955859	At1g21060	261451_at	unknown protein	0.05251	-0.18749	0.64939	1.5399	0.032846
960949	At2g14900	266613_at	similar to gibberellin-regulated proteins	0.04667	-0.18738	0.64956	1.53949	0.007479
948567	At4g27260	WES1	GH3 like protein GH3 protein, Glycine max., PIR2:S17433	0.03459	-0.18621	0.65132	1.53535	2.00E-05
948150	At4g31390	253517_at	predicted protein hypothetical protein slr1919, Synechocystis sp., PIR2:S75233	0.03991	-0.18371	0.65508	1.52653	7.41E-04
954685	At1g80280	260297_at	unknown protein contains Pfam profile: PF00561 alpha/beta hydrolase fold	0.02881	-0.1807	0.65964	1.51599	1.82E-07
953480	At3g02170	LNG2	unknown protein ;supported by full-length cDNA: Ceres:22225.	0.0287	-0.18007	0.66059	1.5138	1.82E-07
955218	At1g06850	AtbZIP52	b-Zip DNA binding protein, putative similar to b-Zip DNA binding protein GB:CAB06697 GI:2246376 from [Arabidopsis thaliana]	0.04299	-0.17873	0.66263	1.50915	0.004224
953667	At3g05120	GID1A	unknown protein	0.03654	-0.17771	0.66419	1.50559	2.41E-04
961901	At5g28300	245861_at	GTL1 - like protein GTL1, Arabidopsis thaliana, EMBL:ATAJ3215; supported by cDNA: gi_14423451_gb_AF386963.1_AF386963	0.05079	-0.17694	0.66536	1.50295	0.04155

Shade-regulated in *sav1* seedlings.

Down-regulated genes identified in response to 1h of W+FR in *sav1* mutant seedlings. "Down-regulated" indicates genes that have significantly (BH<0.05) lower signal (Fold change < 1.5) after 1h of W+FR compared to their levels at time 0 (before starting the simulated shade treatment).

Sequence ID	Accession #	Sequence Name(s)	Sequence Description	Log (Error)	Log (Ratio)	Ratio	Fold Change	BH
961502	At2g30070	ATKT1	high affinity K+ transporter (AtKUP1/AtKT1p) identical to GB:AF029876; supported by cDNA: gi_2654087_gb_AF033118.1_AF033118	0.04334	0.17748	1.50482	-1.50482	0.002549
942203	At5g61420	MYB28	putative transcription factor MYB28 ; supported by cDNA: gi_5823328_gb_AF175998.1_AF175998	0.04209	0.17765	1.50539	-1.50539	0.001358
959591	At2g36630	265203_at	unknown protein	0.05103	0.1787	1.50902	-1.50902	0.021905

953084	At3g05800	258742_at	hypothetical protein predicted by genscan+;supported by full-length cDNA: Ceres:21672.	0.0499	0.17909	1.51039	-1.51039	0.016617
955473	At1g19700	BEL10	homeodomain protein, putative similar to homeodomain protein GI:7239157 from (Malus domestica)	0.03679	0.17973	1.51261	-1.51261	9.72E-05
961969	At5g11060	KNAT4	HOMEOBOX PROTEIN KNOTTED-1 LIKE 4 (KNAT4)	0.04187	0.18099	1.51701	-1.51701	0.001358
962303	At4g36500	246270_at	putative protein	0.02825	0.18715	1.53867	-1.53867	6.25E-09
945321	At5g06270	250734_at	putative protein similar to unknown protein (gb|AAF02129.1);supported by full-length cDNA: Ceres:104017.	0.02748	0.1925	1.55776	-1.55776	4.85E-10
954557	At1g70700	TIFY7	hypothetical protein predicted by genefinder	0.04445	0.19635	1.57162	-1.57162	7.11E-04
958466	At2g28550	RAP2.7	putative AP2 domain transcription factor pFAM domain (PF00847); supported by cDNA: gi_15292762_gb_AY050815.1_	0.03481	0.19721	1.57474	-1.57474	1.91E-06
941833	At5g64770	247252_at	unknown protein	0.04782	0.19879	1.5805	-1.5805	0.00195
956329	At1g22550	261924_at	peptide transporter, putative similar to peptide transporter GI:9757725 from [Arabidopsis thaliana]	0.03111	0.19971	1.58385	-1.58385	2.22E-08
948764	At4g24780	254119_at	putative pectate lyase pectate lyase, Musa acuminata, PATX:E209876;supported by full-length cDNA: Ceres:36681.	0.04164	0.2	1.58489	-1.58489	1.40E-04
944216	At5g38220	249551_at	putative protein predicted proteins, Arabidopsis thaliana	0.03912	0.201	1.58855	-1.58855	2.81E-05
960783	At2g22770	NAI1	putative bHLH transcription factor	0.03672	0.20136	1.58986	-1.58986	5.02E-06
961409	At2g37710	RLK	putative receptor-like protein kinase same as GB:X95909 (polymorphism exists at a GA repeat. We found 6 copies in our sequence whereas only 5 copies exist in GB:X95909)	0.03961	0.20768	1.61318	-1.61318	1.72E-05
957125	At1g13100	CYP71B29	putative cytochrome P450 monooxygenase strong similarity to gb|X97864 cytochrome P450 from Arabidopsis thaliana and is a member of the PF|00067 Cytochrome P450 family	0.04672	0.2108	1.62481	-1.62481	5.08E-04
955903	At1g01140	CIPK9	serine/threonine kinase, putative similar to serine/threonine kinase GB:CAA73067.1 GI:2632252 from [Sorghum bicolor]; supported by cDNA: gi_14423523_gb_AF386999.1_AF386999	0.0416	0.2121	1.62969	-1.62969	3.45E-05
947936	At4g33960	253317_at	putative protein	0.04893	0.21312	1.63351	-1.63351	0.000711
959971	At2g20180	PIL5	unknown protein	0.05866	0.21372	1.63577	-1.63577	0.013965
947271	At3g45210	252619_at	putative protein several hypothetical proteins - Arabidopsis thaliana; supported by cDNA: gi_15724293_gb_AF412087.1_AF412087	0.0369	0.2146	1.63909	-1.63909	8.22E-07
944788	At5g15310	ATMYB16	myb-related protein - like myb-related protein 1, garden petunia, PIR:S26605	0.06469	0.21497	1.64047	-1.64047	0.038965
957126	At1g13110	CYP71B7	putative cytochrome P450 monooxygenase identical to gb|X97864 cytochrome P450 from Arabidopsis thaliana and is a member of the PF|00067 Cytochrome P450 family. ESTs gb|T44875, gb|T04814, gb|R65111, gb|T44310 and gb|T04541 come from this gene	0.02902	0.21577	1.64349	-1.64349	2.28E-11

957099	At1g28600	262745_at	lipase, putative contains Pfam profile: PF00657 Lipase/Acylhydrolase with GDSL-like motif;supported by full-length cDNA: Ceres:37307.	0.0577	0.21856	1.65409	-1.65409	0.008305
955114	At1g49230	260753_at	RING-H2 finger protein RHA3a, putative similar to RING-H2 finger protein RHA3a GI:3790573 from [Arabidopsis thaliana]	0.04937	0.21879	1.65497	-1.65497	7.11E-04
946812	At3g50440	MES10	putative protein pir7a protein - Oryza sativa, PIR:s47086;supported by full-length cDNA: Ceres:30104.	0.04742	0.22082	1.66273	-1.66273	2.73E-04
952756	At3g16560	258437_at	unknown protein contains protein phosphatase 2C domain; supported by full-length cDNA: Ceres: 249756.	0.03483	0.22116	1.66403	-1.66403	3.40E-08
949499	At4g12310	CYP706A5	flavonoid 3,5-hydroxylase -like protein flavonoid 3',5'-hydroxylase - Campanula medium, PID:d1003951	0.0495	0.2225	1.66918	-1.66918	5.49E-04
955742	At1g18810	261407_at	unknown protein ;supported by full-length cDNA: Ceres:151637.	0.05678	0.22316	1.67172	-1.67172	0.004655
948032	At4g33040	253382_at	putative protein AT.I.24, Arabidopsis thaliana, gb:U63815;supported by full-length cDNA: Ceres:4868.	0.0607	0.2233	1.67225	-1.67225	0.012258
947096	At3g47420	252414_at	putative protein sn-glycerol-3-phosphate permease - Haemophilus influenzae,PID:g3603157; supported by cDNA: gi_13430515_gb_AF360170.1_AF360170	0.04	0.2246	1.67725	-1.67725	2.47E-06
958564	At1g54820	264240_at	protein kinase, putative Simisimilar to protein kinase 2 GI:7573598 from [Populus nigra]	0.03194	0.22526	1.67981	-1.67981	3.47E-10
955636	At1g20190	ATEXPA11	expansin S2 precursor, putative similar to GB:U30460 from [Cucumis sativus];supported by full-length cDNA: Ceres:11011.	0.05851	0.22659	1.68498	-1.68498	0.006273
963765	At4g15630	245304_at	hypothetical protein ; supported by full-length cDNA: Ceres: 933.	0.02842	0.22667	1.68528	-1.68528	3.98E-13
948984	At4g21990	APR3	PRH26 protein ;supported by full-length cDNA: Ceres:36866.	0.04851	0.22701	1.68658	-1.68658	2.48E-04
954461	At1g78170	260081_at	unknown protein	0.03006	0.22743	1.68823	-1.68823	8.98E-12
960258	At2g19650	265939_at	putative Ta11-like non-LTR retroelement protein similar to GB:AAA75253; some members of this protein family have a weak CCHC zinc fingers that is mostly from retroviral gag proteins (nucleocapsid)	0.04838	0.22772	1.68936	-1.68936	2.19E-04
949219	At4g19380	254572_at	putative protein predicted protein MTCY20G9, Mycobacterium tuberculosis	0.05064	0.22932	1.69557	-1.69557	4.75E-04
945703	At5g01820	ATSR1	putative protein serine/threonine protein kinase ATPK10 - Arabidopsis thaliana, EMBL:D30622; supported by cDNA: gi_13249126_gb_AF295669.1_AF295669	0.05876	0.23105	1.70235	-1.70235	0.004655
957263	At1g64710	ADH	alcohol dehydrogenase, putative similar to alcohol dehydrogenase GI:551256 from [Nicotiana tabacum]	0.06705	0.23374	1.71291	-1.71291	0.023141
961180	At2g44940	266820_at	putative AP2 domain transcription factor pFAM domain (PF00847)supported by full-length cDNA: Ceres:31044.	0.04501	0.23542	1.71959	-1.71959	1.82E-05
956953	At1g15260	262598_at	unknown protein EST gb|N65467 comes from this gene;supported by full-length cDNA: Ceres:30239.	0.0197	0.2358	1.72107	-1.72107	4.18E-30

957334	At1g75710	262969_at	unknown protein	0.04499	0.23594	1.72163	-1.72163	1.72E-05
947250	At3g44970	252629_at	cytochrome P450 - like protein cytochrome P450 d13695c, Arabidopsis thaliana, PIR:C71417	0.06694	0.2378	1.729	-1.729	0.018802
941671	At5g67280	RLK	receptor-like protein kinase ;supported by full-length cDNA: Ceres:110712.	0.04106	0.23948	1.73574	-1.73574	7.50E-07
963129	At2g39250	SNZ	putative AP2 domain transcription factor pFAM domain (PF00847)	0.06232	0.24099	1.74175	-1.74175	0.006273
942715	At5g55620	248028_at	putative protein similar to unknown protein (gb|AAF04428.1);supported by full-length cDNA: Ceres:27668.	0.06711	0.24157	1.74409	-1.74409	0.01622
959806	At2g20950	265433_at	unknown protein	0.05615	0.2478	1.76931	-1.76931	0.000711
953273	At3g10525	258919_at	Expressed protein ; supported by full-length cDNA: Ceres: 2153.	0.05663	0.24863	1.7727	-1.7727	0.000711
960729	At2g38750	ANNAT4	putative annexin ;supported by full-length cDNA: Ceres:32721.	0.05566	0.25841	1.81306	-1.81306	2.88E-04
944325	At5g24570	249750_at	unknown protein ;supported by full-length cDNA: Ceres:141953.	0.06965	0.26554	1.84307	-1.84307	0.007789
944663	At5g17700	250045_at	putative protein mRNA (orf04), Arabidopsis thaliana, EMBL:ATORF04	0.08115	0.27291	1.8746	-1.8746	0.034371
947259	At3g44990	XTR8	xyloglucan endo-transglycosylase ; supported by cDNA: gi_15810248_gb_AY056163.1_	0.07652	0.27773	1.89555	-1.89555	0.014352
954728	At1g70560	TAA1	putative alliinase similar to aliinase precursor GB:AAD26853 [Allium cepa]	0.05605	0.31308	2.05627	-2.05627	2.87E-06
956567	At1g74940	262170_at	hypothetical protein predicted by genemark.hmm;supported by full-length cDNA: Ceres:24864.	0.03482	0.32434	2.11029	-2.11029	4.84E-18
960280	At2g15050	LTP	putative lipid transfer protein	0.01596	0.32879	2.13204	-2.13204	0
950807	At1g51700	ADOF1	dof zinc finger protein identical to dof zinc finger protein [Arabidopsis thaliana] GI:3608261; supported by cDNA: gi_3608260_dbj_AB017564.1_AB017564	0.06588	0.33229	2.14925	-2.14925	4.51E-05
959272	At2g17300	264909_at	unknown protein	0.09132	0.33868	2.18115	-2.18115	0.011271
943916	At5g41410	BEL1	homeotic protein BEL1 homolog ; supported by cDNA: gi_15146192_gb_AY049237.1_	0.06231	0.35067	2.24218	-2.24218	2.33E-06
950790	At1g51805	256168_at	receptor protein kinase, putative contains Pfam profiles: PF00069: Eukaryotic protein kinase domain, multiple PF00560: Leucine Rich Repeat	0.03805	0.35914	2.28635	-2.28635	1.61E-18
947308	At3g44450	252661_at	putative protein	0.0864	0.48008	3.02048	-3.02048	3.37E-06
941718	At5g66740	247055_at	putative protein similar to unknown protein (emb|CAB81597.1)	0.13948	0.48629	3.06404	-3.06404	0.023141

Up-regulated genes identified in response to 1h of W+FR in *sav1* mutant seedlings. "Up-regulated" indicates genes that have significantly (BH<0.05) higher signal (Fold change > 1.5) after 1h of W+FR compared to their levels at time 0 (before starting the simulated shade treatment).

Sequence ID	Accession #	Sequence Name(s)	Sequence Description	Log (Error)	Log (Ratio)	Ratio	Fold Change	BH

947650	At4g37770	ACS8	1-aminocyclopropane-1-carboxylate synthase - like protein 1-aminocyclopropane-1-carboxylate synthase,Arabidopsis thaliana, S71174; supported by cDNA: gi_12247996_gb_AF334712.1_AF334712	0.2316	-1.75386	0.01763	56.73592	8.58E-12
958701	At1g04180	264323_at	putative dimethylaniline monooxygenase	0.09903	-1.31711	0.04818	20.75463	2.66E-37
948061	At4g32280	IAA29	Expressed protein ; supported by cDNA: gi_14190492_gb_AF380646.1_AF380646	0.0593	-1.31205	0.04875	20.51417	0
963796	At4g16780	ATHB2	DNA-binding homeotic protein Athb-2 ; supported by cDNA: gi_166751_gb_M90394.1_ATHHOMEOA	0.0937	-1.22452	0.05963	16.76968	5.39E-36
958331	At2g42870	PAR1	unknown protein ;supported by full-length cDNA: Ceres:102453.	0.12241	-1.15001	0.07079	14.12583	2.34E-18
948437	At4g28720	253794_at	putative protein dimethylaniline monooxygenase (N-oxide-forming), Sus scrofa domestica, PIR1:A33768	0.08012	-1.14523	0.07158	13.97101	0
952778	At3g15540	IAA19	early auxin-induced protein, IAA19 identical to IAA19 GB:AAB84356 from [Arabidopsis thaliana]; supported by full-length cDNA: Ceres: 94231.	0.04968	-1.12475	0.07503	13.32754	0
954520	At1g52830	IAA6	putative IAA6 protein similar to IAA6 protein GB:S5849 from [Arabidopsis thaliana];supported by full-length cDNA: Ceres:147711.	0.08839	-1.05175	0.08877	11.26547	9.78E-30
943073	At5g51810	GA20OX2	gibberellin 20-oxidase (emb|CAA58294.1)	0.1129	-1.00195	0.09955	10.04501	2.46E-16
956171	At1g15580	IAA5	auxin-induced protein IAA5, putative similar to auxin-induced protein IAA5 GI:972913 from [Arabidopsis thaliana]	0.08139	-0.99951	0.10011	9.98862	1.02E-31
953791	At1g02340	HFR1	unknown protein contains similarity to phytochrome interacting factor 3 GI:3929586 from [Arabidopsis thaliana]; supported by cDNA: gi_11870114_gb_AF324245.1_AF324245	0.09663	-0.9834	0.1039	9.62509	1.36E-21
949810	At4g08040	ACS11	strong similarity to 1-aminocyclopropane-1-carboxylic acid synthases	0.13079	-0.96742	0.10779	9.2772	3.04E-11
945801	At3g63440	CKX6	cytokinin oxidase -like protein cytokinin oxidase, Zea mays, EMBL:ZMY18377	0.10035	-0.95484	0.11096	9.01231	7.79E-19
942972	At5g52900	248282_at	unknown protein ;supported by full-length cDNA: Ceres:148254.	0.03576	-0.93807	0.11533	8.67107	0
944690	At5g18060	250012_x_at	auxin-induced protein-like	0.05629	-0.91982	0.12028	8.31416	0
942112	At5g62280	247474_at	putative protein predicted proteins, Arabidopsis thaliana	0.04663	-0.90911	0.12328	8.11174	0
943709	At5g43890	YUC5	dimethylaniline monooxygenase-like	0.1321	-0.9016	0.12543	7.97251	1.67E-09
963299	At1g35140	PHI-1	phosphate-induced (phi-1) protein, putative similar to phi-1 GB:BAA33810 GI:3759184 from [Nicotiana tabacum];supported by full-length cDNA: Ceres:118937.	0.08185	-0.88768	0.12952	7.72109	1.38E-24
946399	At3g55840	251774_at	nematode resistance protein-like protein Hs1pro-1 nematode resistance gene, Beta procumbens, EMBL:BPU79733;supported by full-length cDNA: Ceres:149697.	0.10199	-0.88129	0.13143	7.60834	1.84E-15
943399	At5g47370	HAT2	homeobox-leucine zipper protein-like ; supported by cDNA: gi_15450446_gb_AY052324.1_	0.05954	-0.86988	0.13493	7.41109	0

942556	At5g57560	TCH4	TCH4 protein (gb|AAA92363.1) ; supported by cDNA: gi_14194112_gb_AF367262.1_AF367262	0.03265	-0.86658	0.13596	7.3549	0
953710	At3g03830	259332_at	putative auxin-induced protein similar to SAUR GB:BAA25434 [Raphanus sativus]	0.07365	-0.85466	0.13975	7.15576	2.86E-28
945609	At5g02760	PP2C	protein phosphatase - like protein protein phosphatase 2C homolog, Mesembryanthemum crystallinum, EMBL:AF097667	0.0428	-0.83338	0.14676	6.81373	0
943739	At5g44260	249065_at	putative protein similar to unknown protein (gb AAD10689.1); supported by cDNA: gi_14334449_gb_AY034916.1_	0.07633	-0.80716	0.1559	6.41453	2.34E-23
945281	At5g07010	ST2A	steroid sulfotransferase-like protein ;supported by full-length cDNA: Ceres:124067.	0.11132	-0.79889	0.15889	6.2935	1.48E-10
945642	At5g02200	FHL	hypothetical protein	0.12369	-0.79193	0.16146	6.19341	2.46E-08
958225	At2g37030	263890_at	putative auxin-induced protein	0.12761	-0.78783	0.16299	6.13516	1.01E-07
945604	At5g02580	251012_at	putative protein ;supported by full-length cDNA: Ceres:16476.	0.17813	-0.78745	0.16314	6.12983	7.11E-04
942799	At5g54470	248160_at	putative protein similar to unknown protein (pir||T05755)	0.2048	-0.78114	0.16553	6.04137	0.007789
961174	At2g44910	ATHB4	homeodomain transcription factor (ATHB-4)	0.15729	-0.77251	0.16885	5.92255	8.60E-05
964006	At2g23170	GH3.3	unknown protein	0.02785	-0.75734	0.17485	5.71921	0
955504	At1g75450	CKX5	cytokinin oxidase, putative similar to GB:CAA77151 from [Zea mays] (Plant J. 17 (6), 615-626 (1999))	0.13363	-0.74876	0.17833	5.60744	2.60E-06
953709	At3g03840	259331_at	putative auxin-induced protein similar to SAUR GB:BAA25434 [Raphanus sativus]	0.07717	-0.7418	0.18122	5.51824	3.15E-19
954228	At1g69570	259834_at	H-protein promoter binding factor-2b nearly identical to H-protein promoter binding factor-2b (Arabidopsis thaliana) GI:3386548; supported by cDNA: gi_3386547_gb_AF079504.1_AF079504	0.15254	-0.73349	0.18472	5.41367	1.37E-04
948743	At4g25420	GA20OX1	gibberellin 20-oxidase - Arabidopsis thaliana	0.14163	-0.73206	0.18533	5.39586	2.45E-05
944978	At5g12050	250327_at	putative serine rich protein predicted proteins, Arabidopsis thaliana;supported by full-length cDNA: Ceres:36958.	0.05214	-0.71671	0.19199	5.20851	6.45E-40
943496	At5g46240	KAT1	potassium channel protein KAT1 (pir||S32816) ; supported by cDNA: gi_166773_gb_M86990.1_ATHKAT1	0.04344	-0.69389	0.20235	4.94185	0
947153	At3g45970	ATEXLA1	putative protein cim1 induced allergen, Glycine max, EMBL:U03860;supported by full-length cDNA: Ceres:27534.	0.04036	-0.69379	0.2024	4.94073	0
963695	At4g14560	IAA1	auxin-responsive protein IAA1 ;supported by full-length cDNA: Ceres:33860.	0.06853	-0.69159	0.20343	4.91575	3.13E-21
945605	At5g02540	251013_at	putative protein various predicted proteins, Brassica rapa, Arabidopsis thaliana;supported by full-length cDNA: Ceres:26538.	0.06792	-0.69035	0.20401	4.90176	1.52E-21
956093	At1g18400	BEE1	helix-loop-helix protein homolog, putative similar to helix-loop-helix protein homolog GB:BAA87957 GI:6520231 from [Arabidopsis thaliana]	0.08144	-0.67563	0.21104	4.73837	3.17E-14
945276	At5g07000	ST2B	steroid sulfotransferase-like protein	0.04117	-0.65765	0.21996	4.54622	0

941705	At5g66590	247074_at	putative protein contains similarity to pathogenesis-related protein;supported by full-length cDNA: Ceres:2152.	0.08372	-0.64575	0.22607	4.42333	2.93E-12
951396	At3g29370	256743_at	Expressed protein ; supported by full-length cDNA: Ceres: 22461.	0.12299	-0.63371	0.23243	4.30236	2.62E-05
960217	At2g42380	BZIP34	unknown protein ; supported by cDNA: gi_15100052_gb_AF401299.1_AF401299	0.09474	-0.62611	0.23653	4.22779	6.79E-09
956322	At1g65920	261917_at	hypothetical protein similar to TMV resistance protein-like GI:9757959 from [Arabidopsis thaliana]	0.10716	-0.61224	0.24421	4.09482	1.46E-06
944868	At5g13700	ATPAO1	polyamine oxidase	0.11331	-0.60921	0.24592	4.06644	8.76E-06
952152	At3g23030	IAA2	auxin-inducible gene (IAA2) identical to auxin-inducible gene (IAA2) GB:AF027157 [Arabidopsis thaliana] (Plant Physiol. 115, 1730 (1997))	0.03852	-0.60891	0.24609	4.06357	0
962983	At5g25190	246932_at	ethylene-responsive element - like protein ethylene-responsive element binding protein homolog, Stylosanthes hamata, EMBL:U91857; supported by cDNA: gi_15010715_gb_AY045659.1_	0.08963	-0.60763	0.24681	4.05166	2.26E-09
953248	At3g06370	NHX4	putative sodium proton exchanger similar to sodium proton exchanger (Nhx1) GB:AAD16946 [Arabidopsis thaliana]	0.10789	-0.58723	0.25868	3.86574	6.26E-06
947626	At4g38400	ATEXLA2	putative pollen allergen pollen allergen - Pinus radiata, PID:g2935527; supported by cDNA: gi_14190354_gb_AF378855.1_AF378855	0.05973	-0.58586	0.2595	3.85352	5.00E-20
947778	At4g36110	253103_at	putative auxin-induced protein high similarity to auxin-induced protein 15A, soybean, PIR2:JQ1096; supported by cDNA: gi_13194817_gb_AF349524.1_AF349524	0.10195	-0.5845	0.26032	3.84145	1.31E-06
950221	At4g01250	WRKY22	putative DNA-binding protein ; supported by cDNA: gi_15028172_gb_AY045909.1_	0.07375	-0.57894	0.26367	3.79263	1.05E-12
943913	At5g41400	249306_at	RING zinc finger protein-like ;supported by full-length cDNA: Ceres:207148.	0.05965	-0.57543	0.26581	3.76211	2.31E-19
944084	At5g39860	PRE1	putative protein putative DNA-binding protein - Arabidopsis thaliana, EMBL:AC011765;supported by full-length cDNA: Ceres:4734.	0.13735	-0.57466	0.26628	3.75541	0.00195
961807	At2g26710	BAS1	putative cytochrome P450	0.06693	-0.57449	0.26638	3.754	2.98E-15
955287	At1g02660	260915_at	hypothetical protein similar to hypothetical protein GB:CAB83109 GI:7362739 from [Arabidopsis thaliana]; supported by cDNA: gi_15081706_gb_AY048246.1_	0.06301	-0.56501	0.27227	3.67287	1.11E-16
946099	At3g59900	ARGOS	putative protein hypothetical protein At2g44080 - Arabidopsis thaliana, EMBL:AC004005	0.07852	-0.56402	0.27288	3.66458	1.43E-10
954131	At1g29490	259785_at	unknown protein ;supported by full-length cDNA: Ceres:252195.	0.08536	-0.55599	0.27798	3.59739	1.23E-08
963761	At4g16515	245336_at	Expressed protein ; supported by full-length cDNA: Ceres: 6580.	0.04268	-0.5521	0.28048	3.56535	2.91E-35
954133	At1g29460	259787_at	auxin-induced protein, putative similar to SP:P33083 from [Glycine max];supported by full-length cDNA: Ceres:147801.	0.07103	-0.54867	0.2827	3.53732	2.72E-12
964362	At1g29440	257506_at	auxin-induced protein, putative similar to SP:P33083 from [Glycine max]	0.04751	-0.548	0.28314	3.5318	6.17E-28

948574	At4g27280	253915_at	putative protein centrin, Marsilea vestita;supported by full-length cDNA: Ceres:13072.	0.03281	-0.54266	0.28664	3.48865	0
959648	At2g28400	265276_at	hypothetical protein predicted by genscan and genefinder	0.08643	-0.53698	0.29042	3.44334	8.01E-08
947838	At4g34770	253207_at	putative protein small auxin up-regulated RNA, Malus domestica, gb:Z93766	0.0641	-0.53052	0.29477	3.3925	3.71E-14
960166	At2g18010	265806_at	putative auxin-regulated protein	0.10682	-0.52384	0.29934	3.34074	8.89E-05
952200	At3g28420	257900_at	hypothetical protein predicted by genemark.hmm	0.08066	-0.52374	0.2994	3.33998	1.40E-08
947379	At3g42800	252765_at	putative protein hypothetical proteins - Arabidopsis thaliana	0.06566	-0.52239	0.30034	3.32956	4.61E-13
946880	At3g50340	252204_at	putative protein predicted protein, Arabidopsis thaliana	0.03713	-0.52077	0.30146	3.31715	1.42E-41
960926	At2g35290	266545_at	hypothetical protein predicted by genefinder	0.12754	-0.51591	0.30485	3.2803	0.003082
961190	At2g22810	ACS4	1-aminocyclopropane-1-carboxylate synthase (ACS4) identical to GB:U23481; supported by cDNA: gi_12083215_gb_AF332404.1_AF332404	0.15676	-0.51097	0.30834	3.24317	0.047397
943130	At5g51190	248448_at	putative protein contains similarity to ethylene responsive element binding factor;supported by full-length cDNA: Ceres:2347.	0.05505	-0.50999	0.30904	3.23585	7.71E-18
946750	At3g50890	AtHB28	putative protein hypothetical protein T8K22.16 - Arabidopsis thaliana, chromosome II BAC T8K22, PIR2:T00609;supported by full-length cDNA: Ceres:20695.	0.09815	-0.50671	0.31138	3.21153	2.49E-05
954119	At1g29500	259773_at	auxin-induced protein, putative similar to SP:P33083 from [Glycine max]	0.03948	-0.50631	0.31167	3.20853	1.12E-34
957573	At1g10550	XTH33	putative endoxyloglucan transferase similar to xyloglucan endotransglycosylase-related protein XTR4 (pir|IS71223);supported by full-length cDNA: Ceres:27813.	0.09019	-0.50029	0.31602	3.16437	3.55E-06
948765	At4g24570	dic-02	putative mitochondrial uncoupling protein mitochondrial uncoupling protein, Arabidopsis thaliana (thale cress), PATX:E1316826;supported by full-length cDNA: Ceres:119476.	0.0431	-0.49673	0.31862	3.13856	6.70E-28
949357	At4g13790	254685_at	SAUR-AC - like protein (small auxin up RNA) SAUR-AC1, Arabidopsis thaliana, S70188;supported by full-length cDNA: Ceres:8965.	0.14274	-0.49446	0.32029	3.12218	0.024773
954130	At1g29450	259784_at	auxin-induced protein, putative similar to SP:P33083 from [Glycine max];supported by full-length cDNA: Ceres:29931.	0.06178	-0.49338	0.32109	3.11441	3.73E-13
941754	At5g66080	PP2C	protein phosphatase 2C-like protein	0.06526	-0.48886	0.32445	3.08216	1.55E-11
950307	At3g29575	AFP3	hypothetical protein	0.10265	-0.48778	0.32525	3.07453	1.78E-04
957650	At1g10560	PUB18	putative zinc-binding protein similar to zinc-binding protein (gi|3249068)	0.14271	-0.48175	0.3298	3.03215	0.033358
947147	At3g45960	ATEXLA3	putative protein cim1 induced allergen, Glycine max, EMBL:U03860	0.1317	-0.47169	0.33753	2.96269	0.017082
945190	At5g08130	BIM1	basic helix-loop-helix (bHLH) family protein involved in brassinosteroid signaling.	0.08343	-0.46947	0.33926	2.9476	2.33E-06
955188	At1g21910	260856_at	TINY-like protein similar to TINY GB:CAA64359 GI:1246403 from [Arabidopsis thaliana];supported by full-length cDNA: Ceres:19721.	0.05499	-0.46703	0.34117	2.93108	6.16E-15

959242	At1g61260	264879_at	cotton fiber expressed protein, putative similar to cotton fiber expressed protein 1 GI:3264828 from [Gossypium hirsutum]	0.11609	-0.46688	0.34129	2.9301	0.003611
942803	At5g54490	PBP1	putative protein similar to unknown protein (pir||T05752);supported by full-length cDNA: Ceres:109272.	0.05533	-0.45927	0.34732	2.8792	3.09E-14
961385	At2g31010	267201_at	putative protein kinase contains a protein kinase domain profile (PDOC00100)	0.11052	-0.45747	0.34876	2.86728	0.00195
960446	At2g18790	PHYB	phytochrome B Identical to GB:X17342	0.03904	-0.45629	0.34971	2.85949	1.12E-28
963364	At5g04190	PKS4	phytochrome kinase substrate 1 - like protein	0.10106	-0.44468	0.35919	2.78406	0.000711
942968	At5g52890	248278_at	putative protein similar to unknown protein (pir||T05472)	0.0994	-0.44363	0.36005	2.77737	6.25E-04
943065	At5g51670	248423_at	putative protein contains similarity to unknown protein (emb|CAB85560.1)	0.06277	-0.43633	0.36616	2.73107	6.92E-10
963768	At4g16770	245307_at	gibberellin oxidase-like protein non-consensus GG acceptor splice site at exon 8; supported by cDNA: gi_13265536_gb_AF324704.2_AF324704	0.06163	-0.4361	0.36635	2.72962	3.01E-10
949396	At4g13260	YUC2	putative protein dimethylaniline monooxygenase (N-oxide-forming) (EC1.14.13.8) -Oryctolagus cuniculus,PID:g164989	0.04834	-0.43376	0.36834	2.71492	1.06E-16
958412	At2g21200	264021_at	putative auxin-regulated protein ;supported by full-length cDNA: Ceres:7141.	0.08194	-0.42966	0.37183	2.6894	1.72E-05
961679	At2g45600	267503_at	unknown protein ; supported by full-length cDNA: Ceres:36855.	0.0709	-0.42142	0.37895	2.63889	4.04E-07
961160	At2g30040	MAPKKK14	putative protein kinase contains a protein kinase domain profile (PDOC00100)	0.04648	-0.41136	0.38783	2.57848	3.02E-16
955780	At1g21050	261456_at	hypothetical protein predicted by genemark.hmm;supported by full-length cDNA: Ceres:3200.	0.04522	-0.41057	0.38854	2.57376	4.11E-17
949514	At4g11280	ACS6	ACC synthase (AtACS-6) ; supported by cDNA: gi_16226285_gb_AF428292.1_AF428292	0.04059	-0.40541	0.39318	2.54336	8.77E-21
947577	At4g38850	SAUR15	small auxin up RNA (SAUR-AC1) ;supported by full-length cDNA: Ceres:14973.	0.08221	-0.40118	0.39703	2.51871	9.83E-05
950403	At4g10150	255802_s_at	putative protein RING-H2 finger protein RHA1a, Arabidopsis thaliana,AF078683	0.08946	-0.40034	0.3978	2.51383	5.95E-04
948567	At4g27260	WES1	GH3 like protein GH3 protein, Glycine max., PIR2:S17433	0.03234	-0.39787	0.40006	2.49961	8.11E-32
948728	At4g25260	254110_at	putative protein pectinesterase - Citrus sinensis, PID:g2098705	0.03382	-0.39736	0.40054	2.49664	5.51E-29
942240	At5g60840	247596_at	putative protein predicted protein, Drosophila melanogaster	0.08303	-0.39562	0.40215	2.48666	1.68E-04
958046	At1g04240	SHY2	putative auxin-induced protein AUX2-11 Match to Arabidopsis IAA3 (gb|U18406). EST gb|T04296 comes from this gene; supported by cDNA: gi_972910_gb_U18406.1_ATU18406	0.04451	-0.3918	0.4057	2.46488	4.60E-16
946167	At3g58640	251526_at	putative protein several serine/threonine-specific protein kinases	0.02412	-0.38668	0.41051	2.436	0
949688	At4g08950	EXO	putative phi-1-like phosphate-induced protein ;supported by full-length cDNA: Ceres:3552.	0.04643	-0.38189	0.41506	2.40927	5.63E-14

958603	At1g09250	264264_at	unknown protein ESTs gb|T04610, gb|N38459, gb|T45174, gb|R30481 and gb|N64971 come from this gene;supported by full-length cDNA: Ceres:9228.	0.03994	-0.38144	0.41549	2.40678	5.75E-19
952407	At3g19380	PUB25	hypothetical protein predicted genemark;supported by full-length cDNA: Ceres:255040.	0.05649	-0.37709	0.41967	2.38282	4.49E-09
942407	At5g59010	247743_at	protein kinase - like serine/threonine/tyrosine-specific protein kinase APK1, Arabidopsis thaliana, PIR:S28615	0.04233	-0.37599	0.42074	2.37679	2.33E-16
961537	At2g40000	HSPRO2	putative nematode-resistance protein ;supported by full-length cDNA: Ceres:35056.	0.0724	-0.37565	0.42106	2.37494	2.21E-05
945983	At3g60390	HAT3	homeobox-leucine zipper protein HAT3 ; supported by cDNA: gi_527632_gb_U09338.1_ATU09338	0.04969	-0.37444	0.42224	2.3683	1.11E-11
948625	At4g25810	XTR6	xyloglucan endo-1,4-beta-D-glucanase (XTR-6) ; supported by cDNA: gi_1244757_gb_U43488.1_ATU43488	0.06858	-0.37304	0.4236	2.36069	6.30E-06
948277	At4g29780	253643_at	hypothetical protein ;supported by full-length cDNA: Ceres:249769.	0.09252	-0.37166	0.42495	2.35322	0.003611
954136	At1g29430	259790_s_at	auxin-induced protein, putative similar to SP:P33083 from [Glycine max];supported by full-length cDNA: Ceres:4119.	0.04655	-0.36724	0.4293	2.32937	7.84E-13
943327	At5g48900	248681_at	pectate lyase non-consensus AG donor splice site at exon 2; supported by full-length cDNA: Ceres: 21311.	0.04772	-0.36217	0.43434	2.30234	7.60E-12
956885	At1g21820	262488_at	unknown protein EST gb|AA586241 comes from this gene	0.03451	-0.36102	0.4355	2.29624	7.54E-23
942241	At5g60860	AtRABA1f	GTP-binding protein - like GTP-binding protein, garden pea, PIR:T06447	0.0902	-0.35481	0.44176	2.26366	0.004655
960081	At2g31980	265672_at	putative cysteine proteinase inhibitor B (cystatin B) ;supported by full-length cDNA: Ceres:35447.	0.0762	-0.35377	0.44282	2.25823	2.88E-04
956017	At1g49780	PUB26	hypothetical protein predicted by genemark.hmm	0.05949	-0.35172	0.44492	2.24759	4.79E-07
946241	At3g58120	BZIP61	putative protein basic leucine zipper transcription activator shoot-forming PKSF1 - Paulownia kawakamii, EMBL:AF046934;supported by full-length cDNA: Ceres:34553.	0.04266	-0.35046	0.44621	2.24109	6.07E-14
947325	At3g44610	252646_at	protein kinase-like protein protein kinase - Solanum berthaultii, EMBL:X97980	0.05908	-0.34957	0.44712	2.23653	4.71E-07
958512	At1g78970	LUP1	lupeol synthase identical to lupeol synthase GI:1762150 from [Arabidopsis thaliana]; supported by cDNA: gi_15450512_gb_AY052358.1_	0.10447	-0.34847	0.44826	2.23085	0.03743
963920	At2g47440	245176_at	unknown protein similar to GP|2104534|AF001308 (T10M13.11)	0.03504	-0.34334	0.45358	2.20467	5.46E-20
957566	At1g54120	263151_at	hypothetical protein predicted by genemark.hmm;supported by full-length cDNA: Ceres:94743.	0.09787	-0.34289	0.45406	2.20235	0.021905
956638	At1g70940	PIN3	auxin transport protein REH1, putative similar to auxin transport protein REH1 GI:3377509 from [Oryza sativa]; supported by cDNA: gi_5817300_gb_AF087818.1_AF087818	0.03435	-0.34093	0.45611	2.19244	1.63E-20

953480	At3g02170	LNG2	unknown protein ;supported by full-length cDNA: Ceres:22225.	0.0318	-0.33634	0.46096	2.1694	2.31E-23
946869	At3g50060	MYB77	R2R3-MYB transcription factor ; supported by cDNA: gi_15983427_gb_AF424588.1_AF424588	0.05137	-0.3359	0.46142	2.16721	1.07E-08
946405	At3g55980	SZF1	putative protein zinc finger transcription factor (PEI1), Arabidopsis thaliana, EMBL:AF050463; supported by cDNA: gi_15810486_gb_AY056282.1_	0.04614	-0.32846	0.4694	2.13038	2.24E-10
960973	At2g46310	CRF5	putative AP2 domain transcription factor	0.06379	-0.32844	0.46942	2.13028	2.66E-05
961547	At2g39870	267339_at	unknown protein ; supported by cDNA: gi_13877742_gb_AF370134.1_AF370134	0.04338	-0.3237	0.47457	2.10716	1.88E-11
948056	At4g32350	253478_at	putative protein trichohyalin - human, PIR1:A45973	0.0551	-0.32265	0.47572	2.10207	6.70E-07
963750	At4g14130	XTR7	xyloglucan endotransglycosylase-related protein XTR-7 ;supported by full-length cDNA: Ceres:33554.	0.09547	-0.32184	0.47661	2.09817	0.03361
962572	At5g15830	AtbZIP3	bZIP DNA-binding protein-like putative bZIP DNA-binding protein - Capsicum chinense, EMBL:AF127797	0.05186	-0.31966	0.47901	2.08764	1.06E-07
946462	At3g54810	BME3	putative protein GATA transcription factor 3, Arabidopsis thaliana, Y13650; supported by cDNA: gi_15724333_gb_AF412107.1_AF412107	0.0327	-0.3179	0.48095	2.07921	1.14E-19
961657	At2g33810	SPL3	putative squamosa-promoter binding protein ;supported by full-length cDNA: Ceres:10375.	0.09339	-0.31769	0.48119	2.0782	0.030565
951954	At3g25710	BHLH32	putative HLH DNA-binding protein contains Pfam profile: PF00010 helix-loop-helix DNA-binding domain;supported by full-length cDNA: Ceres:247881.	0.03855	-0.31576	0.48333	2.06897	7.29E-14
948483	At4g28240	253812_at	putative wound induced protein wound-induced protein - tomato (fragment), PIR2:S19773;supported by full-length cDNA: Ceres:20161.	0.03858	-0.31033	0.48941	2.0433	2.38E-13
944588	At5g18930	BUD2	S-adenosyl-L-methionine decarboxylase - like protein S-adenosyl-L-methionine decarboxylase (SAMDC3), Brassica juncea, EMBL:AF215665	0.07798	-0.3089	0.49102	2.03658	0.004147
957387	At1g54200	263002_at	hypothetical protein predicted by genemark.hmm	0.04299	-0.30889	0.49103	2.03653	1.41E-10
942143	At5g61600	247543_at	DNA binding protein - like DNA binding protein EREBP-4, Nicotiana tabacum, PIR:T02434;supported by full-length cDNA: Ceres:92102.	0.0786	-0.3075	0.49261	2.03001	0.005184
951685	At3g19680	257076_at	unknown protein	0.04269	-0.30617	0.49412	2.0238	1.53E-10
942771	At5g55250	IAMT1	S-adenosyl-L-methionine:salicylic acid carboxyl methyltransferase-like protein ; supported by full-length cDNA: Ceres: 37493.	0.08349	-0.30607	0.49423	2.02334	0.013079
944776	At5g15160	250155_at	putative protein predicted proteins, Arabidopsis thaliana	0.06689	-0.3049	0.49557	2.01788	4.16E-04
945969	At3g61460	BRH1	RING finger protein ;supported by full-length cDNA: Ceres:25801.	0.02861	-0.30488	0.49559	2.01779	1.05E-23
963720	At4g17460	HAT1	homeobox-leucine zipper protein HAT1 (hd-zip protein 1) ;supported by full-length cDNA: Ceres:34167.	0.07411	-0.30441	0.49612	2.01564	0.002549

948948	At4g22780	ACR7	Translation factor EF-1 alpha - like protein translation factor EF-1 alpha genfamily, Arabidopsis thaliana, PATCHX:G1532164	0.08148	-0.3041	0.49648	2.01419	0.010319
947827	At4g34760	253255_at	putative auxin-regulated protein auxin-induced protein X15, Glycine max, PIR2:JQ1097;supported by full-length cDNA: Ceres:10510.	0.03673	-0.30068	0.50041	1.99838	7.54E-14
962639	At5g15580	LNG1	putative protein unknown protein F14P3.18 - Arabidopsis thaliana, EMBL:AC009755	0.03288	-0.30068	0.50041	1.99837	2.26E-17
963319	At1g73540	atnudt21	unknown protein contains similarity to diphosphoinositol polyphosphate phosphohydrolase GI:3978224 from [Homo sapiens]	0.04434	-0.29878	0.50259	1.98969	2.95E-09
947298	At3g44260	252679_at	CCR4-associated factor 1-like protein CAF1_MOUSE CCR4-ASSOCIATED FACTOR 1 - Mus musculus, SWISSPROT:CAF1_MOUSE; supported by cDNA: gi_15292828_gb_AY050848.1_	0.08119	-0.29835	0.50309	1.9877	0.012643
963801	At4g17490	ATERF6	ethylene responsive element binding factor-like protein (AtERF6) ; supported by cDNA: gi_3298497_dbj_AB013301.1_AB013301	0.05209	-0.29687	0.50481	1.98095	1.56E-06
944559	At5g22500	FAR1	male sterility 2-like protein (emb|CAA68191.1) ; supported by cDNA: gi_14334737_gb_AY035042.1_	0.06545	-0.29392	0.50825	1.96753	5.58E-04
956495	At1g59500	GH3.4	auxin-regulated protein GH3, putative similar to auxin-regulated protein GH3 GI:18590 from [Glycine max]	0.0562	-0.29294	0.5094	1.96311	1.96E-05
959156	At1g03610	264836_at	unknown protein similar to hypothetical protein GB:AAD11584;supported by full-length cDNA: Ceres:123030.	0.05432	-0.29052	0.51225	1.95217	1.02E-05
957228	At1g14920	GAI	signal response protein (GAI) identical to GAI GB:CAA75492 GI:2569938 [Arabidopsis thaliana] (Genes Dev. In press); supported by cDNA: gi_16648833_gb_AY058194.1_	0.02495	-0.28922	0.51379	1.94633	3.21E-28
952279	At3g17100	257894_at	unknown protein ; supported by cDNA: gi_15010765_gb_AY045684.1_	0.05426	-0.28915	0.51386	1.94605	1.13E-05
955662	At1g36940	261292_at	hypothetical protein predicted by genemark.hmm	0.06285	-0.28695	0.51648	1.9362	4.04E-04
963266	At1g32190	245784_at	hypothetical protein similar to hypothetical protein GB:AAD18105 GI:4337191 from [Arabidopsis thaliana]	0.03246	-0.28546	0.51825	1.92956	4.83E-16
952525	At3g24500	MBF1C	ethylene-responsive transcriptional coactivator, putative similar to GB:AAD46402 from [Lycopersicon esculentum] (Plant J. 18 (6), 589-600 (1999));supported by full-length cDNA: Ceres:158734.	0.0764	-0.2805	0.52421	1.90764	0.012643
945272	At5g06930	250653_at	putative protein strong similarity to unknown protein (gb|AAD23715.1)	0.04219	-0.27737	0.52799	1.89397	8.48E-09
948053	At4g32290	253475_at	putative protein predicted protein, Arabidopsis thaliana, PATCHX:G2252634	0.03543	-0.2766	0.52894	1.89059	1.46E-12
944270	At5g37770	TCH2	CALMODULIN-RELATED PROTEIN 2, TOUCH-INDUCED (TCH2) ;supported by full-length cDNA: Ceres:25475.	0.03016	-0.27594	0.52973	1.88774	2.20E-17
954129	At1g29510	SAUR68	auxin-induced protein, putative similar to SP:P33083 from [Glycine max];supported by full-length cDNA: Ceres:9311.	0.04754	-0.27311	0.5332	1.87548	1.24E-06

955401	At1g01260	261050_at	transcription factor MYC7E, putative similar to transcription factor MYC7E GB:AAD15818 GI:4321762 from [Zea mays]	0.06247	-0.27268	0.53373	1.87361	0.000711
943627	At5g45340	CYP707A3	cytochrome P450	0.06709	-0.27239	0.53409	1.87236	0.003082
943185	At5g49630	AAP6	amino acid permease 6 (emb CAA65051.1)	0.06698	-0.27234	0.53414	1.87216	0.003082
955286	At1g02640	BXL2	beta-xylosidase, putative similar to beta-xylosidase GB:Z84377 GI:2102655 from [Aspergillus niger]; supported by cDNA: gi_14194120_gb_AF367266.1_AF367266	0.07837	-0.27053	0.53638	1.86434	0.025963
951270	At3g28340	GATL10	unknown protein	0.06116	-0.26936	0.53782	1.85935	0.000711
945936	At3g62100	IAA30	auxin-induced protein homolog auxin-induced protein IAA20 - Arabidopsis thaliana, PIR:T02188	0.06218	-0.26766	0.53993	1.85208	0.001358
960718	At2g27080	266316_at	unknown protein ; supported by cDNA: gi_15450380_gb_AY052291.1_	0.051	-0.26739	0.54027	1.85092	1.72E-05
943175	At5g50570	248524_s_at	putative protein contains similarity to squamosa promoter binding protein;supported by full-length cDNA: Ceres:113229.	0.0412	-0.26569	0.54239	1.84368	1.85E-08
956886	At1g21830	262489_at	unknown protein EST gb|T21171 comes from this gene	0.03797	-0.26556	0.54255	1.84316	5.14E-10
958273	At2g36220	263931_at	unknown protein ;supported by full-length cDNA: Ceres:12251.	0.04469	-0.26425	0.54419	1.83761	4.78E-07
955579	At1g32920	261193_at	unknown protein ; supported by cDNA: gi_15450636_gb_AY052686.1_	0.047	-0.26376	0.5448	1.83553	2.49E-06
960806	At2g43290	MSS3	putative calcium binding protein ;supported by full-length cDNA: Ceres:31535.	0.02529	-0.25972	0.5499	1.81851	5.29E-22
962317	At4g36780	246284_at	putative protein	0.0484	-0.25635	0.55418	1.80447	1.33E-05
949665	At4g09890	PMP	putative protein Arabidopsis thaliana chromosome II BAC T30B22 genomic sequence, gene T30B22.22, PID:g2529679	0.02715	-0.25537	0.55543	1.8004	2.12E-18
943481	At5g47380	248784_at	putative protein similar to unknown protein (pir||T02421)	0.04727	-0.25481	0.55615	1.79809	8.20E-06
959567	At2g43060	265245_at	unknown protein	0.06364	-0.25413	0.55702	1.79528	0.004147
961495	At2g23760	BLH4	putative homeodomain transcription factor ; supported by cDNA: gi_13877512_gb_AF353092.1_AF353092	0.03847	-0.25169	0.56016	1.78521	1.04E-08
954598	At1g68550	CPuORF53	putative AP2 domain transcription factor contains Pfam profile: PF00847 AP2 domain; supported by cDNA: gi_16604674_gb_AY059782.1_	0.02948	-0.25167	0.56019	1.78512	4.47E-15
948434	At4g28640	IAA11	early auxin-inducible protein 11 (IAA11) ; supported by cDNA: gi_972924_gb_U18413.1_ATU18413	0.05289	-0.25164	0.56023	1.78499	1.73E-04
954359	At1g76600	259979_at	unknown protein ;supported by full-length cDNA: Ceres:29624.	0.04834	-0.24979	0.56261	1.77742	2.45E-05
941868	At5g65140	247228_at	trehalose-6-phosphate phosphatase	0.06985	-0.24906	0.56355	1.77445	0.017968
942391	At5g58650	PSY1	putative protein ; supported by cDNA: gi_15529263_gb_AY052256.1_	0.05269	-0.24756	0.56551	1.76831	2.26E-04
942802	At5g54510	DFL1	auxin-responsive-like protein ; supported by cDNA: gi_11041725_dbj_AB050596.1_AB050596	0.01922	-0.24684	0.56645	1.76538	9.16E-35

951749	At3g15210	ERF4	ethylene responsive element binding factor 4 (AtERF4) identical to GB:BAA32421 from [Arabidopsis thaliana];supported by full-length cDNA: Ceres:22775.	0.03624	-0.24666	0.56669	1.76464	1.88E-09
953424	At3g04860	259093_at	unknown protein similar to putative protein GB:CAB40986 [Arabidopsis thaliana];supported by full-length cDNA: Ceres:5170.	0.05318	-0.24507	0.56876	1.75821	3.34E-04
957805	At2g17230	EXL5	unknown protein ;supported by full-length cDNA: Ceres:641.	0.05314	-0.24443	0.5696	1.75562	3.46E-04
955034	At1g19380	260656_at	hypothetical protein predicted by genemark.hmm	0.06049	-0.24427	0.56981	1.75498	0.003082
943790	At5g43700	ATAUX2-11	auxin-induced protein AUX2-11 (sp P33077)	0.0324	-0.24424	0.56984	1.75487	1.09E-11
951962	At3g16800	257650_at	protein phosphatase, putative similar to protein phosphatase-2C GB:AAC36699 from [Mesembryanthemum crystallinum];supported by full-length cDNA: Ceres:6305.	0.05707	-0.24352	0.57079	1.75195	0.001358
948204	At4g31000	253571_at	putative calmodulin-binding protein calmodulin-binding protein, Nicotiana tabacum	0.05739	-0.24256	0.57206	1.74806	0.001358
946283	At3g57795	251601_at	Expressed protein ; supported by full-length cDNA: Ceres: 19033.	0.04633	-0.24094	0.5742	1.74157	2.08E-05
956372	At1g64640	261975_at	unknown protein	0.03862	-0.23888	0.57693	1.73331	9.46E-08
953627	At3g11490	259287_at	putative rac GTPase activating protein similar to rac GTPase activating protein 1 GB:AAC62624 [Lotus japonicus]	0.04666	-0.23745	0.57883	1.72762	3.61E-05
943231	At5g50130	248539_at	ribitol dehydrogenase-like ; supported by cDNA: gi_15146201_gb_AY049242.1_	0.06973	-0.23741	0.57888	1.72747	0.030352
947535	At4g39400	BRI1	brassinosteroid insensitive 1 gene (BRI1)	0.03324	-0.235	0.5821	1.71791	3.10E-10
945836	At3g62720	XT1	alpha galactosyltransferase-like protein alpha galactosyltransferase - Trigonella foenum-graecum, EMBL:TFO245478; supported by cDNA: gi_15983425_gb_AF424587.1_AF424587	0.03923	-0.23387	0.58361	1.71346	3.66E-07
961613	At2g44500	267393_at	similar to axi 1 protein from Nicotiana tabacum	0.02771	-0.23299	0.5848	1.70999	1.27E-14
946377	At3g55740	PROT2	proline transporter 2	0.02857	-0.23062	0.588	1.70067	1.88E-13
954001	At1g69010	BIM2	putative DNA-binding protein predicted by genscan, multiple est matches; Pfam HMM hit: helix-loop-helix DNA-binding domain;supported by full-length cDNA: Ceres:27793.	0.05663	-0.23053	0.58813	1.70031	0.003082
944041	At5g40540	249361_at	protein kinase - like protein protein kinase ATN1, Arabidopsis thaliana, PIR:S61766	0.05742	-0.22939	0.58967	1.69586	0.003611
955859	At1g21060	261451_at	unknown protein	0.06429	-0.2279	0.5917	1.69003	0.019172
958670	At1g78700	264248_at	hypothetical protein predicted by genscan+; supported by cDNA: gi_15215801_gb_AY050430.1_	0.06538	-0.22698	0.59295	1.68648	0.024356
942087	At5g62020	HSFB2A	heat shock factor 6	0.05534	-0.2252	0.59539	1.67957	0.003082
951680	At3g28180	ATCSLC04	unknown protein ; supported by cDNA: gi_15810494_gb_AY056286.1_	0.02853	-0.22291	0.59853	1.67075	1.39E-12
946500	At3g54200	251879_at	putative protein hin1 protein, Nicotiana tabacum, PIR:T03265;supported by full-length cDNA: Ceres:1678.	0.03965	-0.22275	0.59876	1.67013	2.46E-06

950055	At4g03190	GRH1	F-box protein GRR1-like protein 1, AtFBL18 almost identical to GRR1-like protein 1 GI:12658970 from [Arabidopsis thaliana]	0.02126	-0.2217	0.6002	1.66611	1.04E-22
946660	At3g53180	251973_at	nodulin / glutamate-ammonia ligase - like protein MtN6 - nodulin 6, Medicago truncatula, EMBL:MET133118	0.0364	-0.2205	0.60187	1.66148	2.06E-07
953043	At3g08640	258692_at	unknown protein	0.02751	-0.21911	0.6038	1.65619	4.33E-13
947805	At4g35470	253136_at	putative protein leucine-rich protein - Helianthus annuus (common sunflower),PIR:T12704	0.04525	-0.21803	0.60529	1.65209	1.32E-04
945912	At3g61830	ARF18	auxin response factor-like protein auxin response factor 9 - Arabidopsis thaliana, PIR:T08917; supported by cDNA: gi_16604602_gb_AY059746.1_	0.04679	-0.21683	0.60697	1.64752	2.97E-04
944484	At5g22940	F8H	putative protein strong similarity to unknown protein (gb|AAC98455.1)	0.0538	-0.21658	0.60733	1.64655	0.003611
946152	At3g59350	251494_at	protein kinase-like protein Pto kinase interactor 1 - Lycopersicon esculentum, EMBL:U28007; supported by cDNA: gi_15451117_gb_AY054639.1_	0.03391	-0.21563	0.60865	1.64297	3.23E-08
954610	At1g74670	260221_at	GAST1-like protein similar to GAST1 protein precursor GB:P27057 [Lycopersicon esculentum] (induced by gibberellins, inhibited by ABA Plant J 1992 Mar;2(2):153-9)	0.04036	-0.21446	0.61029	1.63856	1.23E-05
952545	At3g13980	258196_at	hypothetical protein predicted by genemark.hmm	0.04815	-0.21444	0.61032	1.63849	6.51E-04
960949	At2g14900	266613_at	similar to gibberellin-regulated proteins	0.05867	-0.21406	0.61086	1.63704	0.01354
948101	At4g31820	ENP	putative protein various predicted proteins	0.0528	-0.21322	0.61205	1.63387	0.003082
941836	At5g64780	247255_at	putative protein similar to unknown protein (pir||T04031); supported by full-length cDNA: Ceres: 144066.	0.06483	-0.21164	0.61427	1.62795	0.046724
947483	At4g39800	MIPS1	myo-inositol-1-phosphate synthase ;supported by full-length cDNA: Ceres:1978.	0.03174	-0.21008	0.61649	1.62209	6.42E-09
958875	At1g09570	PHYA	putative phytochrome A similar to GB:AAA21351; supported by cDNA: gi_14517371_gb_AY039520.1_	0.04792	-0.20978	0.61691	1.62098	0.000711
955218	At1g06850	AtbZIP52	b-Zip DNA binding protein, putative similar to b-Zip DNA binding protein GB:CAB06697 GI:2246376 from [Arabidopsis thaliana]	0.04443	-0.20943	0.61741	1.61968	2.13E-04
958288	At2g36400	AtGRF3	unknown protein	0.04877	-0.20772	0.61984	1.61331	0.001358
942245	At5g60850	OBP4	zinc finger protein OBP4 - like zinc finger protein OBP4, Arabidopsis thaliana, EMBL:AF155817;supported by full-length cDNA: Ceres:19080.	0.05362	-0.20716	0.62064	1.61123	0.006273
953484	At3g02140	TMAC2	hypothetical protein predicted by genscan; supported by cDNA: gi_14334515_gb_AY034949.1_	0.05803	-0.20576	0.62265	1.60605	0.019172
952654	At3g15770	258262_at	hypothetical protein	0.04193	-0.20547	0.62307	1.60496	9.04E-05
943196	At5g49700	248564_at	putative protein contains similarity to AT-hook DNA-binding protein	0.04041	-0.20511	0.62358	1.60364	3.82E-05
958782	At1g11960	264389_at	unknown protein similar to hypothetical protein HYP1 gb|Z97338 from A. thaliana	0.04929	-0.20428	0.62477	1.60058	0.00195

953498	At3g01490	259163_at	putative protein kinase similar to ATMRK1, an Arabidopsis protein kinase related to mammal mixed-lineage kinases and Raf protein kinases GB:BAA22079 [Arabidopsis thaliana]; supported by cDNA: gi_16323086_gb_AY057647.1_	0.04981	-0.20306	0.62653	1.59609	0.003082
954685	At1g80280	260297_at	unknown protein contains Pfam profile: PF00561 alpha/beta hydrolase fold	0.02754	-0.20212	0.62788	1.59267	4.56E-11
958527	At1g65310	XTH17	xyloglucan endotransglycosylase, putative similar to xyloglucan endotransglycosylase 1 GI:3901012 from [Fagus sylvatica]	0.04653	-0.20185	0.62828	1.59165	0.000711
958308	At2g21050	264025_at	AUX1-like amino acid permease ; supported by cDNA: gi_15451207_gb_AY054684.1_	0.03715	-0.20101	0.62949	1.58858	7.31E-06
954905	At2g41820	260494_at	putative receptor-like protein kinase	0.05111	-0.20085	0.62973	1.58799	0.005184
942229	At5g60680	247585_at	putative protein predicted proteins, Arabidopsis thaliana;supported by full-length cDNA: Ceres:16638.	0.03353	-0.20038	0.6304	1.58629	3.36E-07
962099	At5g10695	246018_at	Expressed protein ; supported by full-length cDNA: Ceres: 103171.	0.03985	-0.19962	0.63151	1.58349	5.34E-05
963940	At2g45210	245136_at	putative auxin-regulated protein	0.05036	-0.19926	0.63203	1.58221	0.004655
950269	At4g00820	iqd17	hypothetical protein	0.03991	-0.19925	0.63205	1.58216	5.79E-05
946262	At3g57450	251640_at	putative protein ;supported by full-length cDNA: Ceres:12522.	0.03679	-0.19494	0.63836	1.56652	1.32E-05
960126	At2g01150	RHA2B	RING-H2 finger protein RHA2b identical to GP|AF078823; supported by cDNA: gi_3790570_gb_AF078823.1_AF078823	0.04163	-0.19458	0.63888	1.56525	2.53E-04
953752	At1g13260	RAV1	DNA-binding protein RAV1 identical to RAV1 GI:3868857 from [Arabidopsis thaliana]; supported by cDNA: gi_3868856_dbj_AB013886.1_AB013886	0.03689	-0.1942	0.63944	1.56386	1.56E-05
961280	At2g32560	267116_at	unknown protein	0.05235	-0.19384	0.63997	1.56258	0.011271
960621	At2g46660	CYP78A6	putative cytochrome P450	0.04607	-0.19245	0.64202	1.55759	0.00195
953164	At3g04640	258792_at	hypothetical protein predicted by genefinder;supported by full-length cDNA: Ceres:8992.	0.05364	-0.18853	0.64784	1.5436	0.021129
950547	At1g22190	255926_at	AP2 domain containing protein RAP2, putative similar to AP2 domain containing protein RAP2.4 GI:2281633 from [Arabidopsis thaliana]; supported by cDNA: gi_15292782_gb_AY050825.1_	0.05036	-0.18815	0.64841	1.54223	0.010319
956582	At1g68560	XYL1	alpha-xylosidase precursor identical to alpha-xylosidase precursor GB:AAD05539 GI:4163997 from [Arabidopsis thaliana]; supported by cDNA: gi_15982750_gb_AY057482.1_	0.0251	-0.18794	0.64872	1.5415	1.56E-11
945894	At3g62660	GATL7	putative protein glycosyl transferase lgtC - Neisseria gonorrhoeae, EMBL:AF208062	0.05252	-0.18618	0.65136	1.53526	0.019172
942614	At5g57100	247944_at	putative protein similar to unknown protein (gb|AAF04433.1);supported by full-length cDNA: Ceres:156439.	0.04718	-0.18599	0.65164	1.5346	0.004655
943738	At5g44250	249064_at	putative protein contains similarity to unknown protein (gb|AAD17413.1);supported by full-length cDNA: Ceres:27195.	0.04153	-0.18586	0.65185	1.53411	5.95E-04

950629	At1g58340	ZF14	unknown protein contains Pfam profile: PF01554 uncharacterized membrane protein family UPF0013; supported by cDNA: gi_6520160_dbj_AB028198.1_AB028198	0.04402	-0.18506	0.65304	1.53131	0.00195
942108	At5g62220	247470_at	putative protein various predicted proteins, Arabidopsis thaliana	0.03799	-0.18479	0.65345	1.53034	1.06E-04
944619	At5g19120	249923_at	conglutin gamma - like protein conglutin gamma precursor, Lupinus angustifolius, PIR:S21426; supported by cDNA: gi_15010797_gb_AY045700.1_	0.05643	-0.18446	0.65394	1.52919	0.046219
946165	At3g58620	TTL4	putative protein At2g42580 - Arabidopsis thaliana, EMBL:AC007087	0.03975	-0.18028	0.66026	1.51455	4.59E-04
942603	At5g56980	247933_at	putative protein non-consensus CG donor splice site at exon 1, GA donor splice site at exon 3, similar to unknown protein (pir||T04268);supported by full-length cDNA: Ceres:32257.	0.04376	-0.17961	0.66128	1.51222	0.002549
948150	At4g31390	253517_at	predicted protein hypothetical protein slr1919, Synechocystis sp., PIR2:S75233	0.04301	-0.17916	0.66197	1.51065	0.00195
952225	At3g12920	257858_at	hypothetical protein predicted by genefinder;supported by full-length cDNA: Ceres:924.	0.03524	-0.17887	0.66242	1.50961	3.82E-05
947223	At3g45780	PHOT1	nonphototropic hypocotyl 1 ; supported by cDNA: gi_14532875_gb_AY040062.1_	0.0276	-0.17867	0.66272	1.50892	1.58E-08
954470	At1g78100	260058_at	unknown protein ; supported by cDNA: gi_15450975_gb_AY054568.1_	0.03554	-0.17724	0.66491	1.50397	5.92E-05

Table S3. List of shade- regulated and IAA-, BL- and GA-regulated genes.

Class SD and SU		
**Class SD, Down-regulated**		
**Accession #**	**Sequence Description**	**Primary Gene Symbol**
At3g44450	Unknown protein.	-
At1g70560	TAA1/SAV3 is involved in the shade-induced production of indole-3-pyruvate (IPA), a precursor to IAA, a biologically active auxin. It is also involved in regulating many aspects of plant growth and development from embryogenesis to flower formation and plays a role in ethylene-mediated signaling. This enzyme can catalyze the formation of IPA from L-tryptophan. Though L-Trp is expected to be the preferred substrate in vivo, TAA1 also acts as an aminotransferase using L-Phe, L-Tyr, L-Leu, L-Ala, L-Met, and L-Gln.	TRYPTOPHAN AMINOTRANSFERASE OF ARABIDOPSIS 1 (TAA1)
At5g41410	Homeodomain protein required for ovule identity.Loss of function mutations show homeotic conversion of integuments to carpels.Forms heterodimers with STM and KNAT1. Interacts with AG-SEP heterodimers is thought to restrict WUS expression. BEL interacts with MADS box dimers composed of SEP1(or SEP3) and AG, SHP1, SHP2 and STK. The interaction of BEL1 with AG-SEP3 is required for proper integument development and specification of integument identity.	BELL 1 (BEL1)
At3g47420	Encodes a Pi starvation-responsive protein AtPS3. A member of the phosphate starvation-induced glycerol-3-phosphate permease gene family.	GLYCEROL-3-PHOSPHATE PERMEASE 1 (G3Pp1)
At1g51805	Leucine-rich repeat protein kinase family protein.	
At1g74940	Protein of unknown function (DUF581).	
At3g44990	xyloglucan endo-transglycosylase	XYLOGLUCAN ENDO-TRANSGLYCOSYLASE-RELATED 8 (XTR8)
At2g15050	Predicted to encode a PR (pathogenesis-related) protein. Belongs to the lipid transfer protein (LTP) family.	LIPID TRANSFER PROTEIN 7 (LTP7)
At4g24780	Pectin lyase-like superfamily protein.	
At4g21990	Encodes a protein disulfide isomerase-like (PDIL) protein, a member of a multigene family within the thioredoxin (TRX) superfamily. This protein also belongs to the adenosine 5'-phosphosulfate reductase-like (APRL) group.	APS REDUCTASE 3 (APR3)
At1g75710	C2H2-like zinc finger protein.	
**Class SU, Up-regulated**		
**Accession #**	**Sequence Description**	**Primary Gene Symbol**
At1g68550	Encodes a member of the ERF (ethylene response factor) subfamily B-6 of ERF/AP2 transcription factor family.	CYTOKININ RESPONSE FACTOR 10 (CRF10)
At3g58620	Encodes one of the 36 carboxylate clamp (CC)-tetratricopeptide repeat (TPR) proteins.	TETRATRICOPETIDE-REPEAT THIOREDOXIN-LIKE 4 (TTL4)
At3g13980	Unknown protein.	-
At5g06930	Unknown function.	-
At5g48900	Pectin lyase-like superfamily protein.	-

At3g57795	-	-
At4g32290	Core-2/I-branching beta-1,6-N-acetylglucosaminyltransferase family protein.	-
At3g02170	Encodes LONGIFOLIA2 (LNG2). Regulates leaf morphology by promoting cell expansion in the leaf-length direction. The LNG2 homologue LNG1 (At5g15580) has similar function.	LONGIFOLIA2 (LNG2)
At4g28240	Wound-responsive family protein.	-
At3g17100	Sequence-specific DNA binding transcription factors.	-
At3g54810	Encodes a protein containing a GATA type zinc finger domain that is expressed in the embryo axis and involved in germination.	BLUE MICROPYLAR END 3 (BME3)/GATA TRANSCRIPTION FACTOR 8 (GATA8)
At2g47440	Tetratricopeptide repeat (TPR)-like superfamily protein.	-
At1g32920	Unknown protein.	-
At1g54200	Unknown protein.	-
At1g04240	SHY2/IAA3 regulates multiple auxin responses in roots. It is induced rapidly by IAA, and has been shown to be phosphorylated by oat phytochrome A in vitro.	SHORT HYPOCOTYL 2 (SHY2)
At1g14920	Repressor of GA responses and involved in gibberellic acid mediated signaling.	GIBBERELLIC ACID INSENSITIVE (GAI)
At1g70940	A regulator of auxin efflux and involved in differential growth.	PIN-FORMED 3 (PIN3)
At2g46310	CRF5 encodes one of the six cytokinin response factors.	CYTOKININ RESPONSE FACTOR 5 (CRF5)
At3g58640	Mitogen activated protein kinase kinase kinase-related.	-
At4g34760	SAUR-like auxin-responsive protein family.	-
At1g29510	SAUR-like auxin-responsive protein family.	SMALL AUXIN UPREGULATED 68 (SAUR68);
At1g21820	-	-
At2g40000	Ortholog of sugar beet HS1 PRO-1 2 (HSPRO2).	ORTHOLOG OF SUGAR BEET HS1 PRO-1 2 (HSPRO2)
At4g27260	Encodes an IAA-amido synthase that conjugates Asp and other amino acids to auxin in vitro.	(WES1)
At1g01260	Basic helix-loop-helix (bHLH) DNA-binding superfamily protein.	-
At4g08950	Unknown function.	EXORDIUM (EXO)
At2g39870	Unknown protein.	-
At3g19680	Protein of unknown function.	-
At4g16770	2-oxoglutarate (2OG) and Fe(II)-dependent oxygenase superfamily protein.	-
At3g58120	Encodes a member of the BZIP family of transcription factors.	(BZIP61)
At4g11280	Encodes a member of the 1-aminocyclopropane-1-carboxylate (ACC) synthase gene family.	1-AMINOCYCLOPROPANE-1-CARBOXYLIC ACID (ACC) SYNTHASE 6 (ACS6)
At4g25260	Plant invertase/pectin methylesterase inhibitor superfamily protein.	-
At1g09250	Basic helix-loop-helix (bHLH) DNA-binding superfamily protein.	-

At1g21910	Encodes a member of the DREB subfamily A-5 of ERF/AP2 transcription factor family.	DEHYDRATION RESPONSE ELEMENT-BINDING PROTEIN 26 (DREB26)
At1g29430	SAUR-like auxin-responsive protein family.	-
At4g13260	Encodes YUC2. Catalyzes conversion of IPA (indole-3-pyruvic acid) to IAA (indole-3-acetic acid) in auxin biosynthesis pathway.	YUCCA2 (YUC2)
At4g16515	Encodes a root meristem growth factor (RGF).	ROOT MERISTEM GROWTH FACTOR 6 (RGF6)
At2g18790	Red/far-red photoreceptor involved in the regulation of de-etiolation.	PHYTOCHROME B (PHYB)
At5g08130	Arabidopsis thaliana basic helix-loop-helix (bHLH) family protein involved in brassinosteroid signaling.	(BIM1)
At4g38400	Member of EXPANSIN-LIKE.	EXPANSIN-LIKE A2 (EXLA2)
At1g29450	SAUR-like auxin-responsive protein family.	-
At1g29500	SAUR-like auxin-responsive protein family.	-
At1g21050	Protein of unknown function.	-
At1g02660	Alpha/beta-Hydrolases superfamily protein.	-
At4g01250	AtWRKY22 is a member of WRKY Transcription Factor.	(WRKY22)
At3g23030	Auxin inducible gene expressed in the nucleus.	INDOLE-3-ACETIC ACID INDUCIBLE 2 (IAA2)
At3g29575	ABI five binding protein 3 (AFP3).	ABI FIVE BINDING PROTEIN 3 (AFP3)
At5g07000	Encodes a member of the sulfotransferase family of proteins.	SULFOTRANSFERASE 2B (ST2B)
At1g29460	SAUR-like auxin-responsive protein family.	-
At1g29440	SAUR-like auxin-responsive protein family.	SMALL AUXIN UP RNA 63 (SAUR63)
At5g46240	Encodes a potassium channel protein (KAT1).	POTASSIUM CHANNEL IN ARABIDOPSIS THALIANA 1 (KAT1)
At5g51190	Encodes a member of the ERF (ethylene response factor) subfamily B-3 of ERF/AP2 transcription factor family.	-
At4g34770	SAUR-like auxin-responsive protein family.	-
At2g42380	Encodes a member of the BZIP family of transcription factors.	Encodes a member of the BZIP family of transcription factors
At5g25190	Encodes a member of the ERF (ethylene response factor) subfamily B-6 of ERF/AP2 transcription factor family.	ETHYLENE AND SALT INDUCIBLE 3 (ESE3)
At5g12050	Unknown protein.	-
At5g66590	CAP (Cysteine-rich secretory proteins, Antigen 5, and Pathogenesis-related 1 protein) superfamily protein.	-
At3g50340	Unknown protein.	-
At5g44260	Zinc finger C-x8-C-x5-C-x3-H type family protein.	-
At5g62280	Protein of unknown function.	-
At3g03840	SAUR-like auxin-responsive protein family.	SMALL AUXIN UP RNA 27 (SAUR27)

At5g02760	Protein phosphatase 2C family protein.	-
At5g43890	Encodes a YUCCA-like putative flavin monooxygenase.	YUCCA 5 (YUC5)
At4g27280	Calcium-binding EF-hand family protein.	-
At5g52900	Encodes a member of the MAKR (MEMBRANE-ASSOCIATED KINASE REGULATOR) gene family.	MEMBRANE-ASSOCIATED KINASE REGULATOR 6 (MAKR6)
At5g18060	SAUR-like auxin-responsive protein family.	SMALL AUXIN UP RNA 23 (SAUR23)
At5g47370	Homeobox-leucine zipper genes induced by auxin, but not by other phytohormones.	HAT2
At5g57560	Encodes a cell wall-modifying enzyme.	TOUCH 4 (TCH4)
At1g35140	Involved in the C-starvation response.	PHOSPHATE-INDUCED 1 (PHI-1)
At2g44910	Encodes a homeodomain protein whose expression displays a dependence on phyB for both red and far-red light response. Also involved in the shade avoidance syndrome.	ARABIDOPSIS THALIANA HOMEOBOX-LEUCINE ZIPPER PROTEIN 4 (ATHB-4)
At1g02340	Encodes a light-inducible, nuclear bHLH protein involved in phytochrome signaling.	LONG HYPOCOTYL IN FAR-RED (HFR1)
At4g28720	Auxin biosynthetic gene.	YUCCA 8 (YUC8)
At1g75450	CKX5 (it used to be called AtCKX6) encodes a protein whose sequence is similar to cytokinin oxidase/dehydrogenase, which catalyzes the degradation of cytokinins.	CYTOKININ OXIDASE 5 (CKX5)
At3g15540	Primary auxin-responsive gene.	INDOLE-3-ACETIC ACID INDUCIBLE 19 (IAA19)
At3g03830	SAUR-like auxin-responsive protein family.	SMALL AUXIN UP RNA 28 (SAUR28)
At5g07010	Encodes a sulfotransferase that acts specifically on 11- and 12-hydroxyjasmonic acid.	SULFOTRANSFERASE 2A (ST2A)
At4g32280	Auxin inducible protein.	INDOLE-3-ACETIC ACID INDUCIBLE 29 (IAA29)
At2g42870	Encodes PHYTOCHROME RAPIDLY REGULATED1 (PAR1), an atypical basic helix-loop-helix (bHLP) protein.	PHY RAPIDLY REGULATED 1 (PAR1)
At4g16780	Encodes a homeodomain-leucine zipper protein that is rapidly and strongly induced by changes in the ratio of red to far-red light.	ARABIDOPSIS THALIANA HOMEOBOX-LEUCINE ZIPPER PROTEIN 4 (ATHB-4) / HOMEOBOX-LEUCINE ZIPPER PROTEIN 4 (HB4)
At1g04180	Auxin biosynthetic gene.	YUCCA 9 (YUC9)
At4g37770	Encodes an auxin inducible ACC synthase.	1-AMINO-CYCLOPROPANE-1-CARBOXYLATE SYNTHASE 8 (ACS8)

Class S+A, shade-regulated genes (in both Col-0 and *sav3* seedlings) and IAA-regulated genes.

**Shade down-regulated + IAA up-regulated**		
**Accession #**	**Sequence Description**	**Primary Gene Symbol**
**Shade down-regulated + IAA down-regulated**		
**Accession #**	**Sequence Description**	**Primary Gene Symbol**
At2g15050	Predicted to encode a PR (pathogenesis-related) protein. Belongs to the lipid transfer protein (PR-14) family with the following members: At2g38540/LTP1, At2g38530/LTP2, At5g59320/LTP3, At5g59310/LTP4, At3g51600/LTP5, At3g08770/LTP6, At2g15050/LTP7, At2g18370/LTP8, At2g15325/LTP9, At5g01870/LTP10, At4g33355/LTP11, At3g51590/LTP12, At5g44265/LTP13, At5g62065/LTP14, At4g08530/LTP15.	LTP7
**Shade up-regulated + IAA up-regulated**		
**Accession #**	**Sequence Description**	Primary Gene Symbol
At5g06930	Unknown protein.	-
At3g02170	Encodes LONGIFOLIA2 (LNG2). Regulates leaf morphology by promoting cell expansion in the leaf-length direction. The LNG2 homologue LNG1 (At5g15580) has similar function.	LONGIFOLIA2 (LNG2)
At1g32920	Unknown protein.	-
At1g54200	Unknown protein.	-
At1g04240	SHY2/IAA3 regulates multiple auxin responses in roots. It is induced rapidly by IAA, and has been shown to be phosphorylated by oat phytochrome A in vitro.	SHORT HYPOCOTYL 2 (SHY2)
At1g70940	A regulator of auxin efflux and involved in differential growth.	PIN-FORMED 3 (PIN3)
At4g34760	SAUR-like auxin-responsive protein family.	-
At1g29510	SAUR-like auxin-responsive protein family.	SMALL AUXIN UPREGULATED 68 (SAUR68);
At1g21820	-	-
At4g27260	Encodes an IAA-amido synthase that conjugates Asp and other amino acids to auxin in vitro. Lines carrying insertions in this gene are hypersensitive to auxin. It is involved in camalexin biosynthesis via conjugating indole-3-carboxylic acid (ICA) and cysteine (Cys).	WES1
At4g08950	Unknown function.	EXORDIUM (EXO)
At2g39870	Unknown protein.	-
At3g58120	Encodes a member of the BZIP family of transcription factors.	BZIP61
At4g11280	Encodes a member of the 1-aminocyclopropane-1-carboxylate (ACC) synthase (S-adenosyl-L-methionine methylthioadenosine-lyase, EC 4.4.1.14) gene family.	1-AMINOCYCLOPROPANE-1-CARBOXYLIC ACID (ACC) SYNTHASE 6 (ACS6)
At1g09250	basic helix-loop-helix (bHLH) DNA-binding superfamily protein.	-
At1g29430	SAUR-like auxin-responsive protein family.	-
At4g16515	Encodes a root meristem growth factor (RGF).	ROOT MERISTEM GROWTH FACTOR 6 (RGF6)
At1g29450	SAUR-like auxin-responsive protein family.	-

At1g29500	SAUR-like auxin-responsive protein family.	-
At1g21050	Protein of unknown function.	-
At1g02660	Alpha/beta-Hydrolases superfamily protein.	-
At3g23030	Auxin inducible gene.	INDOLE-3-ACETIC ACID INDUCIBLE 2 (IAA2)
At1g29460	SAUR-like auxin-responsive protein family.	-
At1g29440	SAUR-like auxin-responsive protein family.	SMALL AUXIN UP RNA 63 (SAUR63)
At5g51190	Encodes a member of the ERF (ethylene response factor) subfamily B-3 of ERF/AP2 transcription factor family.	-
At4g34770	SAUR-like auxin-responsive protein family.	-
At5g25190	Encodes a member of the ERF (ethylene response factor) subfamily B-6 of ERF/AP2 transcription factor family.	ETHYLENE AND SALT INDUCIBLE 3 (ESE3)
At5g12050	Unknown protein.	-
At3g50340	Unknown protein.	-
At5g44260	Zinc finger C-x8-C-x5-C-x3-H type family protein.	-
At5g62280	Protein of unknown function.	-
At3g03840	SAUR-like auxin-responsive protein family.	SMALL AUXIN UP RNA 27 (SAUR27)
At5g02760	Protein phosphatase 2C family protein.	-
At4g27280	Calcium-binding EF-hand family protein.	-
At5g52900	Encodes a member of the MAKR (MEMBRANE-ASSOCIATED KINASE REGULATOR) gene family.	MEMBRANE-ASSOCIATED KINASE REGULATOR 6 (MAKR6)
At5g18060	SAUR-like auxin-responsive protein family.	SMALL AUXIN UP RNA 23 (SAUR23)
At5g47370	homeobox-leucine zipper genes induced by auxin, but not by other phytohormones.	HAT2
At5g57560	Encodes a cell wall-modifying enzyme.	TOUCH 4 (TCH4)
At1g35140	Involved in the C-starvation response.	PHOSPHATE-INDUCED 1 (PHI-1)
At3g15540	Primary auxin-responsive gene.	INDOLE-3-ACETIC ACID INDUCIBLE 19 (IAA19)
At3g03830	SAUR-like auxin-responsive protein family.	SMALL AUXIN UP RNA 28 (SAUR28)
At4g32280	Auxin inducible protein.	INDOLE-3-ACETIC ACID INDUCIBLE 29 (IAA29)
At4g37770	Encodes an auxin inducible ACC synthase.	1-AMINO-CYCLOPROPANE-1-CARBOXYLATE SYNTHASE 8 (ACS8)
**Shade up-regulated + IAA down-regulated**		
**Accession #**	**Sequence Description**	**Primary Gene Symbol**

Class S+B, shade-regulated genes (in both Col-0 and *sav3* seedlings) and BL-regulated genes.

Shade down-regulated + BL up-regulated

Accession #	Sequence Description	Primary Gene Symbol

Shade down-regulated + BL down-regulated

Accession #	Sequence Description	Primary Gene Symbol

Shade up-regulated + BL up-regulated

Accession #	Sequence Description	Primary Gene Symbol
At3g58620	Encodes one of the 36 carboxylate clamp (CC)-tetratricopeptide repeat (TPR) proteins	TETRATRICOPETIDE-REPEAT THIOREDOXIN-LIKE 4 (TTL4)
At3g02170	Encodes LONGIFOLIA2 (LNG2). Regulates leaf morphology by promoting cell expansion in the leaf-length direction. The LNG2 homologue LNG1 (At5g15580) has similar function.	LONGIFOLIA2 (LNG2)
At3g54810	Encodes a protein containing a GATA type zinc finger domain that is expressed in the embryo axis and involved in germination	BLUE MICROPYLAR END 3 (BME3)/GATA TRANSCRIPTION FACTOR 8 (GATA8)
At1g32920	unknown protein	
At1g04240	SHY2/IAA3 regulates multiple auxin responses in roots	INDOLE-3-ACETIC ACID INDUCIBLE 3 (IAA3)/SHORT HYPOCOTYL 2 (SHY2)
At1g29510	SAUR-like auxin-responsive protein family.	SMALL AUXIN UPREGULATED 68 (SAUR68);
At1g21820	-	-
At4g08950	Unknown function.	EXORDIUM (EXO)
At4g11280	encodes a member of the 1-aminocyclopropane-1-carboxylate (ACC) synthase gene family.	1-AMINOCYCLOPROPANE-1-CARBOXYLIC ACID (ACC) SYNTHASE 6 (ACS6)
At1g21910	encodes a member of the DREB subfamily A-5 of ERF/AP2 transcription factor family.	DEHYDRATION RESPONSE ELEMENT-BINDING PROTEIN 26 (DREB26)
At1g29430	SAUR-like auxin-responsive protein family.	-
At4g16515	Encodes a root meristem growth factor (RGF).	ROOT MERISTEM GROWTH FACTOR 6 (RGF6)
At1g29450	SAUR-like auxin-responsive protein family.	-
At1g29500	SAUR-like auxin-responsive protein family.	-
At1g29460	SAUR-like auxin-responsive protein family.	-
At1g29440	SAUR-like auxin-responsive protein family	SMALL AUXIN UP RNA 63

		(SAUR63)
At5g25190	encodes a member of the ERF (ethylene response factor) subfamily B-6 of ERF/AP2 transcription factor family.	ETHYLENE AND SALT INDUCIBLE 3 (ESE3)
At4g27280	Calcium-binding EF-hand family protein	-
At5g52900	Encodes a member of the MAKR (MEMBRANE-ASSOCIATED KINASE REGULATOR) gene family	MEMBRANE-ASSOCIATED KINASE REGULATOR 6 (MAKR6)
At5g57560	Encodes a cell wall-modifying enzyme.	TOUCH 4 (TCH4)
At1g35140	Involved in the C-starvation response.	PHOSPHATE-INDUCED 1 (PHI-1)
At3g03830	SAUR-like auxin-responsive protein family	SMALL AUXIN UP RNA 28 (SAUR28)
**Shade up-regulated + BL down-regulated**		
**Accession #**	**Sequence Description**	**Primary Gene Symbol**
At4g32290	Core-2/I-branching beta-1,6-N-acetylglucosaminyltransferase family protein.	-
At4g28240	Wound-responsive family protein.	-
At4g27260	Encodes an IAA-amido synthase that conjugates Asp and other amino acids to auxin in vitro. Lines carrying insertions in this gene are hypersensitive to auxin. It is involved in camalexin biosynthesis via conjugating indole-3-carboxylic acid (ICA) and cysteine (Cys).	WES1
At3g19680	Protein of unknown function.	-
At2g18790	Red/far-red photoreceptor involved in the regulation of de-etiolation.	PHYTOCHROME B (PHYB)
At1g21050	Protein of unknown function.	-
At3g23030	Auxin inducible gene expressed in the nucleus.	INDOLE-3-ACETIC ACID INDUCIBLE 2 (IAA2)
At5g12050	Unknown protein.	-
At5g66590	CAP (Cysteine-rich secretory proteins, Antigen 5, and Pathogenesis-related 1 protein) superfamily protein.	-

Class S+G, shade-regulated genes (in both Col-0 and *sav3* seedlings) and GA-regulated genes.

**Shade down-regulated + GA up-regulated**		
**Accession #**	**Sequence Description**	**Primary Gene Symbol**
**Shade down-regulated + GA down-regulated**		
**Accession #**	**Sequence Description**	**Primary Gene Symbol**

Shade up-regulated + GA up-regulated		
**Accession #**	**Sequence Description**	**Primary Gene Symbol**
At3g57795	-	-
At3g02170	Encodes LONGIFOLIA2 (LNG2). Regulates leaf morphology by promoting cell expansion in the leaf-length direction. The LNG2 homologue LNG1 (At5g15580) has similar function.	LONGIFOLIA2 (LNG2)

Shade up-regulated + GA down-regulated		
**Accession #**	**Sequence Description**	**Primary Gene Symbol**
At1g54200	Unknown protein	-

Table S4. ANOVA table for experiments shown in Figure 4. DF, Degrees of Freedom; SS, Sum of Squares; MS; Mean Square.

HYP elongation in wt vs. *hfr1-5*

Source of variation	DF	SS	MS	F-value	p-value
**Genotype**	1	0.04678	0.04678	0.34	0.5604
**PIC**	2	92.75	46.38	337.56	<0.0001
**Interaction**	2	1.196	0.5978	4.35	**0.0145**
**Residual (error)**	159	21.84	0.1374	-	-

HYP elongation in wt vs. *hfr1-5*

Source of variation	DF	SS	MS	F-value	p-value
**Genotype**	1	1.148	1.148	6.49	0.0117
**EBL**	2	104.1	202.5	1143.88	<0.0001
**Interaction**	2	0.3676	0.1838	1.04	**0.3562**
**Residual (error)**	184	32.58	0.1771	-	-

HYP elongation in wt vs. G-BH.03

Source of variation	DF	SS	MS	F-value	p-value
**Genotype**	1	40.47	40.47	466.90	<0.0001
**PIC**	2	168.8	84.40	973.83	<0.0001
**Interaction**	2	8.625	4.312	49.76	**<0.0001**
**Residual (error)**	142	12.31	0.08667	-	-

HYP elongation in wt vs. G-H.02

Source of variation	DF	SS	MS	F-value	p-value
**Genotype**	1	12.54	12.54	107.16	<0.0001
**PIC**	2	210.3	105.1	898.63	<0.0001
**Interaction**	2	2.078	1.039	8.88	**0.0002**
**Residual (error)**	141	16.50	0.1170	-	-

HYP elongation in wt vs. G-BH.03

Source of variation	DF	SS	MS	F-value	p-value
**Genotype**	1	11.53	11.53	61.48	<0.0001
**EBL**	2	288.1	144.1	767.90	<0.0001
**Interaction**	2	7.791	3.896	20.76	**<0.0001**
**Residual (error)**	149	27.96	0.1876	-	-

HYP elongation in wt vs. G-H.02

Source of variation	DF	SS	MS	F-value	p-value
**Genotype**	1	7.651	7.651	54.48	<0.0001
**EBL**	2	252.5	126.2	898.89	<0.0001
**Interaction**	2	0.8391	0.4196	2.99	**0.0533**
**Residual (error)**	157	22.05	0.1404	-	-

Table S5. ANOVA table for experiments shown in Figure 5. DF, Degrees of Freedom; SS, Sum of Squares; MS; Mean Square.

HYP elongation in wt vs. PAR1-RNAi.06

Source of variation	DF	SS	MS	F-value	p-value
**Genotype**	1	6.718	6.718	63.67	<0.0001
**PIC**	2	58.96	29.48	279.36	<0.0001
**Interaction**	2	0.3180	0.1590	1.51	**0.2249**
**Residual (error)**	151	15.93	0.1055	-	-

HYP elongation in wt vs. PAR1-RNAi.06

Source of variation	DF	SS	MS	F-value	p-value
**Genotype**	1	0.02631	0.02631	0.25	0.6177
**EBL**	2	288.8	144.4	1373.92	<0.0001
**Interaction**	2	0.5986	0.2993	2.85	**0.0617**
**Residual (error)**	128	13.46	0.1051	-	-

HYP elongation in wt vs. *par2-1*

Source of variation	DF	SS	MS	F-value	p-value
**Genotype**	1	6.426	6.426	31.82	<0.0001
**PIC**	2	150.3	75.14	372.10	<0.0001
**Interaction**	2	0.3636	0.1818	0.90	**0.4084**
**Residual (error)**	166	33.52	0.2019	-	-

HYP elongation in wt vs. *par2-1*

Source of variation	DF	SS	MS	F-value	p-value
**Genotype**	1	1.975	1.975	15.51	0.0001
**EBL**	2	492.3	246.1	1933.57	<0.0001
**Interaction**	2	0.3949	0.1974	1.55	**0.2156**
**Residual (error)**	144	18.33	0.1273	-	-

HYP elongation in wt vs. PAR1-G.01

Source of variation	DF	SS	MS	F-value	p-value
**Genotype**	1	29.83	29.83	218.14	<0.0001
**PIC**	2	19.06	9.532	69.71	<0.0001
**Interaction**	2	12.71	6.356	46.48	**<0.0001**
**Residual (error)**	109	14.91	0.1367	-	-

HYP elongation in wt vs. PAR1-GG.13

Source of variation	DF	SS	MS	F-value	p-value
**Genotype**	1	31.74	31.74	277.14	<0.0001
**PIC**	2	16.36	8.181	71.43	<0.0001
**Interaction**	2	14.61	7.306	63.79	**<0.0001**
**Residual (error)**	97.0	11.11	0.1145	-	-

HYP elongation in wt vs. PAR2.12

Source of variation	DF	SS	MS	F-value	p-value
**Genotype**	1	47.58	47.58	465.09	<0.0001
**PIC**	2	16.88	8.440	82.49	<0.0001
**Interaction**	2	13.41	6.704	65.52	**<0.0001**
**Residual (error)**	102	10.44	0.1023	-	-

HYP elongation in wt vs. PAR2-G.03

Source of variation	DF	SS	MS	F-value	p-value
**Genotype**	1	51.23	51.23	503.31	<0.0001
**PIC**	2	16.11	8.055	79.14	<0.0001
**Interaction**	2	14.24	7.118	69.93	**<0.0001**
**Residual (error)**	99.0	10.08	0.1018	-	-

HYP elongation in wt vs. PAR1-G.01

Source of variation	DF	SS	MS	F-value	p-value
**Genotype**	1	24.02	24.02	186.54	<0.0001
**EBL**	2	274.4	137.2	1065.48	<0.0001
**Interaction**	2	3.250	1.625	12.62	**<0.0001**
**Residual (error)**	175	22.53	0.1288	-	-

HYP elongation in wt vs. PAR1-GG.13

Source of variation	DF	SS	MS	F-value	p-value
**Genotype**	1	3.492	3.492	24.11	<0.0001
**EBL**	2	350.0	175.0	1208.49	<0.0001
**Interaction**	2	0.2405	0.1202	0.83	**0.4376**
**Residual (error)**	175	25.34	0.1448	-	-

HYP elongation in wt vs. PAR2.12

Source of variation	DF	SS	MS	F-value	p-value
**Genotype**	1	29.47	29.47	222.19	<0.0001
**EBL**	2	222.1	111.1	837.34	<0.0001
**Interaction**	2	12.63	6.315	47.62	**<0.0001**
**Residual (error)**	173	22.94	0.1326	-	-

HYP elongation in wt vs. PAR2-G.03

Source of variation	DF	SS	MS	F-value	p-value
**Genotype**	1	9.605	9.605	65.66	<0.0001
**EBL**	2	338.0	169.0	1155.24	<0.0001
**Interaction**	2	0.3971	0.1985	1.36	**0.2601**
**Residual (error)**	174	25.45	0.1463	-	-

Table S6. ANOVA table for experiments shown in Figure 6. DF, Degrees of Freedom; SS, Sum of Squares; MS; Mean Square.

HYP elongation in wt vs. *bee123*

Source of variation	DF	SS	MS	F-value	p-value
**Genotype**	1	5.186	5.186	54.33	<0.0001
**PIC**	2	90.28	45.14	472.90	<0.0001
**Interaction**	2	2.027	1.014	10.62	**<0.0001**
**Residual (error)**	157	14.99	0.09545	-	-

HYP elongation in wt vs. *bee123*

Source of variation	DF	SS	MS	F-value	p-value
**Genotype**	1	20.51	20.51	123.16	<0.0001
**EBL**	2	266.1	133.0	798.74	<0.0001
**Interaction**	2	7.443	3.721	123.16	**<0.0001**
**Residual (error)**	144	23.98	0.1666	-	-

HYP elongation in wt vs. *bim123*

Source of variation	DF	SS	MS	F-value	p-value
**Genotype**	1	16.61	16.61	106.65	<0.0001
**PIC**	2	101.2	50.58	324.70	<0.0001
**Interaction**	2	2.245	1.123	7.21	**0.0010**
**Residual (error)**	171	26.64	0.1558	-	-

HYP elongation in wt vs. *bim123*

Source of variation	DF	SS	MS	F-value	p-value
**Genotype**	1	8.474	8.474	62.52	<0.0001
**EBL**	2	359.9	180.0	1327.69	<0.0001
**Interaction**	2	11.06	5.529	40.79	**<0.0001**
**Residual (error)**	142	19.25	0.1355	-	-

Table S7. Changes in the expression of genes reported as involved in some aspects of BR **(A)** or GA **(B)** metabolism or inactivation in response to short (1 h) or long treatments (24 h) with simulated shade. The ratio of the expression levels of the genes shown are based on data from available microarray experiments GSE9816 (Tao *et al*., 2008) and GSE8745 (Franklin and Whitelam, 2007). Gene description was obtained from TAIR (www.arabidopsis.org) database.

A. BR metabolism or inactivation.

Locus	Other Name (Type)	Description	GSE9816	GSE8745
			1 h low/high	24 h low/high (22ºC)
**At1g50430**	**DWF5**	Mutants are defective in BR biosynthesis (delta7-sterol-C7 reduction step) and have a dwarf phenotype.	1,049	0,900
**At2g36800**	**DOGT1; UGT73C5**	Encodes a DON-Glucosyltransferase. The UGT73C5 glucosylates both brassinolide and CS in the 23-O position. The enzyme is presumably involved in the homeostasis of those steroid hormones hence regulating BR activity. Transgenic plants overexpressing UGT73C5 show a typical BR-deficient phenotype.	NP	NP
**At2g26710**	**BAS1; CYP72B1; CYP734A1**	Encodes a member of the cytochrome p450 family that serves as a control point between multiple photoreceptor systems and BR signal transduction. Involved in brassinolide metabolism. Mediates response to a variety of light signals including hypocotyl elongation and cotyledon expansion.	**2,862**	**2,114**
**At2g03760**	**ATST1**	Encodes a BR sulfotransferase. In vitro experiments show that this enzyme has a preference for 24-epibrassinosteroids, particularly 24-epicathasterone, but does not act on CS and brassinolide. It is differentially expressed during development, being more abundant in young seedlings and actively growing cell cultures. Expression is induced in response to salicylic acid and methyl jasmonate and bacterial pathogens.	0,773	0,920
**At2g45400**	**BEN1**	Involved in the regulation of BR metabolic pathway	1,273	0,893
**At2g14920**	**ATST4A**	Encodes a BR sulfotransferase that may be involved in BR inactivation. In vitro experiments show that this enzyme can act on a broad group of naturally occurring BRs, including the 24-epimers and (22R,23R)-28 homobrassinosteroids, that have an array of different side chains, though it shows a preference for (22R,23R)-28 homobrassinosteroids. ST4A is expressed in the roots and transcript levels fall in response to cytokinin treatment.	0,876	**0,354**
**At2g38050**	**DET2**	Similar to mammalian steroid-5-alpha-reductase. Involved in the brassinolide biosynthetic pathway.	0,814	1,046
**At3g02580**	**DWF7**	BR biosynthetic enzyme, catalyzes delta7 sterol C-5 desaturation step. Mutant has dwarf phenotype.	1,006	1,216
**At3g13730**	**CYP90D1**	Encodes a cytochrome P-450 gene that is involved in BR biosynthesis, most likely in the conversion step of teasterone (TE) to 3-dehydroteasterone (3DT), and/or 6-deoxoteasterone (6-deoxoTE) to 6-deoxo-3-dehydroteasterone (6-deoxo3DT); or the conversion of cathasterone (CT) to TE, and/or 6-deoxocathasterone (6-deoxoCT) to 6-deoxoTE. Recently, CYP90D1 was shown to catalyse the C-23 hydroxylation of several BRs (the enzyme has a broad specificity for 22-hydroxylated substrates). Member of the CYP90C CYP450 family. Similar to Cytochrome P450 90C1 (ROT3).	1,116	0,872
**At3g19820**	**CBB1; DWF1**	Involved in the conversion of the early BR precursor 24-methylenecholesterol to campesterol. BRs affect cellular elongation. Mutants have dwarf phenotype. DWF1 is a Ca2+-dependent	1,032	1,030

		calmodulin-binding protein.		
**At3g30180**	**BR6OX2; CYP85A2**	Encodes a cytochrome p450 enzyme that catalyzes the last reaction in the production of brassinolide. It is capable of converting 6-deoxocastasterone into CS, a C-6 oxidation, as well as the further conversion of CS into brassinolide by a Baeyer-Villinger oxidation reaction at C-6, resulting in the formation of an unusual seven-membered lactone ring. The enzyme possesses high affinity for both C28- and C27-BRs. The expression of the gene using a CYP85A2 promoter:LUC fusion construct was shown to be under circadian and light control.	1,474	1,819
**At3g50660**	**DWF4; SAV1**	Encodes a 22α hydroxylase whose reaction is a rate-limiting step in BR biosynthetic pathway. The protein is a member of CYP90B gene family. CLM is an epi-allele with small, compressed rosette, reduced internode length, and reduced fertility, appears in selfed ddm mutant plants possibly due to loss of cytosine methylation. Transcripts accumulate in actively growing tissues, and GUS expression is negatively regulated by BRs. Localized in the endoplasmic reticulum. The in vitro expressed protein can perform the C-22 hydroxylation of a variety of C27-, C28- and C29-sterols. Cholesterol was the best substrate, followed by campesterol. Sitosterol was a poor substrate.	1,275	1,935
**At4g36380**	**ROT3**	Encodes a cytochrome P-450 gene that is involved in leaf blade expansion by controlling polar cell expansion in the leaf length direction. Member of the CYP90C CYP450 family. ROT3 was shown to be involved in brassinosteroid biosynthesis, most likely in the conversion step of typhasterol (TY) to CS. As 6-deoxo-CS was unable to restore the phenotype of rot3-1, it has been postulated that ROT3 might be specifically involved in the conversion of TY to CS in the C6-oxidation pathway of brassinolide. Recently, CYP90C1 was shown to catalyse the C-23 hydroxylation of several BRs (the enzyme has a broad specificity for 22-hydroxylated substrates).	0,773	0,628
**At5g05690**	**CPD; DWF3**	Encodes a member of the CP90A family, a cytochrome P450 monooxygenase which converts 6-deoxocathasterone to 6-deoxoteasterone in the late C6 oxidation pathway and cathasterone to teasterone in the early C6 oxidation pathway of brassinolide biosynthesis. Expressed in cotyledons and leaves. Mutants display de-etiolation and derepression of light-induced genes in the dark, dwarfism, male sterility and activation of stress-regulated genes in the light. The expression of the gene using a CPD promoter:LUC fusion construct was shown to be under circadian and light control. Additionally, the circadian regulation was shown to be independent of BR levels as it remains unchanged in *bri1* mutant lines. CPD appears to be involved in the autonomous pathway that regulates the transition to flowering, primarily through a BRI1-mediated signaling pathway that affects FLC expression levels, as uncovered by double mutant analyses.	1,243	1,483
**At1g17060**	**CYP72C1; SOB7**	Encodes a protein with similarity to other cytochrome P450's and is a homolog of BAS1. Over expression causes a dwarf phenotype resembling brassinolide resistant mutants. Double mutant analysis of sob7/bas1 loss of function mutants suggests these genes have redundant functions in light responsiveness. SOB7 may function in metabolizing brassinolides. Expressed in leaf, root, stem and silique but expression highest in flower and cauline leaves. Dominant overexpressing plants have dwarf phenotype, short siliques/seeds, rounded dark green leaves and short hypocotyls in light and dark. Loss of function alleles result in plants with long hypocotyls.	1,135	1,442
**At5g38970**	**BR6OX1; CYP85A1**	Encodes a polypeptide involved in the C-6 oxidation of BRs. Heterologous expression of the protein in yeast conferred the ability to catalyze multiple reactions in which the C-6 position of 6-deoxocastasterone, 6-deoxotyphasterol, 3-dehydro-6-deoxoteasterone and 6-deoxoteasterone are oxidized.	0,595	**0,282**
**At1g50430**	**DWF5**	Mutants are defective in BR biosynthesis (delta7-sterol-C7 reduction step) and have a dwarf phenotype.	1,049	0,900
**Loss-of-function mutants have a DWARF phenotype - defective in BR biosynthesis**				
**When overexpressed, plants have a DWARF phenotype - metabolism of active BRs**				
**not clearly defined whether involved in BR metabolism or inactivation.**				

B. GA metabolism or inactivation.

Locus	Other Name (Type)	Description	GSE9816 1h low/high	GSE8745 24h low/high (22ºC)
**At4g25420**	**GA20ox1**	Encodes a GA 20 oxidase	**5,41**	**8,31**
**At5g51810**	**GA20ox2**	Encodes a GA 20 oxidase	**4,11**	**3,91**
**At5g07200**	**GA20ox3**	Encodes a GA 20 oxidase	0,66	**4,56**
**At1g60980**	**GA20ox4**	Encodes a GA 20 oxidase	1,36	**3,06**
**At1g44090**	**GA20ox5**	Encodes a GA 20 oxidase	0,54	**3,67**
**At1g15550**	**GA3ox1**	Encodes a GA 3 oxidase	1,83	0,90
**At1g80340**	**GA3ox2**	Encodes a GA 3 oxidase	1,22	0,89
**At4g21690**	**GA3ox3**	Encodes a GA 3 oxidase	**0,21**	**6,15**
**At1g80330**	**GA3ox4**	Encodes a GA 3 oxidase	1,08	1,56
**At1g78440**	**GA2ox1**	Encodes a GA 2 oxidase	1,16	1,00
**At1g30040**	**GA2ox2**	Encodes a GA 2 oxidase	1,05	1,30
**At2g34555**	**GA2ox3**	Encodes a GA 2 oxidase	1,12	1,20
**At1g47990**	**GA2ox4**	Encodes a GA 2 oxidase	0,58	0,56
**At1g02400**	**GA2ox6**	Encodes a GA 2 oxidase	1,12	0,63
**At1g50960**	**GA2ox7**	Encodes a GA 2 oxidase	0,94	**0,21**
**At4g21200**	**GA2ox8**	Encodes a GA 2 oxidase	1,20	1,48
**Encode enzymes involved in metabolism (production) of active GAs**				
**Encode enzymes Involved in catabolism (inactivation) of active GAs**				
